# Versatile Assays for High Throughput Screening for Activators or Inhibitors of Intracellular Proteases and Their Cellular Regulators

**DOI:** 10.1371/journal.pone.0007655

**Published:** 2009-10-30

**Authors:** Hideki Hayashi, Michael Cuddy, Vincent Chih-Wen Shu, Kenneth W. Yip, Charitha Madiraju, Paul Diaz, Toshifumi Matsuyama, Muneshige Kaibara, Kohtaro Taniyama, Stefan Vasile, Eduard Sergienko, John C. Reed

**Affiliations:** 1 Burnham Institute for Medical Research, La Jolla, California, United States of America; 2 Nagasaki University Graduate School of Biomedical Sciences, Sakamoto, Nagasaki, Japan; 3 Conrad Prebys Center for Chemical Genomics, Burnham Institute for Medical Research, La Jolla, California, United States of America; Roswell Park Cancer Institute, United States of America

## Abstract

**Background:**

Intracellular proteases constitute a class of promising drug discovery targets. Methods for high throughput screening against these targets are generally limited to in vitro biochemical assays that can suffer many technical limitations, as well as failing to capture the biological context of proteases within the cellular pathways that lead to their activation.

**Methods & Findings:**

We describe here a versatile system for reconstituting protease activation networks in yeast and assaying the activity of these pathways using a cleavable transcription factor substrate in conjunction with reporter gene read-outs. The utility of these versatile assay components and their application for screening strategies was validated for all ten human Caspases, a family of intracellular proteases involved in cell death and inflammation, including implementation of assays for high throughput screening (HTS) of chemical libraries and functional screening of cDNA libraries. The versatility of the technology was also demonstrated for human autophagins, cysteine proteases involved in autophagy.

**Conclusions:**

Altogether, the yeast-based systems described here for monitoring activity of ectopically expressed mammalian proteases provide a fascile platform for functional genomics and chemical library screening.

## Introduction

Proteolytic processing of proteins is an irreversible post-translational modification of importance for a wide-variety of biological processes, including cell division, cell death, cell differentiation, innate immunity, host-pathogen interactions, and intracellular protein sorting and trafficking. Consequently, proteases have emerged as promising targets for drug discovery for a wide variety of human diseases, including cancer, neurodegeneration, ischemic diseases, inflammation, and infectious diseases. Development of high throughput screening (HTS) assays using purified proteases can be relatively straightforward or it can be quite challenging, particularly when multi-component systems are required to achieve protease activation. Also, due to similarity of the active sites of some groups of proteases, selectivity of chemical inhibitors can be difficult if not impossible to achieve, highlighting the need for alternative screening methods for identifying compounds that target upstream activators of proteases rather than directly inhibiting the protease of interest.

Caspases represent an excellent example of a family of intracellular endopeptidases for which novel HTS tools are desired. Caspases are cysteine proteases that are conversed throughout the animal kingdom. The human genome contains at least 10 genes encoding Caspases (reviewed in [1]). These proteases often collaborate in complex proteolytic networks that encompass upstream initiators and downstream efforts, where upstream members of the Caspase family cleave and activate downstream members. Upstream initiator Caspases become activated through protein interactions involving assembly of multi-protein complexes (reviewed in [2], [3]), which are often difficult to reconstitute in vitro. The substrates cleaved by active Caspases are responsible for apoptotic cell death and for cytokine-mediated inflammation, thus making these proteases attractive targets for drug discovery for a wide variety of degenerative diseases, ischemic disease.

Autophagy is an evolutionarily conserved process for catabolizing organelles and macromolecules during times of nutrient insufficiency for the purpose of generating substrates for maintaining ATP production (reviewed in [4]). Autophagy is recognized as a cell survival mechanism in many contexts of relevance to human physiology and disease, though it also may contribute to non-apoptotic cell death under some circumstances [5]. Autophagins are intracellular cysteine proteases required for autophagy [6]. The human genome contains four genes encoding autophagy proteases, which include ATG4A, ATG4B, ATG4C, and ATG4D [7].

We created yeast-based cellular systems that permit facile expression of proteases and protease-activating proteins in combinations that reconstitute entire mammalian pathways in these simple eukaryotes. Among the assay methods integrated into the yeast system are cleavable reporter gene activators, in which protease-mediated cleavage activates a transcription factor. We demonstrate here the utility of this approach for caspases and autophagins. Applications of this technology to high throughput chemical library screening and to cDNA library screening demonstrate its broad applicability.

## Results

### Development and testing of a cleavable transcription factor and reporter gene system for assaying protease activity in living yeast

**Figure 1:**
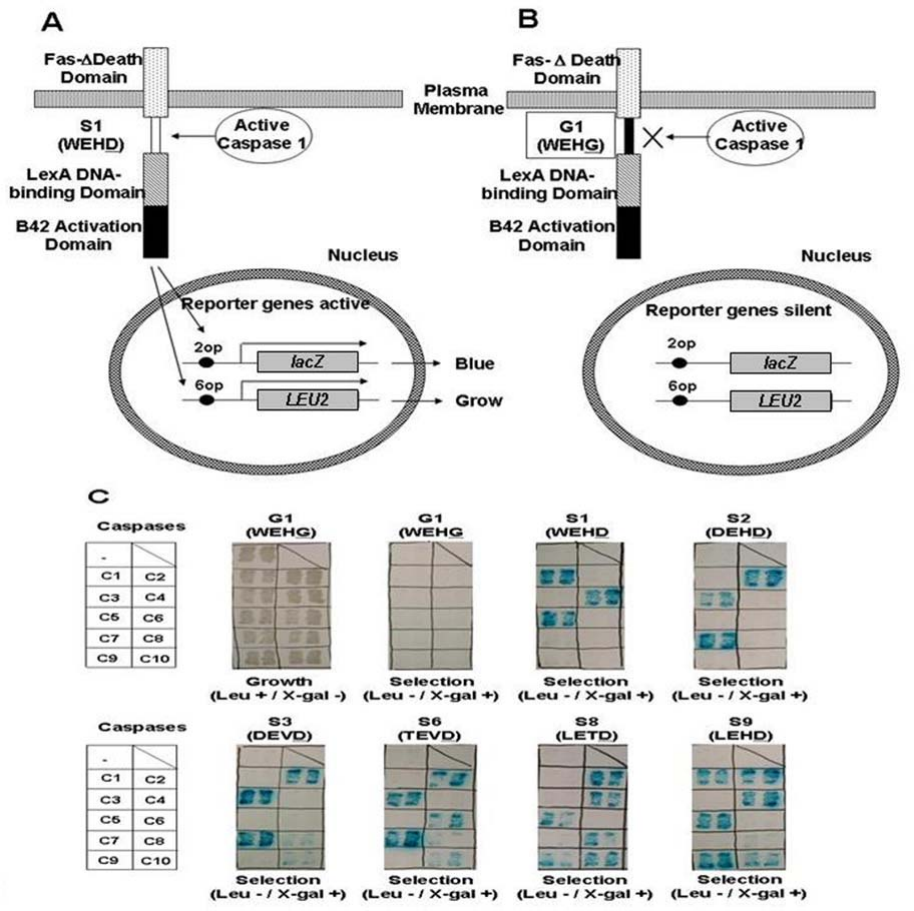
A genetic system for monitoring Caspase-1 activity in yeast cells. (A) Engineered yeast were created that express a type-1 transmembrane protein (Fas-d-S1-TA) in which the Fas devoid of the death domain (Fas-d) is followed by a Caspase-1 target site (S1-WEHD), and a transcriptional activator (TA-consisting of LexA DNA binding domain and B42 activation domain). *lexA* operators are located upstream of *lacZ* (2 operators) and *LEU2* (6 operators) reporter genes, respectively. The cells expressing 6op-*LEU2*/2op-*lacZ*/TEF-Fas-d-S1-TA stimulate Caspase-1 activity reporter, because expression of active Caspase-1 (over-expressing a full-length pro-Caspase-1 construct) results in Fas-d-S1-TA cleavage at the Caspase-1 target site (S1), releasing the transcriptional activator (TA), which enters the nucleus and activates *lacZ* and *LEU2* reporter gene transcription. (B) A version of Fas-d-S1-TA in which the P_1_ aspartate is changed to glycine (Fas-d-G1-TA) cannot be cleaved by active Caspase-1. The cells expressing 6op-*LEU2*/2op-*lacZ*/TEF-Fas-d-G1-TA in which the glycine substitution is found serve as false-positive reporters for molecules that activate *lacZ* and *LEU2* gene expressions independent of cleavage at the Caspase-1 target site. (C) *Substrate specifities of the caspases expressed in yeast*. Plasmids encoding various cleavable transcription factor substrates, harboring different tetrapeptide cleavage sequences optimized for specific Caspases (Fas-d-S1-TA, Fas-d-S2-TA, Fas-d-S3-TA, Fas-d-S6-TA, Fas-d-S8-TA, and Fas-d-S9-TA) were created by substituting the Caspase-1 cleavage site (S1-WEHD) with the Caspase-2 cleavage site (S2-DEHD), the Caspase-3 cleavage site (S3-DEVD), the Caspase-6 cleavage site (S6-TEVD), the Caspase-8/Caspase-10 cleavage site (S8-LETD), and the Caspase-9 cleavage site (S9-LEHD), respectively, and were expressed with the *lacZ* and *LEU2* reporter genes. A construct encoding a Fas-LexA/B42 transcription factor containing a pseudo-site (G1) with the non-cleavable sequence WEHG was also generated. The resultant yeast strains EGY48, expressing 6op-*LEU2*/2op-*lacZ*/TEF-Fas-d-G1-TA, 6op-*LEU2*/2op-*lacZ*/TEF-Fas-d-S1-TA, 6op-*LEU2*/2op-*lacZ*/TEF-Fas-d-S2-TA, 6op-*LEU2*/2op-*lacZ*/TEF-Fas-d-S3-TA, 6op-*LEU2*/2op-*lacZ*/TEF-Fas-d-S6-TA, 6op-*LEU2*/2op-*lacZ*/TEF-Fas-d-S8-TA, or 6op-*LEU2*/2op-*lacZ*/TEF-Fas-d-S9-TA, were transformed with Caspase expression plasmids. Substrate specificities were determined for the ten Caspases (C1–C10). If cells express the active Caspases with the suitable cleavage sites, the cells can grow in the selection medium (without leucine) and they hydrolyze X-gal to become blue. Expression of the Caspases (p424-ADH-Caspase1-FLAG, p424-ADH-HA-Caspase2, p424-ADH-Caspase3, p424-TEF-Caspase4, p424-ADH-Caspase5, p424-TEF-HA-Caspase6, p424-ADH-Caspase7, p424-CYC1-Caspase8-HA/TEF-HA-FADD, p424-ADH-HA-Caspase9-FLAG, and p424-CYC1-Caspase10-FLAG/TEF-HA-FADD) had no effects on the cell growth when plated on regulate (leucine-containing) medium.

We devised a reporter gene system for monitoring protease activity in living yeast, modeled after prior reports that used the same concept [8], [9]. For this purpose, a Type I transmembrane receptor (CD95; Fas) was expressed such that most of its cytosolic domain was replaced by a chimeric transcription factor comprised of the DNA-binding domain of LexA and the transactivation domain of B42. Between Fas and the chimeric transcription factor (LexA-B42) we cloned various numbers of tetrapeptide sequences known to be recognized and cleaved by various members of the Caspase family. As a control, constructs were prepared in which the sessile aspartic acid within the tetrapeptide sequence was replaced with glycine (Figure 1A, B). These reporter constructs were expressed from plasmids in autotrophic yeast mutant strains EGY48 (*MATα trp1*, *his3*, *ura3*, *lexA*6op-*LEU2*) or EGY191 (*MATα trp1*, *his3*, *ura3*, *lexA*2op-*LEU2*), containing stably integrated *LEU2* (allows grown on leucine-deficient media only when the operator is activated by the transcriptional activator, LexA-B42) and episomal *lacZ* (produces β-galactosidase) reporter gene under control of promoters containing 1, 2, 4, 6 or 8 copies of the LexA-binding operator [10].

Caspases are initially produced as inactive zymogens, which typically undergo proteolytic processing to produce active enzymes composed in most cases of tetrameric assemblies with two large (∼20 kDa) and two small (∼10 kDa) subunits. Simply over-expressing Caspases in yeast is sufficient to lead to their activation in many cases [11], [12], [13], [14], presumably because upstream initiator Caspases are typically activated by dimerization and also because many Caspases are capable of cleaving and activating their zymogen forms, constituting a feed-forward mechanism for amplifying protease signaling (reviewed in [15]). Moreover, expressing high levels of some Caspases results in killing of yeast or growth suppression [11], [12], [13], [14], [16], thus interfering with use of a cleavable transcription factor and reporter gene strategy for monitoring changes in Caspase activity. The key to applying reporter gene-based systems for evaluating Caspase activity in yeast thus is to carefully titrate levels of expression of these proteases so that expression is sufficiently high to achieve activation but insufficient to kill yeast or impair their growth.

To accomplish this goal, we empirically evaluated several yeast promoters and also produced attenuated versions of some promoters to titrate Caspase expression to appropriate levels. We expressed full-length Caspases-1, 2, 3, 4, 5, 6, 7, and 9 from high copy-number plasmids using either ADH or TEF promoters, which are constitutive promoters with moderate to strong activity. The levels of Caspase expression afforded by the ADH and TEF promoters were sufficient to achieve ‘spontaneous” activation of these over-expressed proteases, but insufficient to kill yeast or impair their growth. For Caspases-8 and 10, however, we co-expressed a small amount of the full-length (zymogen) proteins using the CYC promote (a constitutive promoter with relatively weak activity) together with a large amount of the upstream activator, FADD (an adapter protein that contains a Death Effector Domain (DED) that binds the DEDs found in the prodomains of Caspase-8 and -10 [17], because large amounts of active Caspases-8 or -10 inhibited the yeast cell growth significantly, consistent with prior reports [18], [19]. Using a *ΔLEU2* yeast strain containing *LEU2* and *lacZ* reporter genes, active Caspases were co-expressed with cleavable transcription factors containing various tetrapeptide target sequences. Pilot experiments with different numbers of cleavage sites in the membrane-tethered transcription factor (n = 1−6) suggested that a single tetra-peptide Caspase cleavable sequence yields satisfactory results. We empirically adjusted variables such as the strengths of the yeast promoters driving expression of Caspases and the Fas-LexA-B42 cleavable fusion proteins and the type of DNA replication origin in the plasmids (high copy versus low copy) to optimize signal:noise ratio, so that background (spontaneous) activation of the *LEU2* and *lacZ* reporter genes was minimal (not shown), while also titrating the number of *lexA* operators in the reporter genes to ensure a signal well above background.

When plated on complete medium, all *leu2* transformants grew, as expected (Figure 1C). However, a selective pattern of growth was observed for yeast transformants when they were plated on leucine-deficient medium to test activity of the LexAop-*LEU2* reporter gene, along with X-gal substrate to colorimetrically detect the presence of β-galactosidase activity. In yeast containing the WEHG control sequence, which lacks the sessile aspartic acid required for Caspase cleavage, none of the yeast transformants grew or showed β-galactosidase-positivity, including Caspase-1 through Caspase-10. Yeast in which the Fas-LexA-B42 fusion protein contains the WEHD tetrapeptide sequence grew on leucine-deficient media and showed β-galactosidase-positivity when co-expressed with active Caspases-1, 4, and 5, but not Caspases-2, 3, 6, 7, 8, 9, or 10, perfectly matching predictions of prior studies in which the optimal tetrapeptide substrate sequences were determined for these human Caspases [20]. Similarly, yeast containing the DEVD linker in the Fas-LexA-B42 fusion protein grew on leucine-deficient media and showed β-galactosidase-positivity when co-expressed with active Caspase-3 or Caspase-7, but not Caspases-1, 4, 5, 6, 8, 9, or 10, showing striking preferentiality among Caspases for cleavage of this reporter protein. Caspase-2 also cleaved the DEVD-containing reporter protein, which differs only slightly from the reported optimal substrate sequence of DEHD and was reported to be second-most efficiently cleaved tetrapeptide sequence for this protease [20]. The DEHD substrate (designed for Caspase-2) produced results very similar to DEVD, showing activation by Caspases-2, -3, and -7, consistent with prior substrate specificity experiments suggesting that the P_2_ position where H was placed is the most flexible residue for Caspases-3 and -7 substrates [20]. The other tetrapeptide sequences optimized for Caspase-6 (TEVD), Caspases-8/10 (LETD) and Caspase-9 (LEHD) resulted in less specific patterns of reporter protein activation, but nevertheless showed selectivity. For example, the “inflammatory” Caspases (Caspases-1, 4, and 5) did not activate the Caspase-6 substrate (TEVD). Also, the downstream effector Caspases (Caspases-3, 6, 7) did not activate the reporter proteins containing tetratpeptide sequences optimized for upstream initiator Caspases (LETD, Caspases-8/10; LEHD, Caspase-9). In contrast, the *lacZ* reporter gene was not activated when substituting non-cleavable reporter constructs in which the aspartic acid in position P1 of the cleavable linker was converted to glycine or replacing vectors encoding wild-type Caspases with mutants in which the active site cysteine was changed to glycine or alanine (*Figure S1*). Taken together, these data demonstrate the performance of a one-component cleavable reporter gene system for measuring activity of all 10 human Caspases in yeast.

We have employed this system for screening human cDNA libraries expressed from yeast plasmids, where the cleavable Fas-LexA-B42 fusion protein was expressed without a Caspase and cDNA libraries were screened for proteases that activate the reporter gene (*Figures S2–S4*). The libraries employed here were constructed by directional cloning into a high copy-number yeast plasmid containing GAL1 or ADH promoters (strong and moderate strength, respectively) for driving cDNA expression. Using the WEHD-containing reporter protein, we obtained eleven cDNAs that confirmed positive on repeated testing, including five that encoded Caspase-1 and six encoding Caspase-4 (*Figures S2 and S4*). Using the DEVD-containing reporter, we obtained twelve clones that confirmed positive, including three that encoded Caspase-3 and nine encoding Caspase-7 (*Figures S3 and S4*). These cDNA library screening results further validate the cleavable reporter system.

### Development of 2-component systems demonstrating function of Caspase activators in yeast

**Figure 2:**
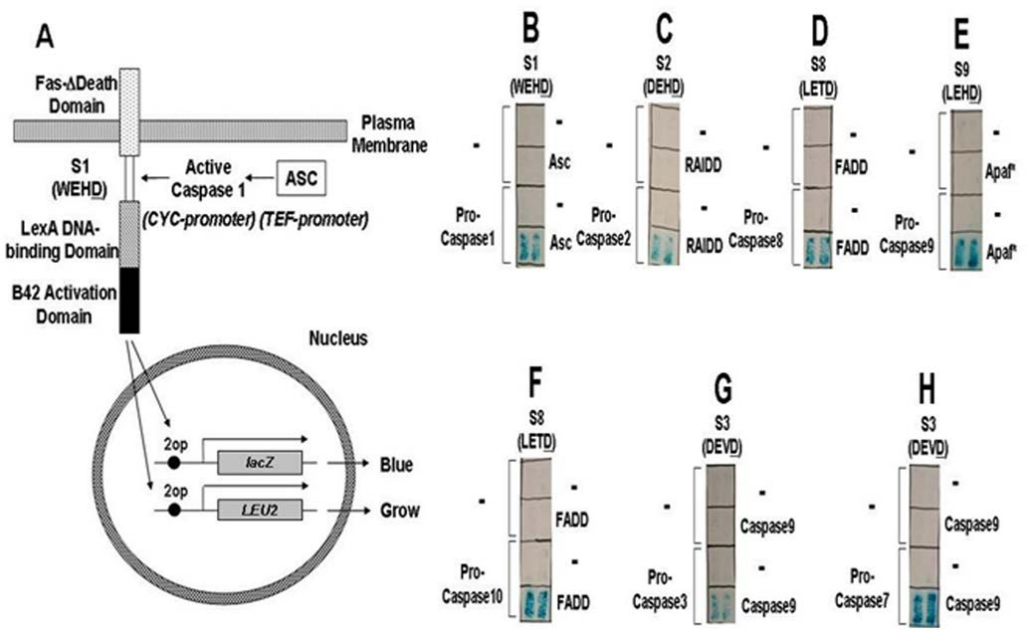
Schematic representation of a 2-component system for caspase1 activators in yeast cells. (A) The zymogen (inactive) pro-caspase-1 protein is expressed in yeast, with the cleavable reporter containing the S1 site (WEHD), by regulating the expression level (p413-TEF-Fas-d-S1-TA/ΔTEF1-Caspase1-FLAG) in the yeast cell (EGY191 expressing 2op-*LEU2*/2op-*lacZ*). The reporter genes are silent in the absence of Asc. Co-expression of Asc at high levels from p424-TEF-HA-Asc activates Caspase-1, resulting in S1 site cleavage, releasing the transcription factor from the membrane to enter the nucleus and activate reporter genes. Co-expression of Asc at high levels from p424-TEF-HA-Asc activates Caspase1, resulting in S1 site cleavage, and the transcription factor is released from the membrane to enter the nucleus and activate reporter genes. (B) Yeast transformants were plated on leucine-deficient medium containing X-gal. For controls, the “empty” version of the plasmid was always introduced so that cells were subjected to identical selection conditions. The recipient yeast strain EGY191 containing 2op-*LEU2*/2op-*lacZ* with substrate plasmid TEF-Fas-d-S1-TA, with or without a gene encoding pro-Caspase-1 (ΔTEF1-Caspase1-FLAG) were transformed with p424-TEF-HA-Asc or empty vector (-). Only the combination of Caspase-1 plus Asc resulted in reporter gene activation, indicating that Asc itself did not cut the S1 site. (C) Experiments were performed as above, except the recipient yeast strain EGY48 containing *6op-LEU2/2op-lacZ* with substrate plasmid ΔTEF2-Fas-d-S2-TA, with or without a gene encoding pro-Caspase-2 (ΔGPD1-HA-Caspase2-FLAG), was transformed with p424-TEF-HA-RAIDD or empty vector (-). (D) The recipient yeast strain EGY48 containing *6op-LEU2/2op-lacZ* with substrate plasmid GPD-Fas-d-S8-TA, with or without a gene encoding pro-Caspase-8 (CYC1-Caspase8-HA), was transformed with p424-TEF-HA-FADD or empty vector (-). (E) The recipient yeast EGY48 containing *6op-LEU2/2op-lacZ* with substrate plasmid TEF-Fas-d-S9-TA, with or without a gene encoding pro-Caspase-9 (TEF-HA-Caspase9), was transformed with p424-TEF-HA-Apaf* expressing an active mutant of Apaf-1 or empty vector (-). (F) The recipient yeast strain EGY48 containing *6op-LEU2/2op-lacZ* with substrate plasmid GPD-Fas-d-S8-TA, with or without a gene encoding pro-Caspase-10 (ADH-Caspase10-FLAG), was transformed with p424-TEF-HA-FADD or empty vector (-). (G, H) The recipient yeast EGY191 containing *2op-LEU2/2op-lacZ* with substrate plasmid ΔTEF3-Fas-d-S3-7-TA, with or without genes encoding pro-Caspase-3 (ΔCYC2-Caspase3) or the recipient yeast EGY48 containing *6op-LEU2/2op-lacZ* with substrate plasmid TEF-Fas-d-S3-7-TA, with or without genes encoding pro-Caspase-7 (CYC1-Caspase7), was transformed with p424-ADH-HA-Caspase-9-FLAG or empty vector (-).

Next, we attempted to develop 2-component systems for assaying Caspase activity in yeast, in which the inactive proforms of the Caspases were co-expressed with various activator proteins and in which the Caspases were expressed from weak promoters to achieve levels of the zymogens that avoid “spontaneous” activation. Figure 2 depicts the concept for Caspase-1, showing its preferred substrate WEHD. Among the 2-component systems interrogated were combinations of upstream initiator Caspases co-expressed with known activators, including: (1) pro-Caspase-1 plus Asc; (2) pro-Caspase-2 plus RAIDD; (3) pro-Caspase-9 plus a gain-of-function mutant of Apaf1; (4) pro-Caspase-8 plus FADD; and (5) pro-Caspase-10 plus FADD. In each of these cases, the upstream initiator Caspase, contains a N-terminal pro-domain (either CARD or DED) that binds a compatible CARD or DED in the activator protein. We also tested 2-component systems involving an upstream and downstream protease. For example, Caspase-9 is a direct upstream activator of downstream proteases, Caspases-3 and -7 [21]. We therefore over-expressed active Caspase-9 (high level expression) in combination with the inactive proforms of Caspase-3 or -7 (expressed at low levels) (Figures 2G, 2H, S5). In each case, an appropriate cleavable reporter protein was co-expressed with the 2-component systems in yeast containing *LEU2* and *lacZ* reporter genes. For all pair-wise combinations of pro-Caspase and upstream activator, we expressed the pro-Caspase at relatively low levels using a low-copy plasmid containing CEN/ARS (1 to 2 copies per cell) and using weak to moderate strength promoters (such as CYC1, and ΔGPD1), and the upstream activator at high levels using 2µ plasmid origin-containing plasmids (20 to 50 copies per cell) with relatively strong promoters (TEF or ADH). For each pair of pro-Caspase and upstream activator, we empirically adjusted the number of *lexA* operators driving the *LEU2* or *lacZ* reporter genes to achieve an acceptable signal:noise ratio. For example, for the combination of pro-Caspase-1 and Asc, we determined that 2 *lexA* operators for *LEU2* were superior to 4 or 6 operators when using ΔTEF1-Caspase1-FLAG to express pro-Caspase-1 (Figure 2), and that 6 *lexA* operators for *LEU2* were superior to 2 or 4 operators when using ΔTEF3-Caspase1-FLAG to express pro-Caspase-1 (Figures S6A and S7).

Successful activation of the cleavable reporter protein was achieved for each 2-component system (Figure 2). Expression of either the pro-Caspase alone or the upstream activator alone failed to result in activation of the *LEU2* and *lacZ* reporter genes, as determined by ability to grow on leucine-deficient plates and β-galactosidase-positivity, confirming that both components are necessary for activating the reporter proteins. Expressing Caspases in which the active site cysteine was mutated to glycine or alanine failed to activate the cleavable reporter (*Figures S1, S5, and S6*). Moreover, pairing pro-Caspases with the wrong activators (e.g. Apaf1*+pro-Caspase-8; Asc+pro-Caspase-9) also failed to activate the reporters (*Figure S7*). Taken together, these data demonstrate the utility of yeast-based protease screening using 2 component systems.

### Development of a 3-component system – mammalian protease activating pathways reconstituted in yeast

**Figure 3:**
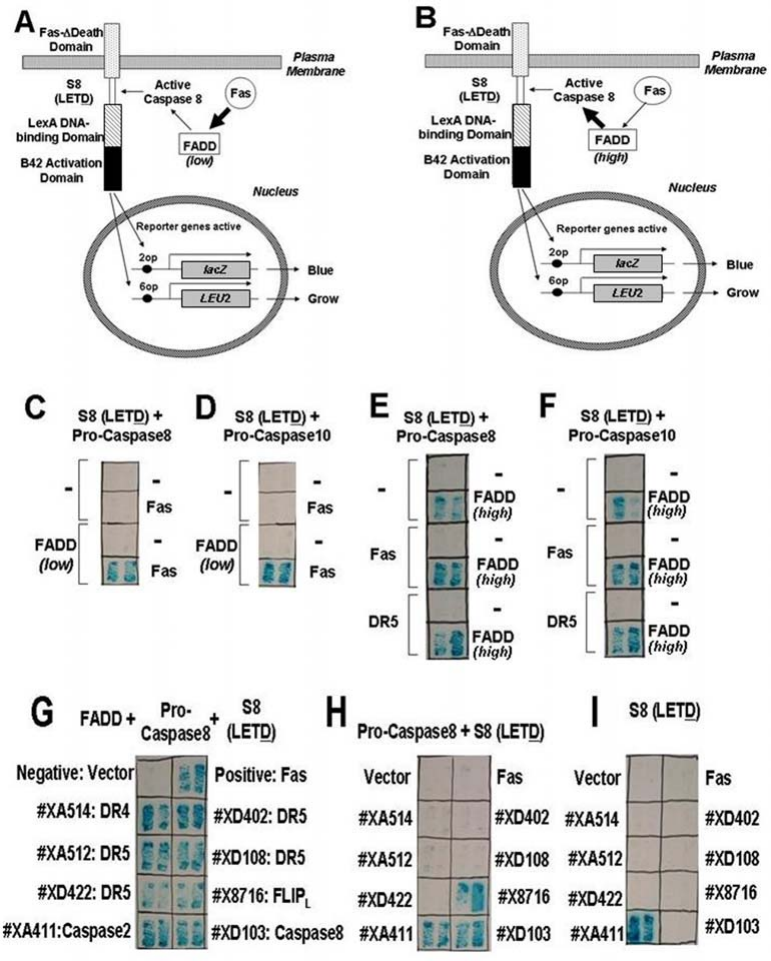
3-component systems for Caspase-8 activation in yeast. The zymogen pro-Caspase-8 is expressed (from CYC1-promoters) with substrate containing LETD cleavage element S8 (from plasmid p413-TEF-Fas-d-S8-TA) in the yeast EGY48 expressing 6op-*LEU2*. (A) A small amount of FADD is also expressed from ΔADH1 promoter on a low-copy plasmid without activating the Caspase-8. Fas is expressed at high levels from ADH promoter on a high copy plasmid. (B) FADD and Fas are both expressed at high levels from TEF and ADH promoters, respectively, from high copy plasmids. (C,D) To validate the system from (A) for cloning cDNAs that activate Caspase-8 or -10 in a FADD-dependent manner (the death receptor cloning system), we established yeast transformants expressing Caspase-8 or -10, with low levels of FADD or corresponding empty vectors (-). Then, the cells (FADD/Caspase-8 or -10) were transformed with a Fas-expressing vector (p424-ADH-Fas) or the vector only (-). The yeast transformants included EGY48-6op-*LEU2*/2op-*lacZ*/TEF-Fas-d-S8(LETD)-TA/CYC1-Caspase8-HA (C) and EGY48-6op-*LEU2*/2op-*lacZ*/GPD-Fas-d-S8(LETD)-TA/CYC1-Caspase10-FLAG (D), without (-) or with a small amount of FADD, and were transformed with the a Fas-expressing vector or the empty vector (-). (E,F) To test the system from (B), The yeast transformants included EGY48-*6op-LEU/2op-lacZ*/TEF-Fas-d-S8 (LETD)-TA/CYC1-Caspase8-HA (E), and EGY48-6op-*LEU2*/2op-*lacZ*/GPD-Fas-d-S8(LETD)-TA/CYC1-Caspase10-FLAG (F) without (-) or with large amounts of Fas or DR5, and were transformed with the a FADD-expressing vector in a large amount under TEF prompter or the empty vector (-). (G–I) Examples of cDNA cloning results. Yeast strain EGY48 containing 6op*-LEU2* and 2op-*lacZ* reporter genes and expressing the LETD-containing substrate (expressed from pTEF-Fas-d-S8-TA) with or without pro-Caspase-8 (CYC1-Caspase8-HA), and/or FADD (ΔADH1-FADD). Cells were subsequently transformed with various cDNA libraries (*Figs. S9–S11*) and clones that activated the reporter genes were characterized by recovery of cDNA library plasmids and retransformation into yeast expressing FADD and pro-Caspase-8 (G), pro-Caspase-8 without FADD (H), or neither (I). Among the positive clones were #XA514 (n = 13) encoding a fragment of DR4 (G^210^-E^468^), #XA512 (n = 15) encoding a fragment of DR5 (A^43^-S^411^), #XD108 (n = 8) encoding a fragment of DR5 (Y^99^-S^411^), and #XD422 (n = 3) encoding a fragment of DR5 (A^192^-S^411^), and #XD402 (n = 7) encoding a fragment of DR5 (V^124^-S^440^), all of which activated the *lacZ* reporter gene in cells containing FADD and Caspase-8 (G) but not in cells lacking FADD (H) or both (I). Also obtained were cDNAs #XD103 encoding a fragment of Caspase-8 (M^1^-R^233^), #X8716 encoding a full-length FLIP_L_, and #XA411 encoding a full-length Caspase-2. Assays were performed in duplicate, growing cells for 4 days on plates.

Having developed prototypical 2-component systems, we attempted to increase the complexity of reconstituted Caspase-activating systems in yeast, moving to a 3-component network. Two different mammalian Caspase-activating networks were explored. In the first, we attempted to recapitulate the proximal portion of the extrinsic pathway (so-called “death inducing signaling complex” [DISC]), expressing (1) death domain (DD)-containing TNF-family death receptor, Fas [CD95]; (2) bipartite adapter protein FADD, which contains DD and DED; and (3) the proform of a DED-containing protease, either Caspase-8 or Caspase-10, along with (4) a cleavable reporter protein containing the Caspase-8/10 substrate tetrapeptide LETD. Two strategies for reconstituting the Fas/FADD/pro-Caspase-8 network in yeast were compared. First, we tried expressing the bridging adapter protein FADD at low levels using a constitutive but weak promoter such that the amount of FADD was inadequate to achieve pro-Caspase-8 activation in the absence of Fas. For that system, Fas was expressed at high levels so that it can oligomerize with itself without requiring Fas Ligand (Figure 3A). By empirically comparing different strength promoters for driving expression of the 3 components and adjusting the number of *lexA* operators in the promoters of the *LEU2* and *lacZ* reporter genes, we arrived at conditions that produced the desired result for the approach whereby low levels of constitutive FADD were complemented by expression of Fas (Figure 3C, 3D, *S8*). Second, we expressed low levels of either pro-Caspase-8 or pro-Caspse-10 with high levels of adapter protein FADD (Figure 3B), adding expression plasmids encoding death receptors such as Fas (CD95) or DR5 (TRAIL-R2) to this system. However, high FADD was sufficient to activate Caspase-8 and -10, with addition of death receptors only modestly further increasing reporter gene activity (Figures 3E, F).

**Figure 4:**
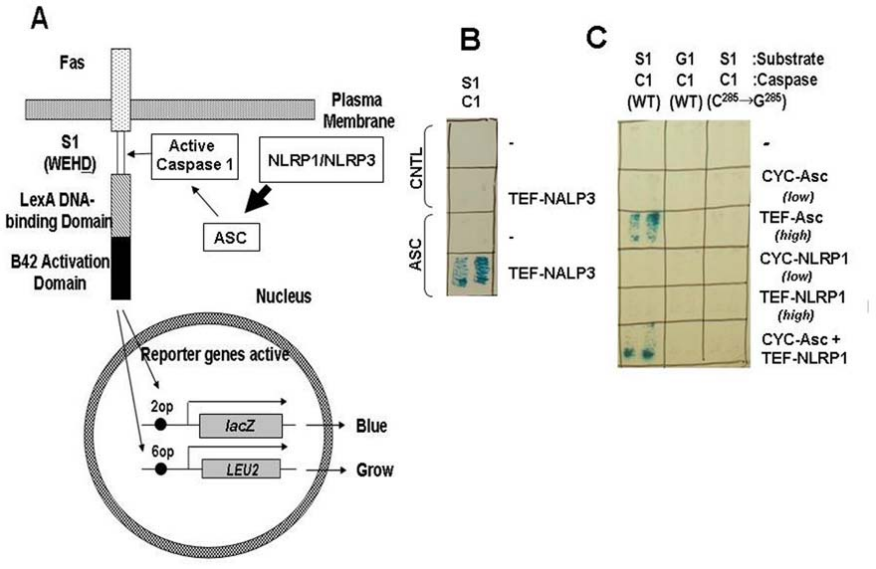
Yeast assay for NLRs. (A) Yeast strain EGY48 containing 6op-*LEU2/*2op*-lacZ* expressing pro-Caspase-1 (driven by ΔTEF3 promoter, which expresses at low levels) with membrane tethered transcription factor substrate containing WEHD peptide linker (S1 substrate) were transformed with plasmids encoding adapter protein Asc (expressed at low levels from a CYC promoter) and upstream activators either NLRP1ΔLRR or NLRP3ΔLRR (expressed at high levels from TEF or GAL1 promoters). When Caspase-1 is activated via the combination of Asc and NLRP1 or NLRP3, the reporter is cleaved, releasing the chimeric LexA/B42 transcription factor to leave the membrane and enter the nucleus, where it induces expression of *LEU2* and *lacZ* genes. (B) EGY48 cells (*6op-LEU2*, *2op-lacZ*) were transformed with plasmids encoding the S1 substrate, pro-Caspase-1 (C1) expressed from ΔTEF3 promoter, Asc *(low)* from CYC promoter, and NLRP3ΔLRR from TEF promoter. Control transformants received the corresponding empty plasmids (-). Cells were grown for 2 days on leucine deficient plates containing X-gal and galactose (to induce NLRP3ΔLRR expression). (C) The EGY48 recipient (6op-*LEU2*, 2op-*lacZ*) yeast cell strain contained plasmids encoding substrates with either the WEHD tetrapeptide cleavable linker (S1 substrate expressed from TEF-Fas-d-S1(WEHD)-TA transcriptional unit) or WEHG non-cleavable substrate (G1 substrate expressed from TEF-Fas-d-G1(WEHG)-TA) in plasmids encoding either wild-type (“C1/WT”) or Cys285 mutated Caspase-1 (“C1/C^285^→G^285^”) (expressed from ΔTEF3-Caspase-1-FLAG or ΔTEF3-Caspase-1(C^285^→G^285^)-FLAG transcriptional units). Cells were transformed with the plasmids expressing a small amount of Asc (CYC-Asc), a large amount of Asc (TEF-Asc), a small amount of NLRP1ΔLRR (CYC-NLRP1), a large amount of NLRP1ΔLRR (TEF-NLRP1), various combinations, or empty vectors (-). Yeast transformants were plated on leucine-deficient medium containing X-gal. The large amount of Asc (expressed from TEF promoter) activated Caspase-1 by itself. NLRP1ΔLRR was unable to activate Caspase-1 by itself, albeit expressed at high levels (confirmed by immunoblotting [*not shown*]). NLRP1ΔLRR activated pro-Caspase-1 in the presence of a small amount of Asc, under conditions where the amount of Asc expressed (from CYC promoter) was insufficient to independently activate Caspase-1.

Another 3 component Caspase-activating network was similarly created for NLR-family proteins, using variations on the same approach. NLRs constitute a family of signaling proteins involved in innate immunity, several of which activate pro-inflammatory Caspases (reviewed in [22]). NLRs are activated by pathogen-derived ligands via leucine-rich repeat (LRR) domains that auto-inhibit these proteins in the absence of ligand, controlling oligomerization of these proteins that form multiprotein complexes termed “inflammasomes” [23]. We attempted to reconstitute in yeast the NLRP1 (NALP1) and NLRP3 (NALP3) inflammasome comprising 3-component systems including: (1) the CARD-containing protease, pro-Caspase-1; (2) the bipartite adapter Asc, which contains CARD and PYD domains; and (3) the NLR-family protein NLRP1/NALP1 (which contains PYD and CARD domains) or NLRP3/NALP3 (which contains PYD domain), using gain-of-function mutants of NLRP1 and NLRP3 that are constitutively active without requiring a bacterial ligand to induce their oligomerization by virtue of deletion of the auto-inhibitory LRRs [23]. For this system, the reporter protein contained the Caspase-1 cleavage sequence, WEHD (Figure 4). We then expressed pro-Caspase-1 and adapter protein Asc in yeast under constitutive weak promoters, such that the amount of Asc produced was insufficient to activate pro-Caspase-1 in the absence of other cofactors. The gain of function mutants, NLRP1(ΔLRR) and NLRP3(ΔLRR) were expressed under control of either strong inducible (GAL1) or constitutive (TEF) promoters. Upon NLRP1(ΔLRR) expression, the reporter genes became activated. Exclusion of any of the 3 components of this system (NLRP1, Asc, or pro-Caspase-1) prevented the reporter gene activation (not shown). Substituting the WEHD-containing cleavable reporter construct with non-cleavable WEHG and substituting wild-type Caspase-1 with mutant Caspase-1 in which the active site cysteine was ablated failed to result in activation of the *LEU2* or *lacZ* reporter genes (Figure 4C and not shown). We conclude therefore that NLRP1ΔLRR and NLRP3ΔLRR can activate pro-Caspase-1 in yeast in an Asc-dependent manner, thus demonstrating reconstitution in yeast of another 3-component system for Caspase activation.

Having developed 3-component systems for Caspase activation, we next exploited this technology for screening cDNA expression libraries. Several strategies were successfully employed to explore the diversity of applications using the Fas/FADD/Caspase-8 system (reconstituted DISC). For example, cDNA cloning experiments were performed where we omitted the plasmid encoding Fas from the 3-component system, leaving FADD (low levels), Caspase-8 (low levels), and the cleavable reporter, then screened cDNA libraries prepared in yeast expression plasmids with moderate strength promoters (typically ADH promoters), searching for cDNA clones encoding proteins that restored activation of the cleavable reporter gene containing the LETD target sequence. In this application, the yeast cell line, EGY48-6op-*LEU2*/2op-*lacZ*/GPD-Fas-d-S8/10(LETD)-TA/CYC1-Caspase8-HA/ΔADH1-FADD was used screen to cDNA libraries in search of clones encoding death receptors that activate Caspase-8 in a FADD-dependent manner. From a screen of 1.7×10^6^ cDNAs from a HepG2 hepatocellular library, 28 of 40 positive clones tested encoded DR4 (TRAIL Receptor-1) or DR5 (TRAIL Receptor-2) (Figure 3E–G and *supplemental Figure S9*), members of the TNF-family of death receptors that are known to activate Caspase-8 via FADD [24], [25], [26], [27]. These DR4 and DR5-encoding cDNA clones failed to restore reporter gene activation when FADD was omitted, showing their dependence on this adapter protein for inducing activation of Caspase-8 (Figure 3E, F). A second independent screening of the same HepG2 library, plating 1.2×10^6^ clones, resulted in 25 positive colonies, of which 8 encoding DR5 (Figure 3E–G and *supplemental Figure S9*). Also, screening 7.5×10^5^ clones from a human liver cDNA expression library resulted in 18 positives, of which 10 encoded DR5 (Figure 3E–G and *supplemental Figure S10*). In another cDNA library screening experiment, 5×10^5^ clones from a HeLa cervical carcinoma cDNA library were screened, resulting in 19 positives, of which 7 encoded a splice-variant of Caspase-8. This cDNA clone activated the reporter gene independently of FADD (Figure 3H) but interestingly not when eliminating Caspase-8 (Figure 3I), thus illustrating how the heirarchical functions within pathways of cDNA hits can be readily deconvoluted by omission of elements of the 3-component system. Similar examples of cDNA clones encoding downstream activators of the Fas/FADD/Caspase-8 pathway were found in a screen of 2.2.×10^6^ clones from a HEK293 renal epithelial cell line library, which resulted 16 positive clones, of which 2 encoded c-FLIP (Figure 3G, *S11*), a Caspase-8-like protein that is known to activate Caspase-8 in some contexts [28], and 9 clones encoded Caspase-2 (Figure 3G, *S11*). The c-FLIP cDNA activated the cleavable reporter independently of FADD but required Caspase-8 (Figures 3H, I), as expected [28], while the Caspase-2 clone activated the reporter independently of both FADD and Caspase-8 (Figure 3H, I), suggesting it directly cleaves the reporter protein containing the LETD sequence. In this regard, while the preferred cleavage sequence for Caspase-2 has been reported as DEHD, our comparisons of Caspase-2 on various cleavable reporters showed that LETD is also a substrate (Figure 1, S8), consistent with results of peptide library screens in which L at the P_4_ position was shown to be cleaved by Caspase-2 with ∼40% the efficiency as D [20]. In another example of how this 3-component system can be employed for functional cloning, we expressed low levels pro-Caspase-10 and high levels of Fas, then screened cDNA libraries to clone adaptor molecules that link Fas to Caspase-10 (*Figures S12–S15*). The yeast cell line EGY48-6op-*LEU2*/2op-*lacZ*/GPD-Fas-d-S8/10(LETD)-TA/CYC1-Caspase10-FLAG/ADH-Fas failed to activate Caspase-10 in the absence of FADD, as monitored by activation of the *LEU2* and *lacZ* reporter genes. In contrast, Caspase-10 was activated when FADD was expressed in these engineered yeast cells (*Figure S13*). Screening 2.2×10^6^ clones from a HEK293 cell library resulted in 55 positives, of which 24 encoded FADD (*Figures S14, S15*). These and other cDNA library screening experiments demonstrated the robust performance of the cleavable reporter-based screening system for studying proteases, illustrated here for the DISC (Fas/FADD/Caspase-8/10).

### Adaptation of the yeast-based Caspase activation assay to HTS

We have implemented the yeast-based protease reporter system in HTS compatible formats by revising conditions from agar plates to liquid medium in microtiter wells. To this end, we measured β-galactosidase produced by yeast carrying the Caspase-cleavable reporter proteins, assaying the colorimetric product (OD_620 nm_) derived from X-gal substrate in 384 well plates, as a measure of the *lacZ* reporter gene activity. Comparisons were made of β-galactosidase activity produced in the presence or absence of the broad-spectrum Caspase inhibitory compound, zVAD-fmk (benzoyl-Valinyl-Alaninyl-Aspartyl-fluoromethylketone). Among the variables that were initially interrogated were initial seeding cell density, time of culture, concentration of X-gal substrate, and supporting carbohydrate (raffinose vs mannose) (*Figures S16 and S17*). Note that we cannot employ glucose in yeast media because it represses the *GAL1* promoter used in some plasmids. All variables tested alter the signal to noise ratio of the assay. For the Fas/FADD/Caspase-8 multi-component assay, the best results were achieve with ∼2×10^5^ cells per well starting density cultured for ∼3 days in raffinose-containing media with 400 ug/mL concentration of X-gal substrate. For the 2-component system of Asc/Caspase-1, the same conditions were optimal, among those tested (not shown). For the NLRP1ΔLRR/Asc/Caspase-1 multi-component assay, the best results were achieved with ∼2×10^5^ cells per well starting density cultured for ∼3 days in raffinose-containing media with 400 µg/mL concentration of X-gal substrate.

**Figure 5:**
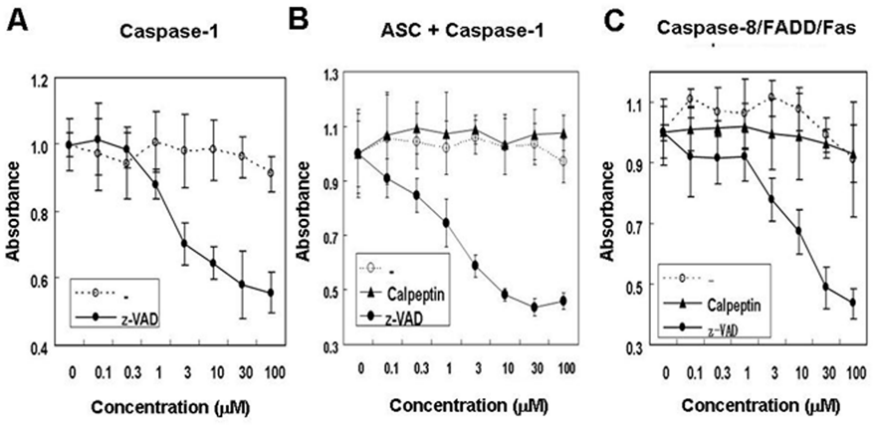
Validation of yeast-based assay using pharmacological inhibitor of Caspases. (A) *Caspase-1*, *single component assay*. EGY48 yeast cells expressing S1 substrate (TEF-Fas-d-S1-TA) and high levels of Caspase-1 (TEF-HA-Caspase1-FLAG) were incubated for 3 days in selection media (SD/X-gal) with the indicated concentrations of z-VAD-fmk in DMSO (•) or DMSO only (○), using 384-well plates. (B) *Asc/Caspase-1 two-component system*. EGY48 yeast cells expressing S1 substrate (TEF-Fas-d-S1-TA) a low level of pro-Caspase-1 (ΔTEF1-Caspase1-FLAG) and high levels of Asc (TEF-HA-Asc) were incubated for 3 days in the selection media (SD/X-gal) in 384 well plates with the indicated concentrations of calpeptin (▴) or z-VAD-fmk (•) or an equivalent volume of DMSO (○). (C) *Fas/FADD/Caspase8 three-component system*. EGY48 yeast cells expressing S8 substrate (TEF-S8-TA), low levels of pro-Caspase-8 CYC1-Caspase-8-HA, with a small amount of FADD (from ΔADH1 promoter) and a large amount of Fas (from ADH promoter), were incubated in selection media (SD/X-gal) with the indicated concentrations of calpeptin (▴) or z-VAD-fmk (•), or equivalent volume of DMSO (○), in 384-well plates for 3–4 days. For (A–C), relative absorbance at 620 nm was measured (mean ± std dev; n = 3−5 independent experiments). (* indicates p<0.001).

We further validated the 384 well assay format, using zVAD-fmk, a peptidyl inhibitor with broad-spectrum activity against animal Caspases. For some experiments, a Calpain inhibitor was employed in side-by-side experiments as a control. The broad-spectrum Caspase inhibitor zVAD-fmk inhibited β-galactosidase activity in a dose-dependent fashion, with the concentration required for achieving 50% inhibition ranging from ∼1–10 µM in these assays, which were performed in 384 well format (Figure 5) (*Table S1*). In contrast, the Calpain inhibitor did not suppress activation of the *lacZ* reporter gene. These experiments verify that the 384 well version of the yeast-based Caspase-dependent reporter gene assay faithfully indicates the activity of an inhibitory compound, thus fulfilling one of the prerequisites for developing HTS assays.

**Figure 6:**
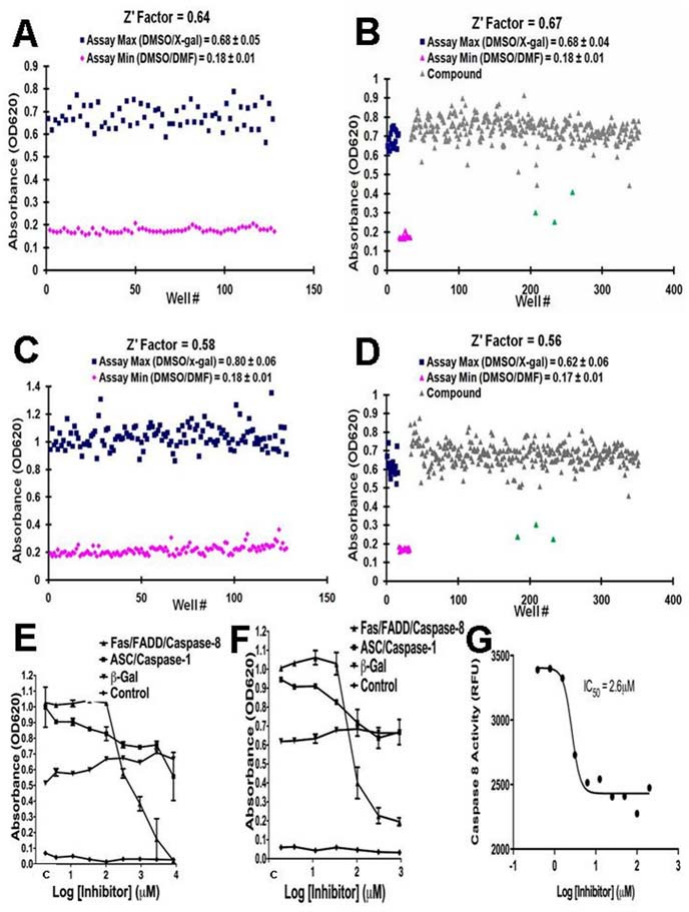
HTS Implementation of yeast-based, 3-component Caspase assays. (A) *Validation of HTS assay for NLRP1-Asc-Caspase-1 system*. Yeast cells (18 µl, typically at ≈2×10^5^ cells/well) containing NLRP1ΔLRR/Asc/Caspase-1 were plated into wells of 384 well plates in media containing 1% galactose, 0.2% raffinose. Wells also contained 18 µL selection media with (blue squares) or without (purple diamonds) X-gal substrate (400 µg/mL final Concentration) plus 10% DMSO (4 µl) (to simulate compound addition). The plates were incubated for 72 hrs at 30°C and the OD_620_ was recorded. Values (mean±std dev) were calculated for the assay maximum and minimum. The Z' factor was calculated as described [45]. (B) *Example of library screening plate for NLRP1-Asc-Caspase-1*. Yeast cells (typically at ≈2×10^5^ cells/well) containing NLRP1/Asc/Caspase-1 were plated in 384 well plates in Selection Media, without (purple triangles) or with (all other wells) 400 µg/ml X-gal (final concentration). Compounds in DMSO (∼10 µM final) had been pre-added to wells (diamonds). The plates were incubated for 72 hrs at 30°C and the OD_620_ was recorded, “Hits” were defined by >50% reduction (as shown in green diamonds). (C) *Validation of HTS assay for Fas-FADD-Caspase-8 system*. Yeast cells containing Fas/FADD/Caspase-8 were cultured in 384 well plates as above with (blue squares) or without (purple diamonds) X-gal substrate, the plates were incubated for 72 hrs at 30°C and analyzed as above. (D) *Example of library screening plate for Fas-FADD-Caspase-8*. Yeast cells containing Fas/FADD/Caspase-8 were plated at ≈2×10^5^ cells/wells in 384 well plates in Selection Media, without (purple triangles) or with (all others) 400 µg/ml X-gal (final concentration), and without (triangles, squares) or with (diamonds) compounds in 10% DMSO (∼10 µM final). Plates were incubated for 48 hrs at 30°C and OD_620_ values recorded. “Hits” were defined by >50% reduction (shown as green diamonds). (E,F) *Examples of compounds showing evidence of selective inhibition of Fas-FADD-Caspase-8*. EGY48 yeast cell lines employed for deconvoluting hits included yeast expressing *lacZ* gene (▾) expresses LexA/B42 transcription factor from GAL1 promoter; cells grown in galactose media to induce promoter) to eliminate false-positives due to β-galactosidase inhibition and Asc/Caspase-1-expressing yeast (▪) to eliminate hits that interfere with a different Caspase. Yeast containing empty vector were also included (⧫). Yeast cells (at 2×10^5^ cells/well) containing Fas/FADD/Caspase-8 (▴) or other strains were cultured in 384 well plates in a total volume of 40 µL Selection Media, with 400 µg/ml X-gal final concentration, without (“C”) or with various concentrations of compound CID-5154 or CID-3101. The plates were incubated for 2–4 days at 30°C and OD_620_ values recorded. (G) *Example of compound showing evidence of inhibition of Caspase-8*. An in vitro biochemical assay was used containing purified recombinant Caspase-8 and fluorigenic substrate Ac-IETD-AFC. Data represent relative fluorescence units (RFU) assessed in the absence or presence of various concentrations of compound (CID-2531).

To assess the reproducibility of the 384 well microplate assay, we prepared multiple replicate wells in 384 well plates representing the negative (assay max) and positive (assay min) controls for the assays. For the 3-component systems of Fas/FADD/Caspase-8 and NLRP1ΔLRR/Asc/Caspase-1, the maximum signal was produced by cells grown with X-gal substrate, while the minimum was set by either cells grown without X-gal substrate (Figure 6) or by adding ZVAD-fmk (*Figure S18*). For both assays, Fas/FADD-mediated activation of Caspase-8 and NLRP1ΔLRR/Asc-mediated activation of Caspase-1 resulted in activation of the cleavable transcription factor, producing β-galactosidase, which was measured using a colorimetric substrate. We empirically adjusted the yeast cell density, incubation time, and other variables to format a 384 well assay. The Z' factors for the Fas/FADD/Caspase-8 assay and the NLRP1ΔLRR/Asc/Caspase-1 assay were determined by reading multiple replicates of the assay max and min, and determined be >0.6 and >0.6, respectively, and thus suitable for HTS (Figure 6A, 6C and *S18*).

We then performed automated pilot screens of 1280 compounds (LOPAC library) using the Fas/FADD/Caspase-8 and NLRP1ΔLRR/Asc/Caspase-1 assay, achieving an average hit rate of ∼0.5 and ∼0.5 per 384 well plate, respectively, which is quite acceptable particularly given that the library is enriched in bioactive molecules. Examples of primary screening results are shown (Figures 6B, D). We further evaluated the 8 hits obtained using the Fas/FADD/Caspase-8 screen using a combination of engineered yeast cells carrying vectors designed for post-HTS compound profiling and using in vitro enzymatic assays for β-galactosidase and Caspase-8. The yeast cell lines employed for deconvoluting hits included yeast constitutively expressing *lacZ* gene to eliminate false-positives due to β-galactosidase inhibition and Asc/Caspase-1 expressing yeast to eliminate hits that interfere with a different Caspase or its upstream activator, in addition to original Fas/FADD/Caspase-8-expressing yeast. Of 8 hit compounds, 4 inhibited β-galactosidase activity in yeast and in vitro, representing false-positives. Two of the compounds showed selectivity for Fas/FADD/Caspase-8 over Asc/Caspase-1, though displaying some inhibition of the latter in the yeast-based reporter gene assay at higher concentrations (Figures 6E, F). One of the compounds was confirmed to be a direct inhibitor of Caspase-8 using a biochemical assay with fluorigenic peptide substrate, showing concentration-dependent inhibition of this protease with IC50 of ∼2.6 µM (Figure 6G). Thus, these yeast-based HTS assays are suitable for compound library screening.

### Application of yeast-based assay system for Autophagins

**Figure 7:**
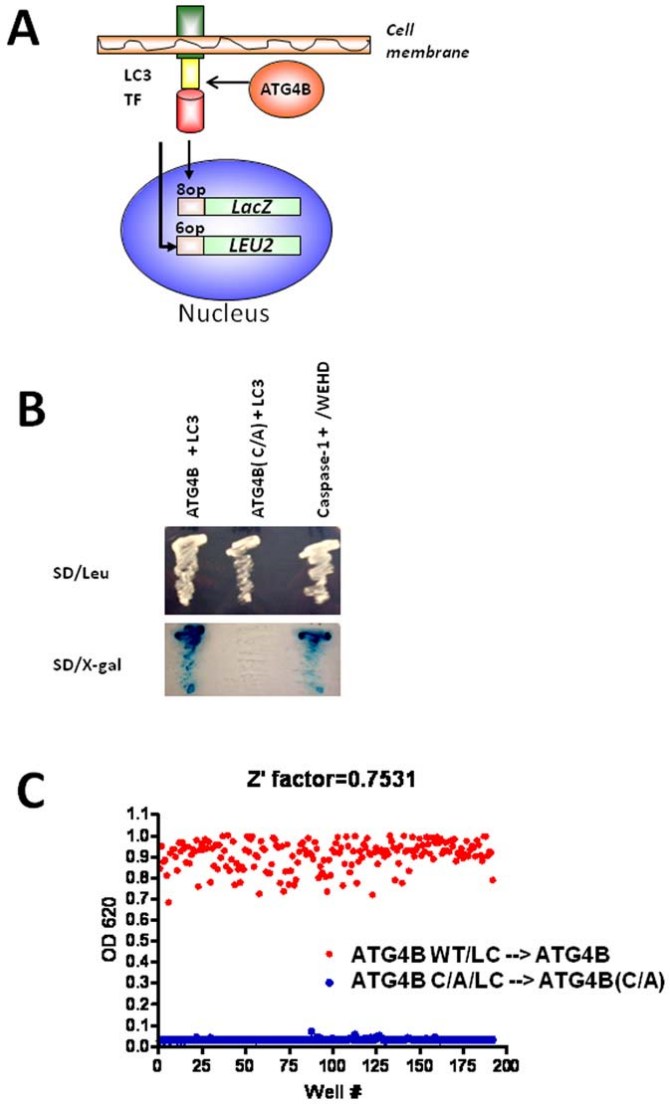
Yeast-based HTS assay for ATG4B. (A) The basis for the yeast-based cleavable reporter assay for monitoring ATG4B activity is depicted. The membrane tethered transcription factor (TF) consists of the DNA-binding domain of the LexA protein and the transactivation domain of the B42 protein, fused to LC3 and the extracellular and transmembrane domains of Fas. ATG4B cleaves LC3 to release the chimeric LexA/B42 transcription factor, leaving the membrane and entering the nucleus, where it induces expression of *LEU2* and *LacZ* genes. (B) EGY48 yeast transformed with plasmids encoding various proteases (ATG4B, ATG4B(C/A), Caspase-1) and various Fas-LexA/B42 substrates containing either LC3 protein or the WEHD tetrapeptide were streaked on standard media plates without (top) or with (bottom) Xgal substrate. Note that wild-type ATG4B but not mutant ATG4B(C/A) activated the *lacZ* reporter gene. (C) For HTS implementation, multiple replicate wells were prepared of yeast cells (100 µL/well at 10^4^ cells/mL) expressing LC3-containing substrate and either ATG4B (red) or ATG4B(C/A) mutant (blue) in 384 well plates using media containing 1% galactose/2%raffinose, with X-gal substrate. Cells were cultured for 48 hrs at 30°C before recording OD 620 nm values.

To explore the general utility of the yeast-based system for assaying mammalian proteases, we turned to another class of intracellular cysteine proteases, Autophagins. These proteases are required for autophagy, with the human genome containing genes encoding four family members – ATG4A, ATG4B, ATG4C, and ATG4D [7]. The substrate specificity of Autophagins has been partially determined, with glycine required at the point of hydrolytic cleavage [29]. A physiological substrate of mammalian Autophagins is the protein LC3. We therefore prepared a cleavable, transmembrane fusion protein containing the LC3 protein fused between the membrane tethering Fas (CD95) protein fragment described above and the chimeric LexA/B42 transcription factor. The Autophagin family member ATG4B or a mutant in which the catalytic cysteine was converted to alanine (C/A) was expressed from a TEF inducible plasmid in yeast with the engineered LC3 substrate (Figure 7A). Culturing the transformed cells on X-gal plates showed that the LexA-reponsive *lacZ* reporter gene was activated upon expression of ATG4B but not the ATG4B(C/A) mutant (Figure 7B). Substituting Caspase-1 for ATG4B also failed to activate the reporter gene (not shown). To explore the utility of the Autophagin assay for HTS, we adapted it to the 384 well format by optimizing cell density and incubation time (*Figure S19*). Multiple replicate wells were prepared with EGY48 strain yeast expressing plasmids encoding ATG4B (maximum signal) or ATG4B(C/A) mutant (minimum signal), showing robust assay performance (Z' factor>0.7) (Figure 7C). Thus, the technology described here can be adapted to additional classes of intracellular protease, as evidenced by these experiments using ATG4B.

## Discussion

Proteases play important roles in a wide variety of cellular processes and diseases. Proteases can be found in either intracellular or extracellular (or cell surface) locations, where they encounter their specific substrates. Among the intracellular families of proteases are Caspases, Autophagins, Calpains, Deubiquitinating enzymes (DUBs), Presenilins, and Separases. Still other proteases may have dual intracellular and extracellular lives. For example, Cathepsins are normally stored in lysosomes undergoing vesicular recycling between extracellular and intracellular compartments, but also are released into the cytosol under various pathological circumstances [30]. Moreover, many clinically significant viruses encode proteases that are candidate targets for drug discovery.

From the standpoint of high-throughput screening (HTS) for identifying chemical inhibitors of proteases, many endopeptidases (or at least their catalytic domains) can be readily produced in large quantities by recombinant DNA technology, purified, and formatted for HTS using convenient fluorigenic or colorimetric substrates. However, such assay formats have several limitations. First, standard HTS configurations for proteases are typically amenable only to screens for inhibitors, not activators of proteases. Second, assays requiring large amounts of purified, active protease can be hampered by difficulties in producing by recombinant methods or purifying from endogenous sources sufficient amounts of material, as well as by instability problems where purified proteases lose activity in vitro. Third, because one is often limited to using only the catalytic domain due to difficulties of expressing and purifying intact full-length proteases, opportunities to discover allosteric modulators of proteases are limited, with most of the standard assays favoring identification of compounds that target the active site in a competitive fashion. Fourth, when using single target systems consisting of purified protease or catalytic domain of protease, opportunities for identification of compounds that target other proteins involved in protease activation are lost. Fifth, several classes of intracellular endopeptidases exists as large families of closely related enzymes with structurally very similar active sites, making it difficult to achieve selective inhibitors in the absence of more context-dependent, multi-component systems that would provide the opportunity for selective inhibition. Sixth, some proteases are not active in purified form, requiring necessary cofactors (e.g. Separins involved in chromosome segregation and cell division) or requiring membranes (e.g. Presenilins [γ-secretase] involved in Amyloid-beta-peptide processing).

For these and other reasons, it would be desirable to have cell-based alternatives to in vitro biochemical assays for protease HTS. Our goal was to develop adaptable approaches for reconstituting in yeast protease activation pathways and networks, using Caspases as a prototype. The HTS implementation of complex 3-component protease networks described here illustrates the robust performance of the configured assays, in which we carefully titrated levels of various components by (a) using promoters of different strengths, (b) employing low versus high copy yeast plasmids; and (c) utilizing reporter genes with different numbers of transcription factor binding sites in their promoters. Acceptable Z' factors were achieved (>0.5) in 384 well format, and proof of concept screens of a small library enriched in bioactive compounds demonstrated acceptable hit rates. Post-HTS deconvolution of hit compounds is readily achieved by using yeast engineered with progressively reduced complexity (reporter gene only, protease only, etc.) to reduce or eliminate false-positive compounds that directly inhibit the reporter gene or its product (β-galactosidase) and to differentiate compounds that inhibit the protease versus upstream activators of the protease. Selectivity of compounds is also readily interrogated by using various Caspase assays described here as counter-screens. In this regard, we succeeded to reconstitute in yeast all ten human Caspases with cleavable transcription factor reporters, thus covering the entire family of these proteases. We also demonstrated the versatility of the technology by configuring an assay for autophagins.

Mammalian and invertebrate Caspases have been expressed in yeast previously for basic research purposes but configuration of yeast-based Caspase assays suitable for HTS have not been described heretofore. A cleavable membrane-tethered transcription factor was previously described by Hawkins and Hay, and used to define the substrate specificity of an invertebrate Caspase (Ced3) and to explore the activities of endogenous inhibitors of Caspases (IAP family proteins) [8], [9]. Similarly, a cDNA library cloned into a membrane tethered transcription factor was used for functional cloning of Caspase substrates in yeast [31]. By exploiting the ability of over-expressed Caspases to kill yeast, and thus using cell death as an endpoint (rather than a reporter gene strategy), reconstitution of Caspase activation pathways has been previously achieved in yeast for the 3 component network of Apaf1/Caspase-9/Caspase-3 [32], [33] and the 2 component networks of Ced4/Ced3 [34], [35], Caspase-2/Caspase-7 [11], Caspase-8/Caspase-3, and Caspase-10/Caspase-3 – in some cases together with animal or viral protein inhibitors of these proteases [12], [13], [19]. In another application, yeast cells have been modified to express a viral protease (TEVP) together with an engineered substrate that releases an endonuclease (DFF4) to kill yeast [36]. Also, a 4-component system has been reconstituted in yeast for γ-secretase activity, comprised of Presenlin-1, Nicastrin, APH-1, and PEN-2, using *lacZ* (β-galactosidase) reporter gene activation by a membrane-tethered cleavable transcription factor (Amyloid β-protein precursor [APP] fused to GAL1 transcription factor) as an endpoint for assessing activity of this transmembrane protease complex [37]. The suitability for HTS of these other systems in which heterologous proteases were reconstituted in yeast has not been demonstrated. A mammalian cell assay based on endogenous secretase-mediated cleavage of an APP-Gal4-VP16 fusion protein that activates a luciferase reporters gene has been implemented as a HTS screen [38], but has the limitation that more than one endogenous mammalian protease can cleave this engineered substrate.

The yeast-based assays for proteases described here were also successfully exploited for functional cDNA library screening, in addition to compound library screening. By using two sequential selections based on *LEU2* (growth on leucine-deficient media) and *lacZ* (blue color on X-gal plates) reporter gene activity, false-positive clones were reduced, resulting in a high proportion of cDNAs that were clearly true-positives (124 of 276 total cDNA hits tested from 11 separate cDNA library screening experiments). Moreover, some of the cDNA hits that we called false-positive may with further investigation prove to actually be genuine regulators of the Caspases activation pathways used for screening. With the exception of screen for substrates of proteases [31], we are unaware of prior reports that successfully used cleavable transcription factor/reporter gene read-outs for functional cDNA library screening in yeast or other types of eukaryotic cells for Caspases or other types of intracellular proteases. Cell death has been successfully employed as an end-point for screening a cDNA library in search of protein inhibitors of Caspases, where cDNA clones that rescued yeast from a Caspase-mediated lethality were identified [8]. Reporter gene-based screens and lethality-based screens presumably each introduce different types of bias, and could even be used in tandem to efficiently reduce false-positives in functional cDNA library screening projects. Similarly, reporter gene-based and lethality-based screens of Caspases in yeast could be applied in tandem for compound library screening, using various colorimetric and fluorigenic indicators of yeast cell viability for the latter in microtiter plate format. However, not all Caspases [18] and not all animal or viral proteases ectopically expressed in yeast produce lethality or growth inhibitory phenotypes, thus precluding the use of viability/growth rescue as a general strategy for functional cDNA library screening or compound library screening.

In summary, we described versatile expression vectors that provide a range of levels of expression of heterologous proteins in yeast, plasmids encoding a transcription factor substrate with a cleavable linker to accommodate various protease specificities, and methods for configuring complex multi-component protease activation pathways for high throughput chemical library screening. These unique assay components and methods were demonstrated for all ten human Caspases and for a human autophagin (ATG4B). The described technology platform thus provides powerful tools for identification of compounds or cDNAs that modulate the activity of these and other classes of intracellular proteases and their cellular regulators.

## Methods

Additional methodological details are provided as supplemental information (**Supplemental Methods S1**).

### Plasmid Constructions

#### Vectors encoding Caspase or Autophagin cleavable transcription factors

The reporter, Fas-d-S1-TA for Caspase-1 and related proteases (called “S1”), was generated by using PCR and standard recombinant DNA techniques modeled after a previous report [8]. This protein consists of, from N to C termini, amino acids M^1^-L^224^ of a type 1 transmembrane protein, human Fas [39] in which Fas is devoid of the cytosolic death domain (Fas-d), a linker containing the sequence GWEHDG between a XhoI and EagI site, and finally a transcriptional activator (TA) containing the LexA DNA binding domain and the B42 activation domain, taken from plasmids pRS305(Δwbp1-Cub-PLV) [40] and pJG4-5 (Invitrogen) with PCR. Other reporters were made by substituting the linker with oligonucleotides designed to encode in-frame the sequences GWEHGG (“G1”), GDEHDG (“S2”), GDEHGG (“G2”), GDEVDG (“S3”), GDEVGG (“G3”), GTEVDG (“S6”), GTEVGG (“G6”), GLETDG (“S8”), GLETGG (“G8”), GLEHDG (“S9”), GLEHGG (“G9”), or PCR-amplified LC3B gene, after digestion with XhoI and EagI.

#### Plasmids for expressing Caspases and their upstream activators

Plasmids for expression of Caspases in yeast were derived from pRS series vectors [41], [42], [43] and pESC series vectors (Stratagene [Agilent]). Expression levels were adjusted by using constitutive promoters of different strengths (CYC1, ADH, TEF, and GPD) [44] or using the inducible GAL1 promoter, in conjunction with different strength reporter genes carrying variable numbers of *lexA* operators [10], and using plasmids with different replication origins for low or high copy number replication (2µ and CEN/ARS). To further control expression levels, we made several attenuated forms of the promoters by PCR-assisted deletional mutagenesis. Using a standard indicator gene, we determined the approximate relative strength of the promoters to be: ΔCYC4 < ΔCYC2 < CYC1 < ΔGPD2 < ΔGPD1 < ΔADH1 < ADH < ΔTEF3 < ΔTEF2 < ΔTEF1 < TEF. However, the relative locations of the promoters in complex plasmids somewhat affects their strength, especially when two or three genes are contained in one plasmid. In this regard, we sometimes co-expressed two or three genes within one plasmid by placing the genes flanked by the above promoters and inserting transcription termination elements between them, because the selectable marker genes available are limited (*URA3*, *HIS3*, *TRP1*, and *LEU2*). In addition, many of the expressed Caspases and upstream activators were cloned with N-terminal HA or C-terminal HA or FLAG epitope tags for convenience of detection of the protein products by immunoblotting. Examples of complex plasmids are (a) p413-TEF-Fas-d-S8-TA/CYC1-Caspase8-HA, which consists of CEN/ARS origin, HIS3 marker, Fas-d-S8-TA substrate driven by TEF promoter, and a C-terminally HA-tagged pro-Caspase-8 cDNA under control of the CYC1 promoter, as in Figure S20C, and (b) p426-2op-*lacZ*/ΔADH1-FADD, which consists of 2µ origin, URA3 marker, *lacZ* gene under the control of two *lexA* operators, and a FADD gene with expression driven by a short form of the ADH promoter (ΔADH1), as in Figure S21C. Further details about plasmid constructions are provided as Supplemental Information.

#### Construction of expression cDNA libraries

Oligo(dT)-primed or random heptamer-primed cDNA libraries were made in modified p424-GAL1, p424-GAL1-HA, p424-ADH, or p424-ADH-HA plasmids (carrying a TRP1 marker) using mRNAs derived from HEK 293 cells, HepG2 cells, HeLa cells, human liver, or human placenta, as in Supplemental Figure S22A. The mRNAs of HepG2 cells, HeLa cells, human liver, and human placenta were purchased from Ambion. The p424-GAL1 was modified to create 5′XhoI and 3′NotI sites downstream of a GAL1 promoter for insertion of the cDNAs. The p424-GAL1-HA has a HA-tag between the GAL1 promoter and the 5′XhoI site. The p424-ADH and p424-ADH-HA plasmids were modified with 5′XhoI and 3′SfiI sites downstream of the ADH promoter. To construct directional libraries, the first-strand cDNA syntheses were initiated with a NotI-oligo(dT) primer adaptor or a NotI-random hexamer primer adaptor for the p424-GAL1 and p424-GAL1-HA, and with a SfiI-oligo(dT) primer adaptor or a SfiI-random hexamer primer adaptor for the p424-ADH and p424-ADH-HA, according to the manufacturer's instructions (Invitrogen). SalI adapters were ligated to the resultant double-strand cDNAs prior to digestion with NotI or SfiI. The cDNAs with SalI-NotI termini or with SalI-SfiI termini were ligated into the plasmid cloning vectors (XhoI-NotI-cut or XhoI-SfiI-cut, respectively). The number of initial transformants ranged from 1.3×10^6^ to 1.3×10^7^. A human B cell cDNA library was purchased from ATCC. The oligo(dT)-primed cDNAs were inserted into the XhoI cloning site, downstream of a GAL1 promoter. Procedures used for cDNA library screening are provided as Supplemental Information.

#### Reporter gene assays

Plasmids were introduced into yeast by lithium acetate transformation. The yeast strain EGY48, which carries 6 *lexA* operators upstream of *LEU2* gene (6op-*LEU2*), was transformed with pJK103 [10], which carries two *lexA* operators upstream of *lacZ* gene (p426-2op-*lacZ*), and subsequently with reporter plasmids (p413-TEF-Fas-d-S1-TA, p413-TEF-Fas-d-G1-TA, p413-TEF-Fas-d-S2-TA, p413-TEF-Fas-d-S3-TA, p413-TEF-Fas-d-S6-TA, p413-TEF-Fas-d-S8-TA, or p413-TEF-Fas-d-S9-TA). Caspase (all full-length zymogen proforms) expression plasmids (p424-ADH-Caspase1-FLAG, p424-ADH-HA-Caspase2, p424-ADH-Caspase3, p424-TEF-Caspase4, p424-ADH-Caspase5, p424-TEF-HA-Caspase6, p424-ADH-Caspase7, and p424-ADH-HA-Caspase9-FLAG) or empty vector (p424-ADH), were introduced into these backgrounds. As for assays with Caspase-8 and -10, we expressed small amounts of pro-Caspase-8 and -10 with a large amount of FADD by transforming yeast with the dual gene plasmids p424-CYC1-Caspase8-HA/TEF-HA-FADD and p424-CYC1-Caspase10-FLAG/TEF-HA-FADD, because expression of large amounts of human Caspase-8 or Caspase-10 significantly inhibited the cell growth. For ATG4 assays, the yeast strain EGY48 was transformed with ATG4B or catalytic mutant ATG4B(C/A) plasmids (p424-TEF-ATG4B-FLAG or p424-TEF-ATG4B(C/A)-FLAG), reporter plasmid (p413-TEF-Fas-d-LC3B-TA), and pJK103 or pSH18-34 [10], which carries eight *lexA* operators upstream of a *lacZ* gene. The transformants (two independent colonies for each transformation) were streaked on growth plates (minimum synthetic dropout (SD) medium containing 2% glucose and 50 µg/ml leucine) or on selection plates (SD medium containing 1% galactose, 0.2% raffinose, BU salts, and 80 µg/ml X-gal). Yeast growth and blue color development were monitored for four to six days at 30°C.

#### Reporter gene assays in liquid media using 384-well plates

For caspase assays, the assays were performed in a total volume of 40 µl in triplicate. First, 20 µl of liquid selection media containing X-gal and a series of concentrations of reagents such as z-VAD was dispensed into each well of 384-well plates. Next, confluent yeast cells expressing various Caspases and cleavable substrates were collected, washed with water, and suspended in selection media of the same volume as the culture media. The yeast suspension was diluted to 1∶5-10 (v:v) with the selection media, and 20 µl aliquots were added to the 384-well plates. For ATG4B assays, yeast cells expressing ATG4B and LC3 fused-transcription factor were cultured overnight in standard growth medium, then the yeast cells were diluted in selection medium (SD medium containing 1% galactose, 0.2% raffinose, BU salts, and 100 µg/ml X-gal) at 10^4^/ml and dispensed 100 µl into each well of 384-well plates. Absorbance at 620 nm was measured 2–3 days after culture at 30°C.

#### HTS Assays

For the Fas/FADD/Caspase-8 and the NLRP1/Asc/Caspase-1 assays, EGY48 yeast containing the desired plasmids were streaked onto SD plates (6.8 g Yeast Nitrogen Base w/o amino acids, 20 mg arginine, 50 mg threonine, 30 mg isoleucine, 60 mg phenylalanine, 20 mg valine) containing agar (1.7%), supplemented with 2% α-D-glucose and 50 µg/mL leucine. The plates were incubated at 30°C for 48 hrs and a colony was picked and transferred to a 14 ml polypropylene tube containing 2 ml of SD media broth supplemented with α-D-glucose and leucine as above and grown at 30°C for 16–24 hrs with shaking. Then, 1 ml of the overnight culture was transferred into 20 mls of growth media in a 500 ml flask and shaken at 30°C for 16–24 hrs. The cells were collected by centrifugation at *1000xg* for 5 minutes at room temperature, and washed with 20 mls of sterile water, then resuspended in 20 mls of SD broth supplemented with 1% galactose and 0.2% raffinose. The HTS Assay was performed at a final compound concentration of 10 µM (1% DMSO), with cell densities of 2×10^5^ cells/ml (2×10^5^ cells) in a volume of 40 µl. The assay was assembled by the addition of 4 µl of ≈100 µM compounds (final concentration of 10 µM) in 10% DMSO (1% final DMSO) to clear polystyrene 384-well microplates using a Beckman-Coulter Biomek FX, then 18 µl of cell suspension (1.1×10^7^ cells/ml) (2×10^5^ cells/well) and 18 µl of X-gal suspended in Selection Media (for a final concentration of 400 µg/mL) were added to the wells using a Matrix WellMate bulk reagents dispenser. Remaining solutions were added by using the WellMate from Matrix Technologies Corp. Controls were included with each plate, corresponding to cells treated with DMSO (without compounds) and cells cultured with and without X-gal. The plates were sealed with breathable sealing film (from Axygen Scientific) to reduce evaporation and transferred to 30°C incubators. After 48 hours, the plates were brought to room temperature, the breathable film removed and replaced with the transparent polyester tape seal, and the plates were mixed, pelleted briefly and read using a Beckman DTX 880, recording absorbance at 620 nm.

#### Yeast-based counter-screen assays

EGY48 yeast inducibly (*GAL1* promoter) expressing the LexA/B42 transcription factor or containing the empty vector were used to detect compounds that directly inhibit the *lacZ* reporter gene or that alter β-galactosidase activity. The plasmids employed were p424-GAL1 and p424-GAL1-TA (transcriptional activator) with transformed cells selected on tryptophan-deficient plates. Yeast expressing Caspase-1 activated by expression of high levels of Asc (ΔTEF3-Caspase-1-FLAG/TEF-HA-Asc) were used to detect compounds that cross-react with hits from the Fas/FADD/Caspase-8 screen. The Asc/Caspase-1 yeast cells were cultured and assayed under identical conditions to those for the primary NLRP1 HTS assay (see above), using cells expressing 6op-*LEU2*/2op-*lacZ* reporter genes and TEF-Fas-d-S1-TA substrate.

## Supporting Information

Supplemental Methods S1 (0.03 MB PDF)

Table S1 Determination of IC50 values for zVAD-fmk inhibition of Caspases in yeast. Yeast expressing various Caspases alone (at high levels) or in combination with upstream activators (at low levels) and cleavable substrates containing appropriate tetrapeptides reorganized by these proteases were used in 384 well β-galactosidase activity assays to assess inhibition by zVAD-fmk. The compound was titrated into assays at various concentrations and percentage inhibition was determined. IC50 values were determined, using PRIZM software for analysis.
(0.01 MB PDF)

Figure S1 Substrate sequence- and Caspase activity-dependent cleavage of the S1, S2, and S9. Yeast transformants were plated on leucine-deficient medium containing X-gal. (A) The recipient yeast cell strains, EGY48-6op-LEU2/2op-lacZ/TEF-Fas-d-S1(WEHD)-TA (S1), or EGY48-6op-LEU2/2op-lacZ/TEF-Fas-d-G1(WEHG)-TA (G1) were transformed with the plasmids encoding the active wild type Caspase1 (WT), the catalytically-defective Caspase1 (C285→G285), and the empty vector (-). (B) The recipient yeast cell strains, EGY48-6op-LEU2/2op-lacZ/TEF-Fas-d-S2(DEHD)-TA (S2), or EGY48-6op-LEU2/2op-lacZ/TEF-Fas-d-G2(DEHG)-TA (G2) were transformed with the plasmids encoding the active wild type Caspase2 (WT), the catalytically-defective Caspase2 (C320→A320), and the empty vector (-). (C) The recipient yeast cell strain, EGY48-6op-LEU2/2op-lacZ/TEF-Fas-d-S9(LEHD)-TA (S9), or EGY48-6op-LEU2/2op-lacZ/TEF-Fas-d-G9(LEHG)-TA (G9), were transformed with the plasmids encoding the active wild type Caspase9 (WT), the catalytically-defective Caspase9 (C287→A287), and the empty vector (-).
(0.05 MB PDF)

Figure S2 Flow chart for cDNA library screening using a reporter gene strategy based on cleavable transcription factor. The example here is S1 substrate. The yeast strain EGY48 containing 6op-LEU2 and 2op-lacZ reporter genes and Caspase-cleavable substrate (expressed from TEF-Fas-d-S1-TA) was transformed (left) with a HEK293 cell cDNA library (oligo-dT-primed, PGAL1 promoter). Independent colonies of 5.1×105 clones appeared on growth plates in 48 hours. Cells were collected, pooled, and a portion (3.6×106) was seeded on leucine-deficient selection plates containing X-gal. Blue-colored colonies (n = 42) appeared within a week, of which 6 corresponded to clone #ZB311 encoding full-length Caspase-4 (which is known to cleave WEHD tetrapeptide [Thornberry, N.A, et. al., J. Biol. Chem. 272, 17907–17911 (1997)]), while the rest were false positives. (Right) Another cDNA library screen (HEK293 random-primed, ADH promoter used to drive expression) resulted in 2×106 clones, which were pooled and 2.1×107 cells were screened on leucine-deficient, X-gal-containing plates, resulting in 10 blue colonies. Five clones (including #XE501) encoding a fragment of Casapse-1 (L89-G403) were isolated. The remaining five clones were apparent false positives.
(0.04 MB PDF)

Figure S3 Flow chart for cDNA library screening using reporter gene strategy based on cleavable transcription factor. The example here is S3/S7 substrate. Yeast strain EGY48 containing 6op-LEU2 and 2op-lacZ reporter genes and the DEVD-containing cleavable transcription factor (expressed from TEF-Fas-d-S3-TA) was transformed with a HEK293 cell cDNA library (random-primed, PGAL1 promoter, HA-tagged) (left). Independent colonies (1.0×106) appeared on growth plates within 48 hours. Cells were collected, pooled, and a portion (5.0×106) was seeded on the leucine-deficient selection plates containing X-gal. Blue-colored colonies (n = 21) appeared within a week, of which 3 clones (including #ZB331) encoded full-length Caspase-3, while the rest were false positives. In a similar experiment (right), another HEK293 cDNA library (oligo-dT-primed, PADH promoter) (1.8×106 initial colonies) was screened, resulting in 9 positive clones (including #X8312) encoding full-length Caspase-7. The remaining five clones were false positives. Both cloned Caspases are known to cleave DEVD tetrapeptide [Thornberry, N.A, et. al., J. Biol. Chem. 272, 17907–17911 (1997)].
(0.04 MB PDF)

Figure S4 Use of one-component yeast-based Caspase activity assay for cDNA library screening. (A) The plasmids containing library cDNAs from Figure S2 were recovered and re-transformed into yeast cells containing 6op-LEU2 and 2op-lacZ reporter genes with either cleavable (WEHD-containing) (expressed from TEF-Fas-d-S1-TA) or non-cleavable (WEHG-containing) transcription factor substrate (expressed from TEF-Fas-d-G1-TA) to confirm whether they cleave S1 specifically. As controls, yeast were transformed with a plasmid encoding Caspase-1 (“Positive” control) or the empty vector (“Negative” control). Assays were performed in duplicate, with cells grown on selection plates for 4 days. Note that the cDNA library clones supported lacZ reporter gene activation only when co-expressed with the S1 cleavable substrate. (B) The cDNA library plasmids from Figure S3 were recovered and used to re-transform the yeast strains containing either the same cleavable DEVD-containing or DEVG non-cleavable transcription factor, expressed from TEF-Fas-d-S3-TA (“S3” substrate) and TEF-Fas-d-G3-TA (“G3” substrate), respectively. Assays were performed in duplicate and cells grown on selection plates for 4 days. Note that the two cDNA library clones activated the lacZ reporter gene only when co-expressed with cleavable S3 (DEVD-containing) substrate. As controls, yeast cells were transformed with plasmids encoding Caspase-3 (“Positive” control) or the empty vector (“Negative” control).
(0.04 MB PDF)

Figure S5 Validation of yeast-based assays for effector Caspase activators - two- and three-component systems. Yeast EGY191 strain containing 2op-LEU2/2op-lacZ or EGY48 strain containing 6op-LEU2/2op-lacZ were employed for developing assays for activators of downstream effector Caspases (e.g. Caspases-3 and -7). Yeast transformants were plated on leucine-deficient medium containing X-gal. Yeast were transformed with plasmids encoding membrane tethered transcription factor substrate with either DEVD-containing cleavable (S3) or DEVG-containing non-cleavable (G3) linkers, and with plasmids encoding pro-Caspase-3 (C3) or pro-Caspase-7 (C7) or the corresponding empty vector (-). (Note that the optimal tetrapeptide sequence for both Caspase-3 and Caspase-7 has previously been reported to be DEVD). Yeast transformants were as follows: (A) S3 Substrate/No Caspase: EGY191-2op-LEU2/2op-lacZ/ΔTEF3-Fas-d-S3 (DEVD)-TA; S3 Substrate/Caspase-3: EGY191-2op-LEU2/2op-lacZ/ΔTEF3-Fas-d-S3/7(DEVD)-TA/ΔCYC2-HA-Caspase3; G3 Substrate/Caspase-3: EGY191-2op-LEU2/2op-lacZ/ΔTEF3-Fas-d-G3/7(DEVG)-TA/ΔCYC2-Caspase-3; and (B) S3 Substrate/No Caspase: EGY48-6op-LEU2/2op-lacZ/TEF-Fas-d-S3(DEVD)-TA; S3 Substrate/Caspase-7: EGY48-6op-LEU2/2op-lacZ/TEF-Fas-d-S3(DEVD)-TA/CYC1-Caspase-7; G3 Substrate/Caspase-7: EGY48-6op-LEU2/2op-lacZ/TEF-Fas-d-G3(DEVG)-TA/CYC1-Caspase-7. These yeast transformants were then subsequently transformed with plasmids expressing a small amount of Caspase-9 (driven from the CYC promoter) or plasmids expressing large amounts of wild-type (WT) or catalytically-defective (C287→A287) Caspase-9 (driven from the TEF promoter), with or without an active form (gain of function mutant not requiring cytochrome c for activation) of Apaf-1 (driven by TEF-promoter) or the corresponding empty vectors (-) (Haraguchi M, Torii S, Matsuzawa S, et al. J Exp Med 2000;191:1709-20). Note that the large amount of Caspase-9 (expressed from TEF promoter) activated the cleavable S3(DEVD) substrate when co-expressed with pro-Caspase-3 or pro-Caspase-7, but not in their absence, thus constituting a 2-component system. No lacZ reporter gene activity was detected when the non-cleavable substrate (DEVG) was employed (G3). In contrast, expressing a small amount of Caspase-9 (from the CYC promoter) or the catalytically-defective Caspase9 (C287→A287) did not activate Caspase-3 or Caspase-7. However, co-expressing active Apaf-1* with a small amount of pro-Caspase-9 activated the lacZ reporter gene in yeast expressing pro-Caspase-3 or -7 (but not in the absence of these downstream effector Caspases), thus constituting a 3-component system.
(0.06 MB PDF)

Figure S6 Validation of yeast-based assays for initiator Caspase activators: two-component systems. Yeast EGY48 strain containing 6op-LEU2/2op-lacZ was employed for developing 2-component assays for activators of upstream initiator Caspases (e.g. Caspases-1, 2, 8, 9, 10). Two independent clones of yeast transformants were plated on leucine-deficient medium containing X-gal. Yeast were transformed with plasmids encoding various membrane tethered transcription factor substrates containing (A) WEHD (“S1”), (B) DEHD (“S2”), (C) LEHD (“S9”) or (D) LETD (“S8”) cleavable linkers or their corresponding non-cleavable glycine mutants (“G1”, “G2”, “G9”, “G8”). The yeast were also transformed with plasmids encoding wild-type (WT) or catalytically inactive mutants of proforms various initiator Caspases expressed from relatively weak promoters (e.g., CYC1; ΔTEF3), including (A) pro-Caspase-1, (B) pro-Caspase-2, (C) pro-Caspase-9, and (D) pro-Caspase-10. (Note that the optimal betrapeptide sequences are the same for Caspase-8 and 10). These yeast were transformed with empty vectors (-) or plasmids encoding upstream activators of the Caspases, including (A) Asc, (B) RAIDD, (C) Apaf-1*, and (D) FADD, expressed from strong promoters (either GPD or TEF). Note that the lacZ reporter gene was activated only when the combination of an initiator Caspase and upstream activator was co-expressed, along with a cleavable substrate. Transformants: The transformed yeast cell clones are: (A) EGY48-6op-LEU2/2op-lacZ/TEF-Fas-d-S1(WEHD)-TA/ΔTEF3-Caspase1-FLAG (S1,C1(WT)), EGY48-6op-LEU2/2op-lacZ/TEF-Fas-d-G1(WEHG)-TA/ΔTEF3-Caspase1-FLAG (G1,C1(WT)), or EGY48-6op-LEU2/2op-lacZ/TEF-Fas-d-S1(WEHD)-TA/ΔTEF3-Caspase1(C285→G285)-FLAG, (S1,C1(C285→G285)), were transformed with the plasmids encoding the activator Asc, or the empty vector (-); (B) EGY48-6op-LEU2/2op-lacZ/ΔTEF2-Fas-d-S2(DEHD)-TA/ΔGPD1-HA-Caspase2- FLAG(S2,C2(WT)), EGY48-6op-LEU2/2op-lacZ/ΔTEF1-Fas-d- G2(DEHG)-TA/ΔGPD1-HA-Caspase2-FLAG (G2,C2(WT)), or EGY48-6op-LEU2/2op-lacZ/ΔTEF2-Fas-d-S2(DEHD)-TA/ΔGPD1-HA-Caspase2(C320→A320)-FLAG (S2,C2(C320→A320)), were transformed with plasmids encoding the activator RAIDD, or the empty vector (-); (C) EGY48-6op-LEU2/2op-lacZ/TEF-Fas-d-S9(LEHD)-TA/TEF-HA-Caspase9 (S9,C9(WT)), EGY48-6op-LEU2/2op-lacZ/TEF-Fas-d-G9(LEHG)-TA/TEF-HA-Caspase9 (G9,C9(WT)), or EGY48-6op-LEU2/2op-lacZ/TEF-Fas-d-S9(LEHD)-TA/TEF-HA-Caspase9(C287→A287) (S9,C9(C287→A287)), were transformed with plasmids encoding an active form of Apaf-1 (Apaf*), or the empty vector (-); (D) EGY48-6op-LEU2/2op-lacZ/GPD-Fas-d-S8(LETD)-TA/ADH-Caspase10-FLAG (S8,C10(WT)), EGY48-6op-LEU2/2op-lacZ/GPD-Fas-d-G8(LETG)-TA/ADH-Caspase10-FLAG (G8,C10(WT)), or EGY48-6op-LEU2/2op-lacZ/GPD-Fas-d-S8(LETD)-TA/ADH-Caspase10(C358→A358)-FLAG (C10(C358→A358)), were transformed with the plasmids encoding the activator FADD, or the empty vector (-). The large amount of FADD is enough to activate Caspases-10 by itself.
(0.06 MB PDF)

Figure S7 Specificity of upstream activators of initiator Caspases - tested by 2-component systems. Yeast transformants were prepared and tested as described in Figures S5 and S6, except a matrix of plasmid combinations was prepared to evaluate the specificity of upstream activators. The substrates and Caspases tested are indicated across the top, while the activators are indicated along the side. Note that results obtained were as predicted, with (1) Apaf-1* activating pro-Caspase-9, but not other initiator Caspases; (2) RAIDD activating pro-Caspase-2, but not other Caspases; (3) FADD activating pro-Caspases-8 and 10, but not other Caspases, and (4) Asc activating pro-Caspase-1 and 8. Note that while Asc contains a CARD that pairs with a complementary CARD in pro-Caspase-1 and would not be necessarily predicted to activate the DED-containing protease Caspase-8, it has previously been reported that Asc is an activator of Caspase-8 (Hasegawa M. et.al. J Biol Chem 280:15122-30 (2005); Masumoto, J. et. al., Biochem. Biophys. Res. Commun 303: 69-73 (2003). Transformants: Transformed yeast clones were as follows: S1,C1:EGY48-6op-LEU2/2op-lacZ/TEF-Fas-d-S1(WEHD)-TA/ΔTEF3-Caspase1-FLAG; S2,C2:EGY48-6op-LEU2/2op-lacZ/ΔTEF2-Fas-d-S2(DEHD)-TA/ΔGPD1-HA-Caspase2-FLAG; S8,C8:EGY48-6op-LEU2/2op-lacZ/GPD-Fas-d-S8/10(LETD)-TA/CYC1-Caspase8-HA; S9,C9:EGY48-6op-LEU2/2op-lacZ/TEF-Fas-d-S9(LEHD)-TA/TEF-HA-Caspase-9; S8,C10:EGY48-6op-LEU2/2op-lacZ/GPD-Fas-d-S8/10(LETD)-TA/ADH-Caspase10- FLAG. These cells were transformed with the plasmids encoding the activators (Asc, RAIDD, FADD, and Apaf*). For controls (-), the “empty” version of the plasmids were introduced.
(0.05 MB PDF)

Figure S8 Validation of 3-component yeast-based Caspase assay reconstituting DISC. Yeast transformants were prepared to assess the performance of the Fas/FADD/Caspase-8/10 three-component systems. Two independent clones of each transformant were plated on leucine-deficient medium containing X-gal. Substrates included LETD-containing cleavable (S8) and LETG-containing non-cleavable (G8) transcription factors, while Caspase expression plasmids included WT pro-Caspases-8 (A) and -10 or a catalytic mutant of Caspase-10. (B) FADD was expressed at low levels from a ΔADH1 promoter. Note that the lacZ reporter gene was activated only when the combination of Fas, FADD, and either WT pro-Caspase-8 or -10 was co-expressed and only when a cleavable substrate (S8) was employed. (Note that the optimal tetrapeptide cleavage sequence is the same for Caspases-8 and -10). Transformants: (A) Yeast transformants included EGY48-6op-LEU2/2op-lacZ/TEF-Fas-d-S8 (LETD)-TA/CYC1-Caspase-8-HA (S8,C8) EGY48-6op-LEU2/2op-lacZ/TEF-Fas-d-G8(LETG)-TA/CYC1-Caspase8-HA (G8,C8), without (-) or with Fas, and without (-) or with FADD, which were expressed from either ADH and ΔADH1 promoter, respectively, to achieve high expression of Fas and low expression of FADD. (B) Yeast transformants included: EGY48-6op-LEU2/2op-lacZ/GPD-Fas-d-S8(LETD)-TA/CYC1-Caspase10-FLAG (S8,C10); EGY48-6op-LEU2/2op-lacZ/GPD-Fas-d-G8(LETG)-TA/CYC1-Caspase10-FLAG (G8,C10); and EGY48-6op-LEU2/2op-lacZ/GPD-Fas-d-S8 (LETD)-TA/CYC1-Caspase10(C358→A358)-FLAG (S8,C10(C358→A358)) each without (-) or with Fas, and without (-) or with FADD-expressing vector or the corresponding empty vectors.
(0.05 MB PDF)

Figure S9 Flow chart for cDNA library screening using 3 component system - application to death receptor cloning-Example #1. The screening strategy is essentially the same as outlined in Figure 3. The yeast transformant EGY48-6op-LEU2/2op-lacZ/TEF-Fas-d-S8(LETD)-TA/CYC1-Caspase8-HA/ΔADH1-FADD was transformed with a HepG2 cell cDNA library (oligo dT-primed, PADH promoter). Independent colonies (1.7×106) appeared on growth plates within 48 hours. Cells were collected, pooled, and a portion (2.4×107) was seeded on the leucine-deficient selection plates containing X-gal. Blue-colored colonies (n = 150) appeared within a week, 13 of which encoded DR4, (#XA514), and 15 clones encoded DR5 variant 2 (#ZA512), while the rest were false positives. Screening another cDNA library from HepG2 cells (random-primed, PADH promoter) yielded eight DR5 (#XD108) clones.
(0.05 MB PDF)

Figure S10 Flow chart for cDNA library screening using 3 component system to clone death receptors - Example #2. The screening strategy is essentially the same as outlined in Figure 3. The yeast transformant EGY48-6op-LEU2/2op-lacZ/TEF-Fas-d-S8(LETD)-TA/CYC1-Caspase8-HA/ΔADH1-FADD was transformed with a human liver cDNA library (random-primed, PADH promoter) and a HeLa cell cDNA library (random-primed, PADH promoter).
(0.04 MB PDF)

Figure S11 Flow chart for cDNA library screening using 3-component system to to clone death receptors - Example #3. The screening strategy is essentially the same as outlined in Figure 3. The yeast transformant EGY48-6op-LEU2/2op-lacZ/TEF-Fas-d-S8(LETD)-TA/CYC1-Caspase8-HA/ΔADH1-FADD was transformed with a HEK293T cell cDNA library (oligo-dT-primed, PADH promoter) and a HEK293T cell cDNA library (random-primed, PADH promoter, HA-tagged).
(0.04 MB PDF)

Figure S12 Schematic representation of 3-component system used for cloning adapter protein that links Fas to pro-Caspase-10. (A) The zymogen pro-Caspase-10 was expressed (from CYC1-promoter) with substrate containing the LETD-containing cleavage element S8 (from plasmid p413-GPD-Fas-d-S8-TA/CYC1-Caspase-10-FLAG) in the yeast EGY48 expressing 6op-LEU2. Fas was also expressed without activating the Caspase-10 from plasmid p426-2op-lacZ/ADH-Fas. (B) Addition of FADD activates Caspase-10, releasing the transcription factor.
(0.04 MB PDF)

Figure S13 Validation of adapter protein cloning system for Fas/FADD/Caspase-8/10: Reconstituted DISC. Yeast cell transformants were prepared to assess the performance of the Fas/FADD/Caspase-8 or Fas/FADD/Caspase-10 three-component systems. Two independent clones of each transformant were plated on leucine-deficient medium containing X-gal and grown for 4 days. Substrates included LETD-containing cleavable (S8) and LETG-containing non-cleavable (G8) transcription factors, while Caspase expression plasmids included wild-type (WT) pro-Caspases-8 (A) and -10 or a catalytic mutant of Caspase-10 (C358→A358) (B). Note that the lacZ reporter gene was activated only when the combination of Fas, FADD, and either WT pro-Caspase-8 or -10 was co-expressed and only when a cleavable substrate (S8) was employed. Transformants: (A) Yeast cell transformants included: EGY48-6op-LEU2/2op-lacZ/TEF-Fas-d-S8 (LETD)-TA/CYC1-Caspase8-HA (S8,C8) and EGY48-6op-LEU2/2op-lacZ/TEF-Fas-d-G8 (LETG)-TA/CYC1-Caspase8-HA (G8,C8), with empty vector (-) or with plasmids encoding FADD (expressed from CYC1 promoter) or Fas (expressed from ADH promoter). (B) Yeast cell transformants included EGY48-6op-LEU2/2op-lacZ/GPD-Fas-d-S8(LETD)-TA/CYC1-Caspase10-FLAG (S8,C10), EGY48-6op-LEU2/2op-lacZ/GPD-Fas-d-G8 (LETG)-TA/CYC1-Caspase10-FLAG (G8,C10), and EGY48-6op-LEU2/2op-lacZ/GPD-Fas-d-S8(LETD)-TA/CYC1-Caspase10(C358→A358)-FLAG (S8,C10(C358→A358)), each with empty vector (-) or with plasmids encoding FADD or Fas as above.
(0.05 MB PDF)

Figure S14 Flow chart for cDNA library screening using 3 component system to clone adapters. Yeast transformant EGY48-6op-LEU2/2op-lacZ/GPD-Fas-d-S8(LETD)-TA/ADH-Fas/CYC1-Caspase10-FLAG was transformed with a HEK293 cell cDNA library (random-primed, PADH promoter, HA-tagged). Independent colonies (∼2.2×106) appeared on growth plates within 48 hours. Cells were collected, pooled, and a portion (3.2×107) was seeded on leucine-deficient selection plates containing X-gal. Blue-colored colonies (n = 200) appeared within a week, five of which encoded full-length Caspase-2 (#XA227), five encoded a fragment of Caspase-2 (#ZA214), five encoded a fragment of Caspase-8 (#XA221), and 24 encoded full-length FADD (#XA212), while the rest were apparent false positives.
(0.03 MB PDF)

Figure S15 Examples of cDNA cloning results. The clones that activated the reporter genes were characterized by recovery of cDNA library plasmids and retransformation into yeast expressing Fas and pro-Caspase-10 (A), pro-Caspase-10 without Fas (B), or neither (C). Among the positive clones were a full-length Caspase-2 (#XA227), a fragment of Caspase-2 (#ZA214, V130-L312), a fragment of Caspase-8 (#XA221, M1-K438), and full-length FADD (#XA212). Assays were performed in duplicate, growing cells for 4 days on plates.
(0.05 MB PDF)

Figure S16 Optimization of signal:noise ratio in microtiter plates: cell density. EGY48 cells containing the 3-component Fas/FADD/Caspase-8 system and Caspase-8-cleavable reporter were seeded at 2.35×104/well (A), 2×105/well (B), or 3.76×105/well (C) in 384 well plates to compare cell densities. Cells were cultured at 30°C. The activity of β-galactosidase was measured after 72 hrs at various times after initiating cultures (mean+std dev; n = 3), for cells grown without (white circles) or with (black circles and black squares) X-gal and grown in the absence (circles) or presence (black squares) of 100 µM zVAD-fmk Caspase inhibitor.
(0.04 MB PDF)

Figure S17 Optimization of signal:noise ratio in microtiter plates: time and X-gal concentration. (A) EGY48 cells containing the 3-component Fas/FADD/Caspase-8 system and Caspase-8-cleavable reporter were grown at 2×105 cells/well in 384 well plates, comparing β-galactosidase activity at various times (mean ± std dev; n = 3), for cells grown in the absence (black squares) or presence (circles) of X-gal and in the absence (circles) or presence (squares) of 100 µM zVAD-fmk Caspase inhibitor. (B) EGY48 cells containing the 3-component NLRP1ΔLRR/Asc/Caspase-1 system and Caspase-1-cleavable reporter were grown at 2×105 cells/well in 384 well plates, comparing X-gal concentrations. The activity of β-galactosidase was measured after 72 hrs culture (mean ± std dev; n = 3) for cells grown in the absence (red circles) or presence (all others) X-gal. Various concentrations of X-gal were compared.
(0.04 MB PDF)

Figure S18 Determination of Z' scores for NLRP1(NALP1) and Fas(CD95) 3-component yeast-based HTS assays using caspase inhibitor zVAD-fmk. (A) Yeast strain EGY48 harboring the NLRP1/ASC/Caspase-1 proteolytic network (B) or the Fas/FADD/Caspase-8 proteolytic network were cultured at 2×105 cells/well for 48 hours in SD media containing 400 µg/ml X-gal with DMSO solvent control (blue) or zVAD in DMSO (100 µM final concentration) (purple). The Z' scores were determined by comparison of the DMSO-treated samples with the zVAD-treated samples.
(0.34 MB PDF)

Figure S19 Experiments with ATG4B-expressing yeast. (A) Determination of cell density dependence. The production of β-galactosidase activity (y-axis) in 384 well plate format was compared for various densities of EGY48 cells (x-axis) harboring plasmids encoding ATG4B and the LC3-containing substrate and lacZ reporter genes containing either 2 (red, orange) or 8 (blue, green) LexA-operators. Medium consisted of 1% galactose/2%raffinos, with 100 µg/ml X-gal. Cells were cultured at 30°C for 48 hrs, then absorbance was measured at OD 620 nm. Data represent mean±std dev (n = 4). (B) The time-course of β-galactosidase activity generation was compared for EGY48 cells containing plasmids encoding LEU2 and lacZ reporter with (2 vs 8) LexA operators. Cells were plated and culture as described above. Data represent mean ± std dev (n = 4).
(0.02 MB PDF)

Figure S20 Plasmids for co-expression of Caspases and substrate cleavable transcription factors in yeast. The plasmid p413 was used as the backbone for these constructions, containing CEN/ARS centromeric origin for low-copy episomal replication in yeast (S. cerevisiae) and HIS3 gene for selection in his3 yeast strains. (A) The plasmid p413-TEF-Fas-d-S1-TA, where expression of the Fas-LexA/B42 membrane tethered transcription factor with WEHD linker (Caspase-1/4/5 cleavable) is driven by the TEF promoter. (B) The plasmid p413-TEF-Fas-d-S1-TA/ΔTEF3-Caspase-1-FLAG, contains two additional transcriptional units, where expression of the Fas-LexA/B42 membrane tethered transcription factor with WEHD linker (Caspase-1/4/5 cleavable) is driven by the TEF promoter and expression of pro-Caspase-1 with C-terminal FLAG tag is driven by an attenuated TEF3 promoter (ΔTEF3). (C) The plasmid p413-TEF-Fas-d-S8-TA/CYC1-Caspase-8-HA contains two additional transcriptional units, where expression of the Fas-LexA/B42 membrane tethered transcription factor with LETD linker (Caspase-8/10 cleavable) is driven by the TEF promoter and expression of pro-Caspase-8 with C-terminal HA tag is driven by CYC1 promoter. (D) The plasmid p413-GDP-Fas-dS8-TA/CYC1-Caspase-10-FLAG, similarly contains two additional transcriptional units, where expression of the same Caspase-8/10 cleavable Fas-LexA/B42 substrate as above is driven by GDP promoter and where expression of pro-Caspase-8 with C-terminal FLAG tag is driven by CYC1 promoter. Transcriptional termination elements from the CYC1 and ADH genes were employed as illustrated.
(0.07 MB PDF)

Figure S21 Plasmids for expression of upstream activators of Caspases and lacZ reporter gene in yeast. The plasmid p426 was used as the backbone for these constructions, containing 2µ plasmid origin for high-copy episomal replication in yeast and URA3 gene for selection in ura3 yeast strains. (A) The plasmid p426-2op-lacZ contains a lacZ gene driven by a minimal promoter containing two tandem copies of binding sites for the LexA transcription factor, followed by ADH gene termination element. (B) Plasmid p426-2op-lacZ/CYC1-HA-Asc contains an additional transcriptional unit, where expression of human cDNA encoding N-terminal HA-tagged Asc protein is driven by a CYC1 promoter, and followed by ADH gene termination element. (C) In plasmid p426-2op-lacZ/ΔADH1-FADD, expression of a human FADD cDNA is driven by ΔADH1 promoter and followed by ADH termination element. (D) Plasmid p426-2op-lacZ/ADH-Fas contains a human Fas(CD95) cDNA driven by ADH promoter, and followed by CYC1 gene termination element.
(0.05 MB PDF)

Figure S22 Yeast expression plasmids for functional screening of cDNA libraries and expression of upstream activators of Caspases. The plasmid p424 was used as the backbone for these constructions, containing 2µ plasmid origin for high-copy episomal replication in yeast (S. cerevisiae) and TRP1 gene for selection in trp1 yeast strains. (A) The plasmid p426-GAL1-cDNAs contains human cDNAs directionally cloned downstream of a GAL1 promoter, followed by CYC1 gene termination element. (B) Plasmid p424-CYC1-HA-Asc contains a human cDNA encoding N-terminal HA-tagged Asc protein cloned downstream of a CYC1 promoter and followed by CYC1 gene termination element. (C) In plasmid p424-CYC1-HA-Asc/TEF-NLRP1ΔLRR, an additional transcriptional unit was added to p424-CYC1-HA-Asc above, where expression of a human cDNA encoding a gain-of-function NLRP1 mutant lacking LRRs (Faustin, B, et al. Molecular Cell 25;713, 2007) is driven by a TEF promoter and followed by ADH gene termination element.
(0.04 MB PDF)
