# Supplementary material for: Versatile Assays for High Throughput Screening for Activators or Inhibitors of Intracellular Proteases and Their Cellular Regulators
Source: PLoS One. 2009 Oct 30;4(10):e7655. doi: 10.1371/journal.pone.0007655 (PMC2764853; doi:10.1371/journal.pone.0007655)
Supplement: Supplemental Methods S1 — (0.03 MB PDF) [file pone.0007655.s001.pdf]

SUPPLEMENTAL INFORMATION

I. METHODS

Plasmids constructions. Details about construction of plasmids are available upon request. Diagrams of complex plasmids containing 3 or more genes are provided as Supplemental data. The cDNAs encoding human Caspases, Adapter proteins, Death Receptors, and NLRs, in wild-type and mutant versions have been described previously J-M. Bruey, et al., *Nature Cell Biology* 2, 645 (2000).M. Krajewska, et al., *Exp Neurol* 189 (2), 261 (2004). H. Marusawa, et al., *EMBO J* 22, 2729 (2003).T. Miyazaki, et al., *Nature Immunol* 3, 4 (2002). A.D. Schimmer, et al., *Cancer Cell* 5, 25 (2004).

Cloning systems for Caspase activators. To clone cDNAs encoding proteins that activate Caspase-1, the yeast strain EGY48 containing 6op-*LEU2*/2op-*lacZ* was transformed with plasmids containing transcriptional units of TEF-Fas-d-S1-TA (substrate) and ΔTEF3-Caspase1-FLAG (pro-Caspase-1), as illustrated in Supplemental Figure S20B, and selected on histiodine-deficient plates. As controls, cells were subsequently transformed with the Asc-expressing plasmid p424-TEF-HA-Asc or empty vector and selected on tryptophan-deficient plates.

To clone cDNAs encoding proteins that activate Caspase-2, the yeast strain EGY48 containing 6op-*LEU2*/2op-*lacZ* was transformed with plasmids containing transcriptional units of ΔTEF2-Fs-d-S2-TA (substrate) and ΔGPD1-HA-Caspase2-FLAG (pro-Caspase-2) and selected on histiodine-deficient plates. As controls, cells were subsequently transformed with the RAIDD-expressing plasmid p424-TEF-HA-RAIDD or empty vector and selected an tryptophan-deficient plates.

To clone cDNAs encoding proteins that activate Caspase-3, the yeast strain EGY191 containing 2op-*LEU2*/2op-*lacZ* was transformed with plasmids containing transcriptional units for ΔTEF3-Fas-d-S3-TA and ΔCYC2-Caspase-3 and selected on histiodine-deficient plates. As controls, cells were transformed with the Caspase-9-expressing plasmid p424-ADH-HA-Caspase9-FLAG or empty vector and selected on tryptophan-deficient plates.

To clone cDNAs that activate Caspase-7, the yeast strain EGY48 expressing 6op-*LEU2*/2op-*lacZ*/TEF-Fas-d-S3-TA/CYC1-Caspase-7 was created. The small amount of Caspase-7 exists as inactive zymogen, and does not cut the S3 site. As a positive control, pro-Caspase-7 was activated by expressing Caspase-9 at high levels (p424-ADH-HA-Caspase9-FLAG). Active Caspase-9 did not cut the S3 site.

To clone cDNAs encoding proteins that activate Caspase-8, the yeast strain EGY48 containing 6op-*LEU2*/2op-*lacZ* was transformed with plasmids containing transcriptional units for GPD-Fas-d-S8-TA and CYC1-Caspase8-HA and selected on histiodine-deficient plates. As controls, cells were transformed with FADD-expression plasmid p424-TEF-HA-FADD or empty vector and selected on tryptophan-deficient plates.

To clone cDNAs that activate Caspase-9, the yeast strain EGY48 containing 6op-*LEU2*/2op-*lacZ* was transformed with plasmids containing transcriptional units for TEF-Fas-d-S9-TA and TEF-HA-Caspase-9, on a low-copy plasmid p413 (CEN/ARS). The small amount of pro-Caspase-9 exists as inactive zymogen, and does not cut the S9 site. As controls, cells were transformed with the Apaf-1*- expressing plasmid p424-TEF-HA-Apaf* or empty vector and selected on tryptophan-deficient plates.

To clone cDNAs encoding proteins that activate Caspase-10, the yeast strain EGY48 containing 6op-*LEU2*/2op-*lacZ* was transformed with plasmids containing transcriptional units for GPD-Fas-d-S8-TA and ADH-Caspase10-FLAG and selected on histiodine-deficient plates. As controls, cells were transformed with the FADD-expressing plasmid p424-TEF-HA-FADD or empty vector and selected on tryptophan-deficient plates.

Procedures for cDNA library screening. Screens for cDNAs encoding proteases (Caspases). To screen for cDNAs encoding proteases capable of cleaving the S1 site (WEHD), the yeast strain EGY48 containing 6op-*LEU2*/2op-*lacZ* was transformed with p413-TEF-Fas-d-S1-TA followed selection on histiodine deficient plates, then these cells were subsequently transformed with a HEK293 cDNA library (contains TRP1 marker) or a human placenta cDNA library (contains TRP1 marker). The transformants were seeded on tryptophan-deficient growth plates (SD medium containing 2% glucose and 50 μg/ml leucine). Independent colonies of 5.1×10^5 appeared from the HEK293 cDNA library in 48 hours. The colonies were harvested and pooled, and a portion of the recovered cells (3.6×10^6 cells) was seeded onto leucine-deficient selection plates (SD medium containing 1% galactose, 0.2% raffinose, BU salts, and 80 μg/ml X-gal). Blue-colored colonies appeared within a week and were subjected to plasmid DNA extraction. The extracted plasmid DNAs were introduced into KC8 *E.coli* cells by electroporation to efficiently recover the cDNAs plasmids. The candidate cDNAs were again introduced into the yeast cells containing 6op-*LEU2*/2op-*lacZ*/TEF-Fas-d-S1-TA or 6op-*LEU2*/2op-*lacZ*/TEF-Fas-d-G1-TA to confirm whether they cleave S1 specifically.

To screen for cDNAs encoding proteases capable of cleaving the S3 site (DEVD), the yeast strain EGY48 containing 6op-*LEU2*/2op-*lacZ*, was transformed with p413-TEF-Fas-d-S3-TA, followed by selection on histiodine-deficient plates, and subsequently transformed with a HEK293 cell cDNA library, then processed as above.

Screening for cDNAs encoding proteins that activate Caspase-8 in a FADD-dependent manner. The yeast strain EGY48 (6op-*LEU2*) was transformed with plasmids p426-2op-*lacZ*/ΔADH1-FADD and p413-TEF-Fas-d-S8-TA/CYC1-Caspase8, then subsequently transformed with a HepG2 cDNA library, a human liver cDNA library, or a HEK293 cDNA library. The transformants were seeded on growth plates (SD medium containing 2% glucose and 50 μg/ml leucine). Independent colonies of 1.7×10^6 appeared from the HepG2 library in 48 hours. They were harvested, and pooled, and a portion of the cells (2.4×10^7 cells) was seeded on selection plates (SD medium containing 1% galactose, 0.2% raffinose, BU salts, and 80 μg/ml X-gal). Blue-colored colonies appeared within a week, were subjected to plasmid DNA extraction. The extracted plasmid DNAs were introduced into KC8 *E.coli* cells by electroporation to recover the cDNAs plasmids. The candidate cDNAs were again introduced into the yeast cells containing 6op-*LEU2*/2op-*lacZ* and plasmids containing transcriptional units for ΔADH1-FADD, TEF-Fas-d-S8-TA, and CYC1-Caspase8; or TEF-Fas-d-S8-TA and CYC1-Caspase8; or TEF-Fas-d-S8-TA to confirm whether they activate Caspase-8 in a FADD-dependent manner.

<u>Screening for cDNA encoding adapter proteins that link Fas or DR5 to Caspases-8 and -10.</u> The yeast strain EGY48 (6op-*LEU2*) was transformed with p426-2op-*lacZ*/ADH-DR5-FLAG and p413-TEF-Fas-d-S8-TA/CYC1-Caspase8, then subsequently transformed with a HeLa cell cDNA library or a HEK293 cDNA library. The transformants were seeded on growth plates (SD medium containing 2% glucose and 50 μg/ml leucine). Independent colonies of $1.0x10^6$ appeared from the HeLa cell cDNA library within 48 hours. They were harvested, pooled, and a portion of the cells ($2.4x10^7$ cells) was seeded on selection plates (SD medium containing 1% galactose, 0.2% raffinose, BU salts, and 80 μg/ml X-gal). Blue-colored colonies appeared within a week and were subjected to plasmid DNA extraction. The extracted plasmid DNAs were introduced into KC8 *E.coli* cells by electroporation to recover the cDNA plasmids. The candidate cDNAs were again introduced into the yeast cells expressing 6op-*LEU2*/2op-*lacZ*/ADH-DR5-FLAG/TEF-Fas-d-S8-TA/CYC1-Caspase8, 6op-*LEU2*/2op-*lacZ*/TEF-Fas-d-S8-TA/CYC1-Caspase8, or 6op-*LEU2*/2op-*lacZ*/TEF-Fas-d-S8-TA to confirm whether they activate Caspase-8 in a DR5-dependent manner.

The yeast strain EGY48 (6op-*LEU2*) was transformed with p426-2op-*lacZ*/ADH-Fas and p413-GPD-Fas-d-S8-TA/CYC1-Caspase10, then subsequently transformed with a HEK293 cell cDNA library. The transformants were seeded on growth plates (SD medium containing 2% glucose and 50 μg/ml leucine). Independent colonies of $2.2x10^6$ appeared within 48 hours, and were harvested, and pooled. A portion ($3.2x10^7$ cells) of the cells seeded on selection plates (SD medium containing 1% galactose, 0.2% raffinose, BU salts, and 80 μg/ml X-gal). Blue-colored colonies appeared in a week and were subjected to plasmid DNA extraction. The extracted plasmid DNAs were

introduced into KC8 *E.coli* cells by electroporation to recover the cDNA plasmids. The candidate cDNAs were again introduced into the yeast cells expressing 6op-*LEU2*/2op-*lacZ*/ADH-Fas/GPD-Fas-d-S8-TA/CYC1-Caspase10, 6op-*LEU2*/2op-*lacZ*/GPD-Fas-d-S8-TA/CYC1-Caspase10, or 6op-*LEU2*/2op-*lacZ*/GPD-Fas-d-S8-TA to confirm whether they activate Caspase-10 in a Fas-dependent manner.
