# Supplementary material for: Versatile Assays for High Throughput Screening for Activators or Inhibitors of Intracellular Proteases and Their Cellular Regulators
Source: PLoS One. 2009 Oct 30;4(10):e7655. doi: 10.1371/journal.pone.0007655 (PMC2764853; doi:10.1371/journal.pone.0007655)
Supplement: Table S1 — Determination of IC50 values for zVAD-fmk inhibition of Caspases in yeast. Yeast expressing various Caspases alone (at high levels) or in combination with upstream activators (at low levels) and cleavable substrates containing appropriate tetrapeptides reorganized by these proteases were used in 384 well β-galactosidase activity assays to assess inhibition by zVAD-fmk. The compound was titrated into assays at various concentrations and percentage inhibition was determined. IC50 values were determined, using PRIZM software for analysis. (0.01 MB PDF) [file pone.0007655.s002.pdf]

Table I. IC_{50} values for z-VAD inhibition of Caspases

Enzyme	IC_{50} (uM)
Caspase1	3.07
Caspase1 activated with Asc	1.00
Caspase6	6.78
Caspase7	4.11
Caspase8 activated with FADD + Fas	5.42
Caspase9 activated with Apaf*	10.66
Caspase10 activated with FADD	9.52

Table S1-(Reed)
