# Supplementary figures and images for: Versatile Assays for High Throughput Screening for Activators or Inhibitors of Intracellular Proteases and Their Cellular Regulators
Source: PLoS One. 2009 Oct 30;4(10):e7655. doi: 10.1371/journal.pone.0007655 (PMC2764853; doi:10.1371/journal.pone.0007655)

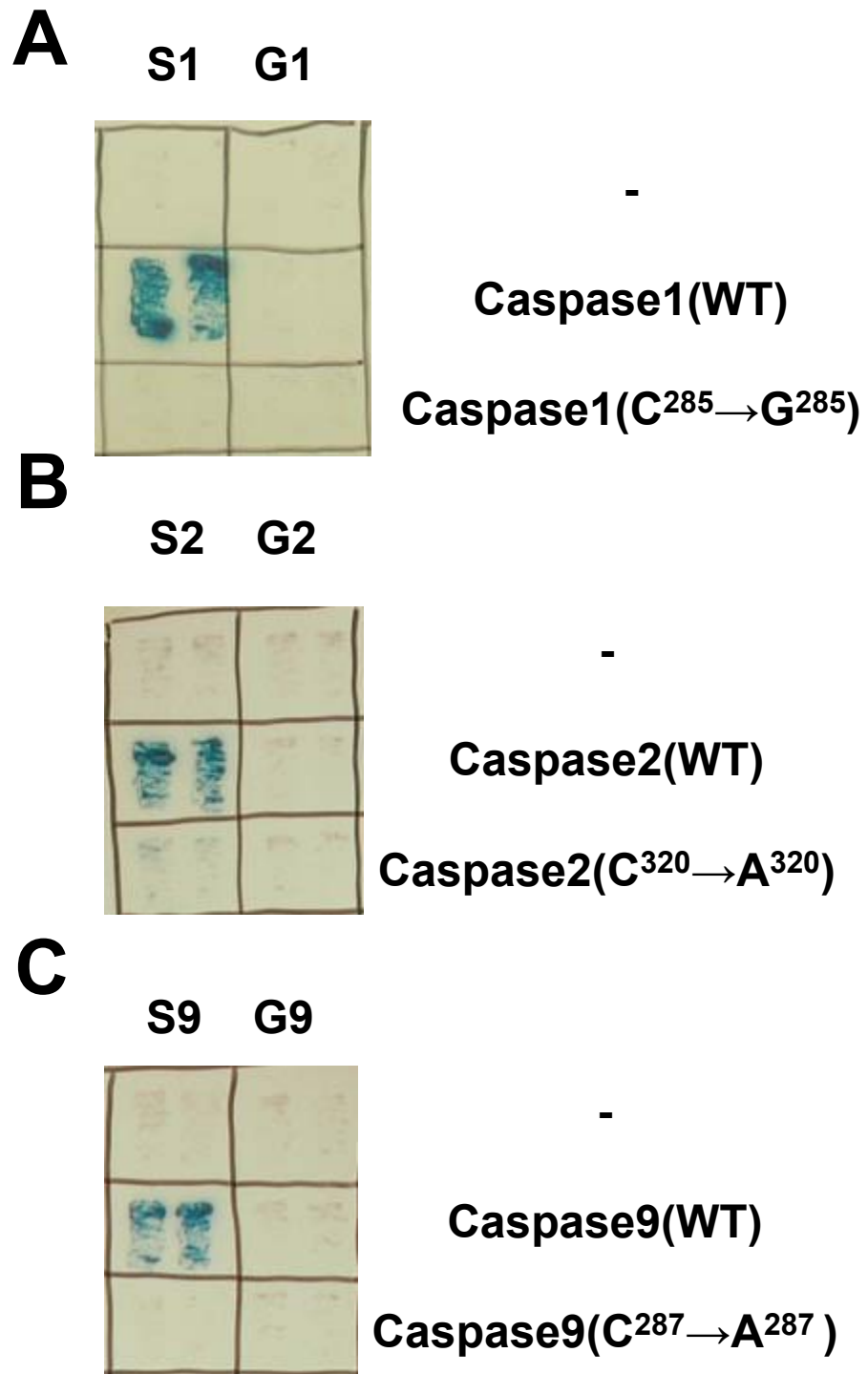

Figure-S1 (Reed)

Supplement: Figure S1 — Substrate sequence- and Caspase activity-dependent cleavage of the S1, S2, and S9. Yeast transformants were plated on leucine-deficient medium containing X-gal. (A) The recipient yeast cell strains, EGY48-6op-LEU2/2op-lacZ/TEF-Fas-d-S1(WEHD)-TA (S1), or EGY48-6op-LEU2/2op-lacZ/TEF-Fas-d-G1(WEHG)-TA (G1) were transformed with the plasmids encoding the active wild type Caspase1 (WT), the catalytically-defective Caspase1 (C285→G285), and the empty vector (-). (B) The recipient yeast cell strains, EGY48-6op-LEU2/2op-lacZ/TEF-Fas-d-S2(DEHD)-TA (S2), or EGY48-6op-LEU2/2op-lacZ/TEF-Fas-d-G2(DEHG)-TA (G2) were transformed with the plasmids encoding the active wild type Caspase2 (WT), the catalytically-defective Caspase2 (C320→A320), and the empty vector (-). (C) The recipient yeast cell strain, EGY48-6op-LEU2/2op-lacZ/TEF-Fas-d-S9(LEHD)-TA (S9), or EGY48-6op-LEU2/2op-lacZ/TEF-Fas-d-G9(LEHG)-TA (G9), were transformed with the plasmids encoding the active wild type Caspase9 (WT), the catalytically-defective Caspase9 (C287→A287), and the empty vector (-). (0.05 MB PDF) [file pone.0007655.s003.pdf]

**EGY48-6op-LEU2/2op-lacZ/TEF-Fsa-d-S1-TA**

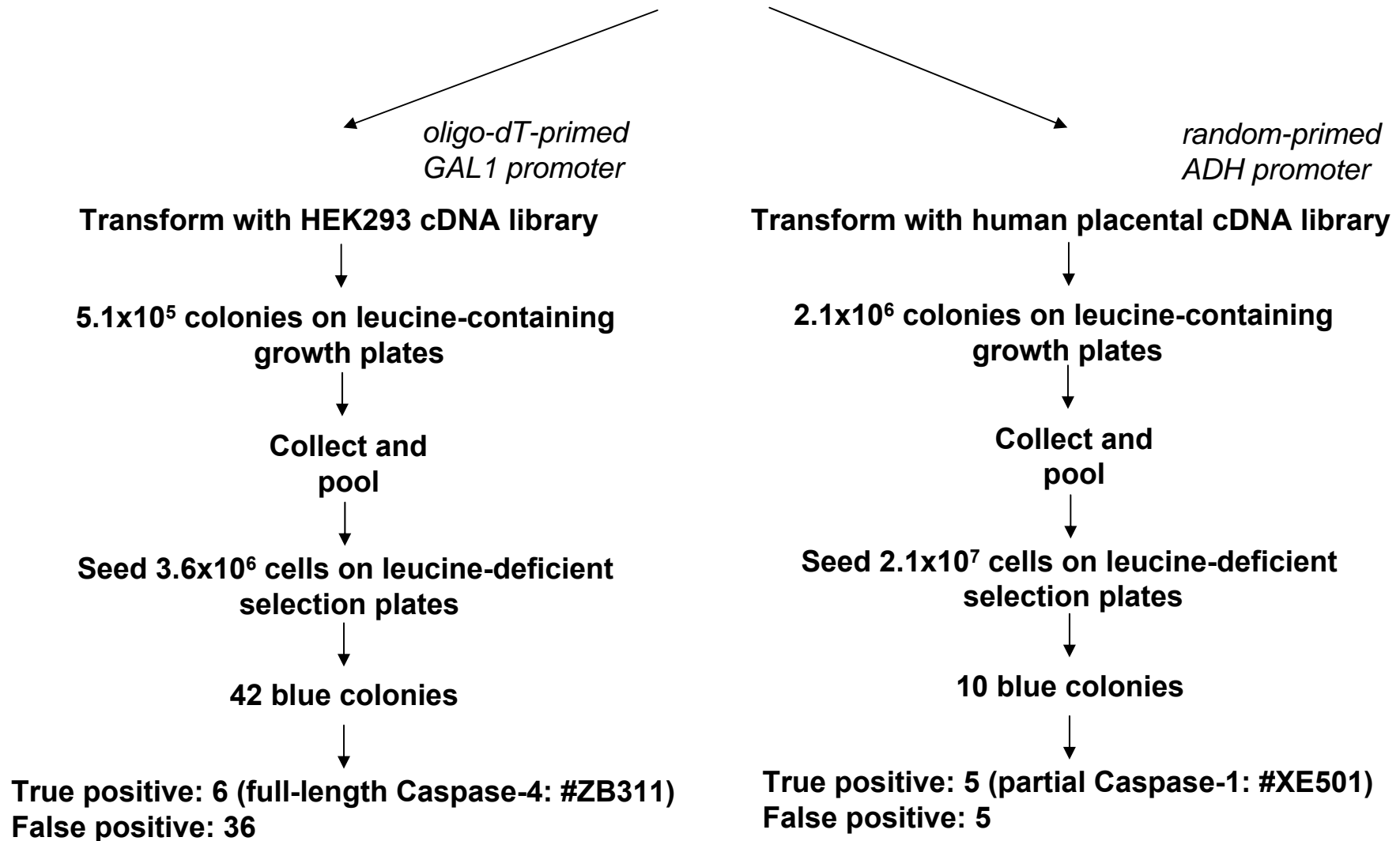

Figure-S2 (Reed)

Supplement: Figure S2 — Flow chart for cDNA library screening using a reporter gene strategy based on cleavable transcription factor. The example here is S1 substrate. The yeast strain EGY48 containing 6op-LEU2 and 2op-lacZ reporter genes and Caspase-cleavable substrate (expressed from TEF-Fas-d-S1-TA) was transformed (left) with a HEK293 cell cDNA library (oligo-dT-primed, PGAL1 promoter). Independent colonies of 5.1×10^5^ clones appeared on growth plates in 48 hours. Cells were collected, pooled, and a portion (3.6×10^6^) was seeded on leucine-deficient selection plates containing X-gal. Blue-colored colonies (n = 42) appeared within a week, of which 6 corresponded to clone #ZB311 encoding full-length Caspase-4 (which is known to cleave WEHD tetrapeptide [Thornberry, N.A, et. al., J. Biol. Chem. 272, 17907–17911 (1997)]), while the rest were false positives. (Right) Another cDNA library screen (HEK293 random-primed, ADH promoter used to drive expression) resulted in 2×10^6^ clones, which were pooled and 2.1×10^7^ cells were screened on leucine-deficient, X-gal-containing plates, resulting in 10 blue colonies. Five clones (including #XE501) encoding a fragment of Casapse-1 (L89-G403) were isolated. The remaining five clones were apparent false positives. (0.04 MB PDF) [file pone.0007655.s004.pdf]

**EGY48-6op-LEU2/2op-lacZ/TEF-Fsa-d-S3-TA**

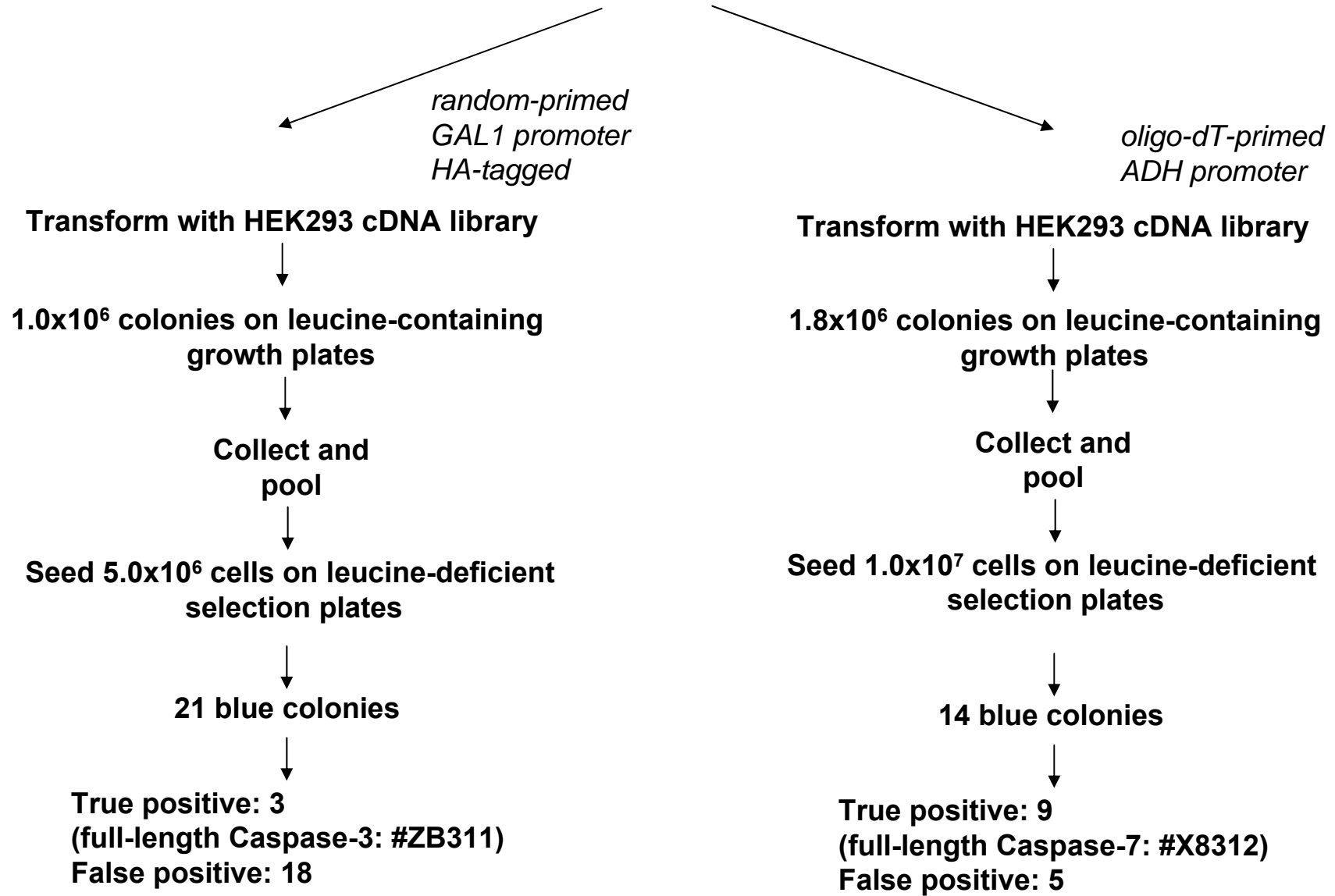

Figure-S3 (Reed)

Supplement: Figure S3 — Flow chart for cDNA library screening using reporter gene strategy based on cleavable transcription factor. The example here is S3/S7 substrate. Yeast strain EGY48 containing 6op-LEU2 and 2op-lacZ reporter genes and the DEVD-containing cleavable transcription factor (expressed from TEF-Fas-d-S3-TA) was transformed with a HEK293 cell cDNA library (random-primed, PGAL1 promoter, HA-tagged) (left). Independent colonies (1.0×10^6^) appeared on growth plates within 48 hours. Cells were collected, pooled, and a portion (5.0×10^6^) was seeded on the leucine-deficient selection plates containing X-gal. Blue-colored colonies (n = 21) appeared within a week, of which 3 clones (including #ZB331) encoded full-length Caspase-3, while the rest were false positives. In a similar experiment (right), another HEK293 cDNA library (oligo-dT-primed, PADH promoter) (1.8×10^6^ initial colonies) was screened, resulting in 9 positive clones (including #X8312) encoding full-length Caspase-7. The remaining five clones were false positives. Both cloned Caspases are known to cleave DEVD tetrapeptide [Thornberry, N.A, et. al., J. Biol. Chem. 272, 17907–17911 (1997)]. (0.04 MB PDF) [file pone.0007655.s005.pdf]

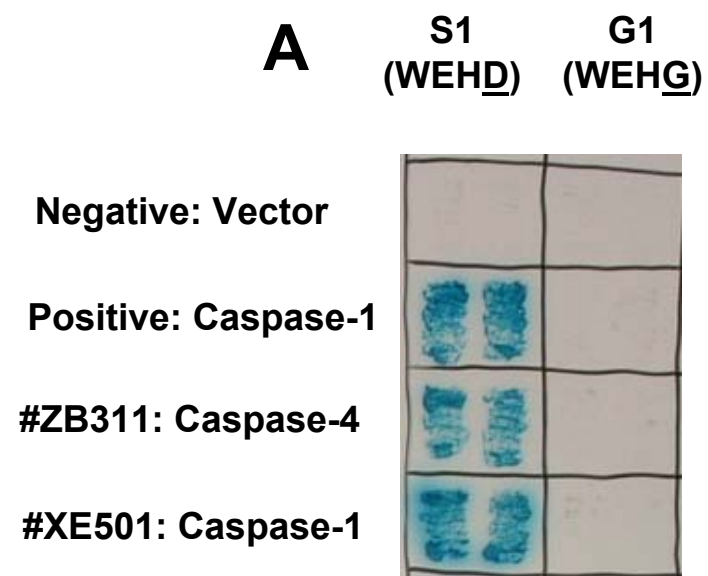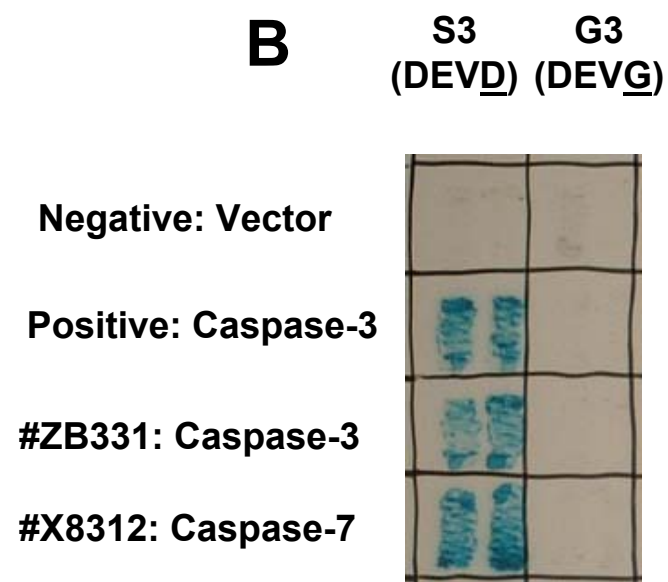

Figure-S4 (Reed)

Supplement: Figure S4 — Use of one-component yeast-based Caspase activity assay for cDNA library screening. (A) The plasmids containing library cDNAs from Figure S2 were recovered and re-transformed into yeast cells containing 6op-LEU2 and 2op-lacZ reporter genes with either cleavable (WEHD-containing) (expressed from TEF-Fas-d-S1-TA) or non-cleavable (WEHG-containing) transcription factor substrate (expressed from TEF-Fas-d-G1-TA) to confirm whether they cleave S1 specifically. As controls, yeast were transformed with a plasmid encoding Caspase-1 (“Positive” control) or the empty vector (“Negative” control). Assays were performed in duplicate, with cells grown on selection plates for 4 days. Note that the cDNA library clones supported lacZ reporter gene activation only when co-expressed with the S1 cleavable substrate. (B) The cDNA library plasmids from Figure S3 were recovered and used to re-transform the yeast strains containing either the same cleavable DEVD-containing or DEVG non-cleavable transcription factor, expressed from TEF-Fas-d-S3-TA (“S3” substrate) and TEF-Fas-d-G3-TA (“G3” substrate), respectively. Assays were performed in duplicate and cells grown on selection plates for 4 days. Note that the two cDNA library clones activated the lacZ reporter gene only when co-expressed with cleavable S3 (DEVD-containing) substrate. As controls, yeast cells were transformed with plasmids encoding Caspase-3 (“Positive” control) or the empty vector (“Negative” control). (0.04 MB PDF) [file pone.0007655.s006.pdf]

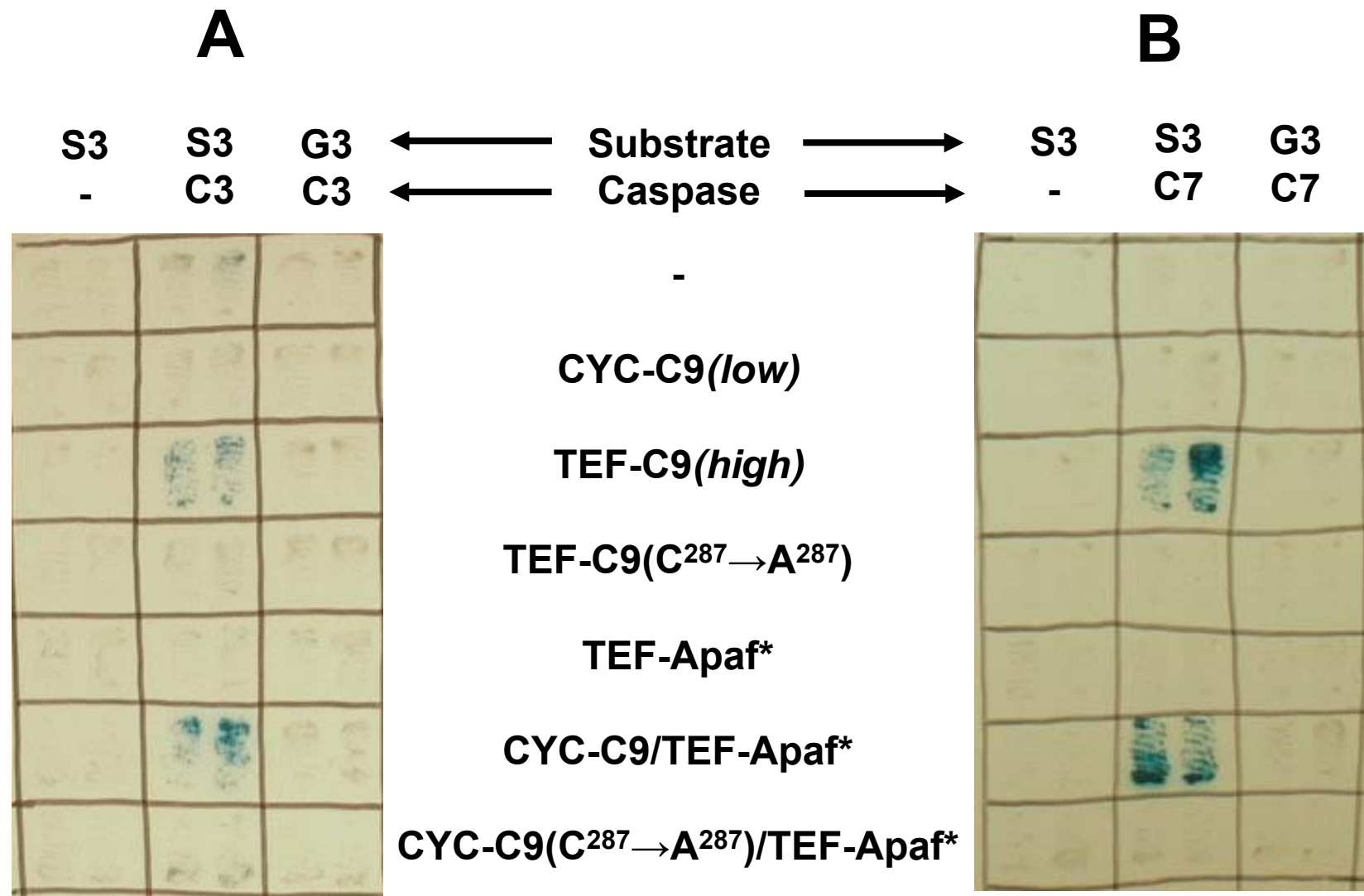

Figure-S5 (Reed)

Supplement: Figure S5 — Validation of yeast-based assays for effector Caspase activators - two- and three-component systems. Yeast EGY191 strain containing 2op-LEU2/2op-lacZ or EGY48 strain containing 6op-LEU2/2op-lacZ were employed for developing assays for activators of downstream effector Caspases (e.g. Caspases-3 and -7). Yeast transformants were plated on leucine-deficient medium containing X-gal. Yeast were transformed with plasmids encoding membrane tethered transcription factor substrate with either DEVD-containing cleavable (S3) or DEVG-containing non-cleavable (G3) linkers, and with plasmids encoding pro-Caspase-3 (C3) or pro-Caspase-7 (C7) or the corresponding empty vector (-). (Note that the optimal tetrapeptide sequence for both Caspase-3 and Caspase-7 has previously been reported to be DEVD). Yeast transformants were as follows: (A) S3 Substrate/No Caspase: EGY191-2op-LEU2/2op-lacZ/ΔTEF3-Fas-d-S3 (DEVD)-TA; S3 Substrate/Caspase-3: EGY191-2op-LEU2/2op-lacZ/ΔTEF3-Fas-d-S3/7(DEVD)-TA/ΔCYC2-HA-Caspase3; G3 Substrate/Caspase-3: EGY191-2op-LEU2/2op-lacZ/ΔTEF3-Fas-d-G3/7(DEVG)-TA/ΔCYC2-Caspase-3; and (B) S3 Substrate/No Caspase: EGY48-6op-LEU2/2op-lacZ/TEF-Fas-d-S3(DEVD)-TA; S3 Substrate/Caspase-7: EGY48-6op-LEU2/2op-lacZ/TEF-Fas-d-S3(DEVD)-TA/CYC1-Caspase-7; G3 Substrate/Caspase-7: EGY48-6op-LEU2/2op-lacZ/TEF-Fas-d-G3(DEVG)-TA/CYC1-Caspase-7. These yeast transformants were then subsequently transformed with plasmids expressing a small amount of Caspase-9 (driven from the CYC promoter) or plasmids expressing large amounts of wild-type (WT) or catalytically-defective (C287→A287) Caspase-9 (driven from the TEF promoter), with or without an active form (gain of function mutant not requiring cytochrome c for activation) of Apaf-1 (driven by TEF-promoter) or the corresponding empty vectors (-) (Haraguchi M, Torii S, Matsuzawa S, et al. J Exp Med 2000;191:1709-20). Note that the large amount of Caspase-9 (expressed from TEF promoter) activated the cleavable S3(DEVD) substrate when co-expr [file pone.0007655.s007.pdf]

Substrate: S1   S2   S8   S9   S8  
 Caspase: C1   C2   C8   C9   C10

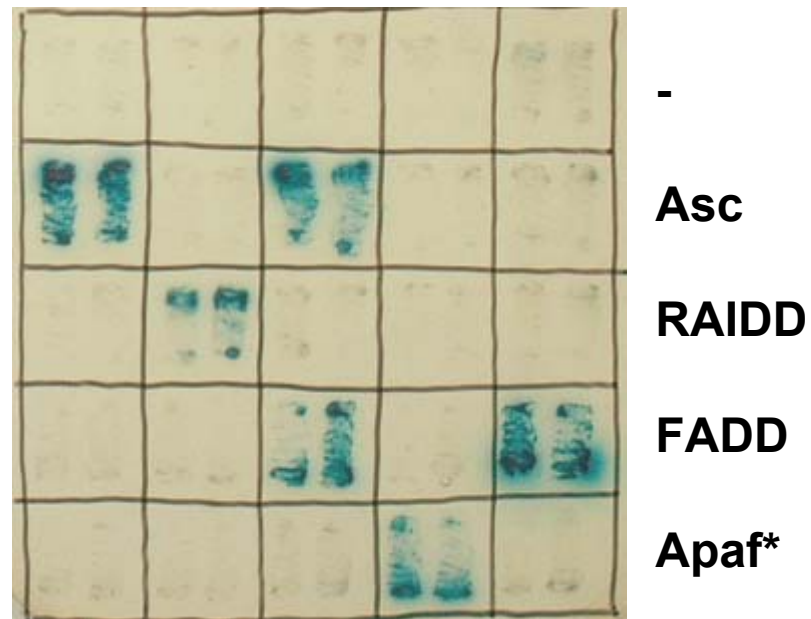

Figure-S7 (Reed)

Supplement: Figure S7 — Specificity of upstream activators of initiator Caspases - tested by 2-component systems. Yeast transformants were prepared and tested as described in Figures S5 and S6, except a matrix of plasmid combinations was prepared to evaluate the specificity of upstream activators. The substrates and Caspases tested are indicated across the top, while the activators are indicated along the side. Note that results obtained were as predicted, with (1) Apaf-1* activating pro-Caspase-9, but not other initiator Caspases; (2) RAIDD activating pro-Caspase-2, but not other Caspases; (3) FADD activating pro-Caspases-8 and 10, but not other Caspases, and (4) Asc activating pro-Caspase-1 and 8. Note that while Asc contains a CARD that pairs with a complementary CARD in pro-Caspase-1 and would not be necessarily predicted to activate the DED-containing protease Caspase-8, it has previously been reported that Asc is an activator of Caspase-8 (Hasegawa M. et.al. J Biol Chem 280:15122-30 (2005); Masumoto, J. et. al., Biochem. Biophys. Res. Commun 303: 69-73 (2003). Transformants: Transformed yeast clones were as follows: S1,C1:EGY48-6op-LEU2/2op-lacZ/TEF-Fas-d-S1(WEHD)-TA/ΔTEF3-Caspase1-FLAG; S2,C2:EGY48-6op-LEU2/2op-lacZ/ΔTEF2-Fas-d-S2(DEHD)-TA/ΔGPD1-HA-Caspase2-FLAG; S8,C8:EGY48-6op-LEU2/2op-lacZ/GPD-Fas-d-S8/10(LETD)-TA/CYC1-Caspase8-HA; S9,C9:EGY48-6op-LEU2/2op-lacZ/TEF-Fas-d-S9(LEHD)-TA/TEF-HA-Caspase-9; S8,C10:EGY48-6op-LEU2/2op-lacZ/GPD-Fas-d-S8/10(LETD)-TA/ADH-Caspase10- FLAG. These cells were transformed with the plasmids encoding the activators (Asc, RAIDD, FADD, and Apaf*). For controls (-), the “empty” version of the plasmids were introduced. (0.05 MB PDF) [file pone.0007655.s009.pdf]

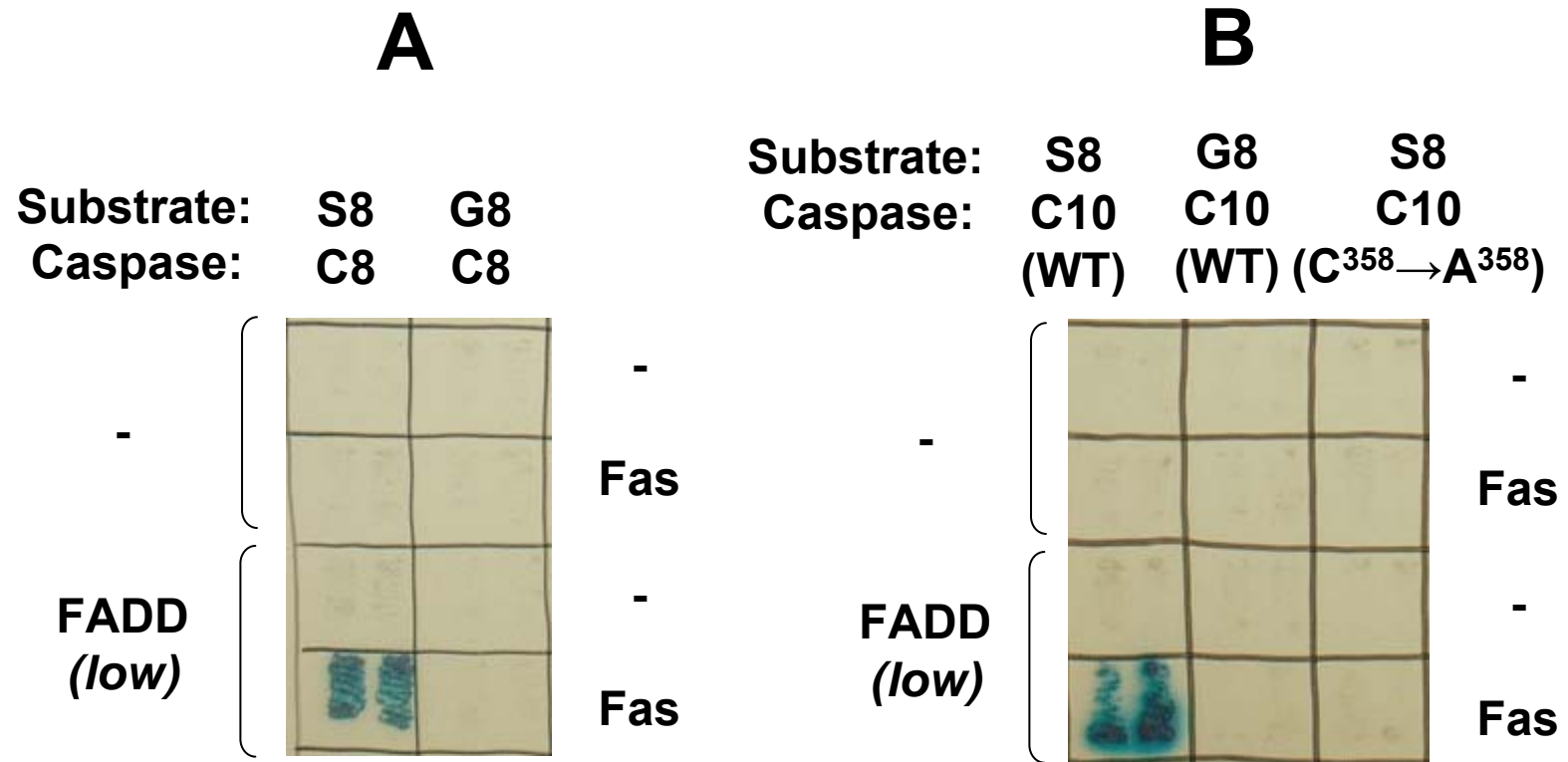

Figure-S8 (Reed)

Supplement: Figure S8 — Validation of 3-component yeast-based Caspase assay reconstituting DISC. Yeast transformants were prepared to assess the performance of the Fas/FADD/Caspase-8/10 three-component systems. Two independent clones of each transformant were plated on leucine-deficient medium containing X-gal. Substrates included LETD-containing cleavable (S8) and LETG-containing non-cleavable (G8) transcription factors, while Caspase expression plasmids included WT pro-Caspases-8 (A) and -10 or a catalytic mutant of Caspase-10. (B) FADD was expressed at low levels from a ΔADH1 promoter. Note that the lacZ reporter gene was activated only when the combination of Fas, FADD, and either WT pro-Caspase-8 or -10 was co-expressed and only when a cleavable substrate (S8) was employed. (Note that the optimal tetrapeptide cleavage sequence is the same for Caspases-8 and -10). Transformants: (A) Yeast transformants included EGY48-6op-LEU2/2op-lacZ/TEF-Fas-d-S8 (LETD)-TA/CYC1-Caspase-8-HA (S8,C8) EGY48-6op-LEU2/2op-lacZ/TEF-Fas-d-G8(LETG)-TA/CYC1-Caspase8-HA (G8,C8), without (-) or with Fas, and without (-) or with FADD, which were expressed from either ADH and ΔADH1 promoter, respectively, to achieve high expression of Fas and low expression of FADD. (B) Yeast transformants included: EGY48-6op-LEU2/2op-lacZ/GPD-Fas-d-S8(LETD)-TA/CYC1-Caspase10-FLAG (S8,C10); EGY48-6op-LEU2/2op-lacZ/GPD-Fas-d-G8(LETG)-TA/CYC1-Caspase10-FLAG (G8,C10); and EGY48-6op-LEU2/2op-lacZ/GPD-Fas-d-S8 (LETD)-TA/CYC1-Caspase10(C358→A358)-FLAG (S8,C10(C358→A358)) each without (-) or with Fas, and without (-) or with FADD-expressing vector or the corresponding empty vectors. (0.05 MB PDF) [file pone.0007655.s010.pdf]

**EGY48-6op-LEU2/2op-lacZ/TEF-Fsa-d-S8-TA/CYC1-Caspase-8-HA/ $\Delta$ ADH1-FADD**

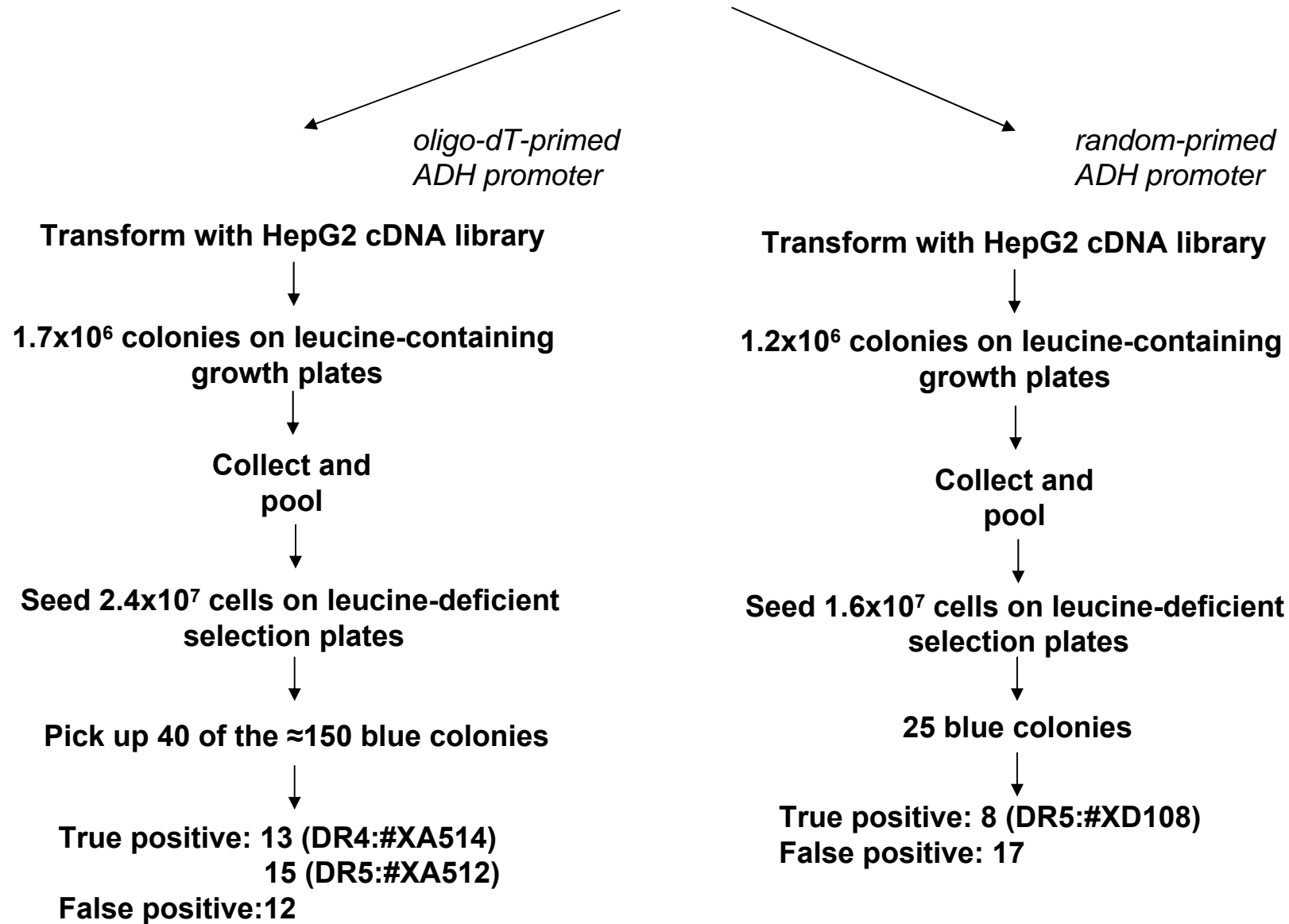

Figure-S9 (Reed)

Supplement: Figure S9 — Flow chart for cDNA library screening using 3 component system - application to death receptor cloning-Example #1. The screening strategy is essentially the same as outlined in Figure 3. The yeast transformant EGY48-6op-LEU2/2op-lacZ/TEF-Fas-d-S8(LETD)-TA/CYC1-Caspase8-HA/ΔADH1-FADD was transformed with a HepG2 cell cDNA library (oligo dT-primed, PADH promoter). Independent colonies (1.7×10^6^) appeared on growth plates within 48 hours. Cells were collected, pooled, and a portion (2.4×10^7^) was seeded on the leucine-deficient selection plates containing X-gal. Blue-colored colonies (n = 150) appeared within a week, 13 of which encoded DR4, (#XA514), and 15 clones encoded DR5 variant 2 (#ZA512), while the rest were false positives. Screening another cDNA library from HepG2 cells (random-primed, PADH promoter) yielded eight DR5 (#XD108) clones. (0.05 MB PDF) [file pone.0007655.s011.pdf]

**EGY48-6op-LEU2/2op-lacZ/TEF-Fsa-d-S8-TA/CYC1-Caspase-8-HA/ $\Delta$ ADH1-FADD**

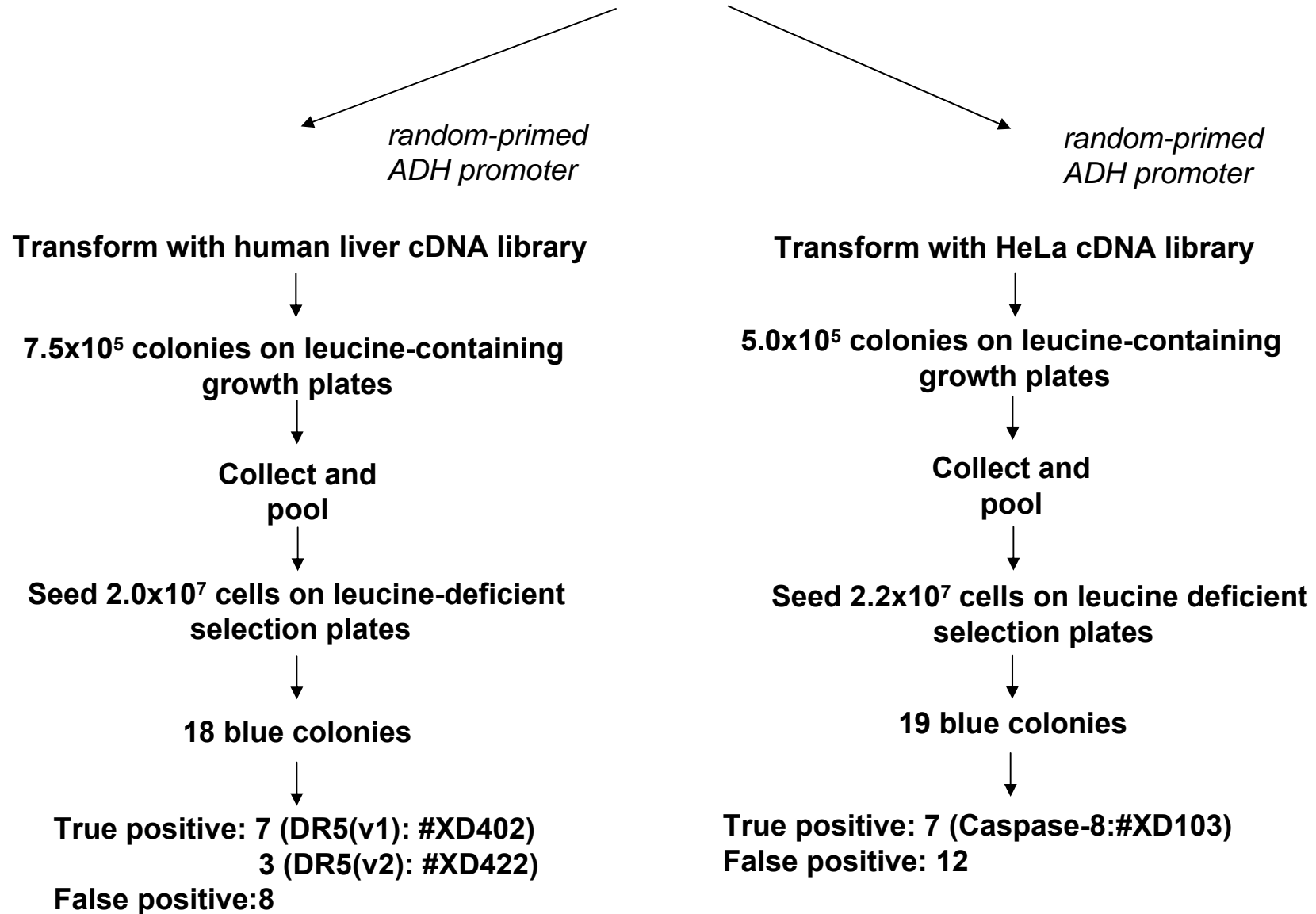

Figure-S10 (Reed)

Supplement: Figure S10 — Flow chart for cDNA library screening using 3 component system to clone death receptors - Example #2. The screening strategy is essentially the same as outlined in Figure 3. The yeast transformant EGY48-6op-LEU2/2op-lacZ/TEF-Fas-d-S8(LETD)-TA/CYC1-Caspase8-HA/ΔADH1-FADD was transformed with a human liver cDNA library (random-primed, PADH promoter) and a HeLa cell cDNA library (random-primed, PADH promoter). (0.04 MB PDF) [file pone.0007655.s012.pdf]

**EGY48-6op-LEU2/2op-lacZ/TEF-Fsa-d-S8-TA/CYC1-Caspase-8-HA/ $\Delta$ ADH1-FADD**

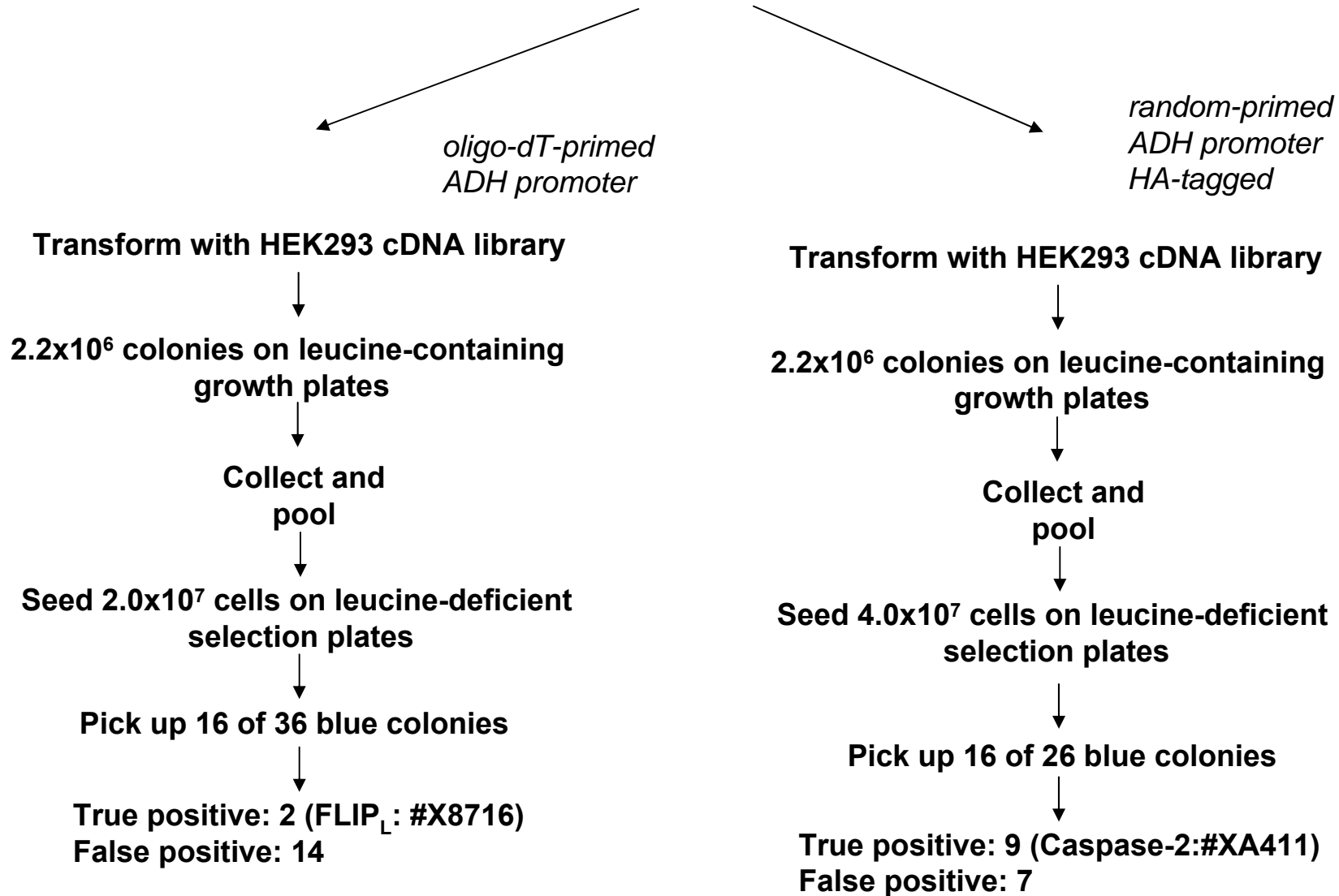

Figure-S11 (Reed)

Supplement: Figure S11 — Flow chart for cDNA library screening using 3-component system to to clone death receptors - Example #3. The screening strategy is essentially the same as outlined in Figure 3. The yeast transformant EGY48-6op-LEU2/2op-lacZ/TEF-Fas-d-S8(LETD)-TA/CYC1-Caspase8-HA/ΔADH1-FADD was transformed with a HEK293T cell cDNA library (oligo-dT-primed, PADH promoter) and a HEK293T cell cDNA library (random-primed, PADH promoter, HA-tagged). (0.04 MB PDF) [file pone.0007655.s013.pdf]

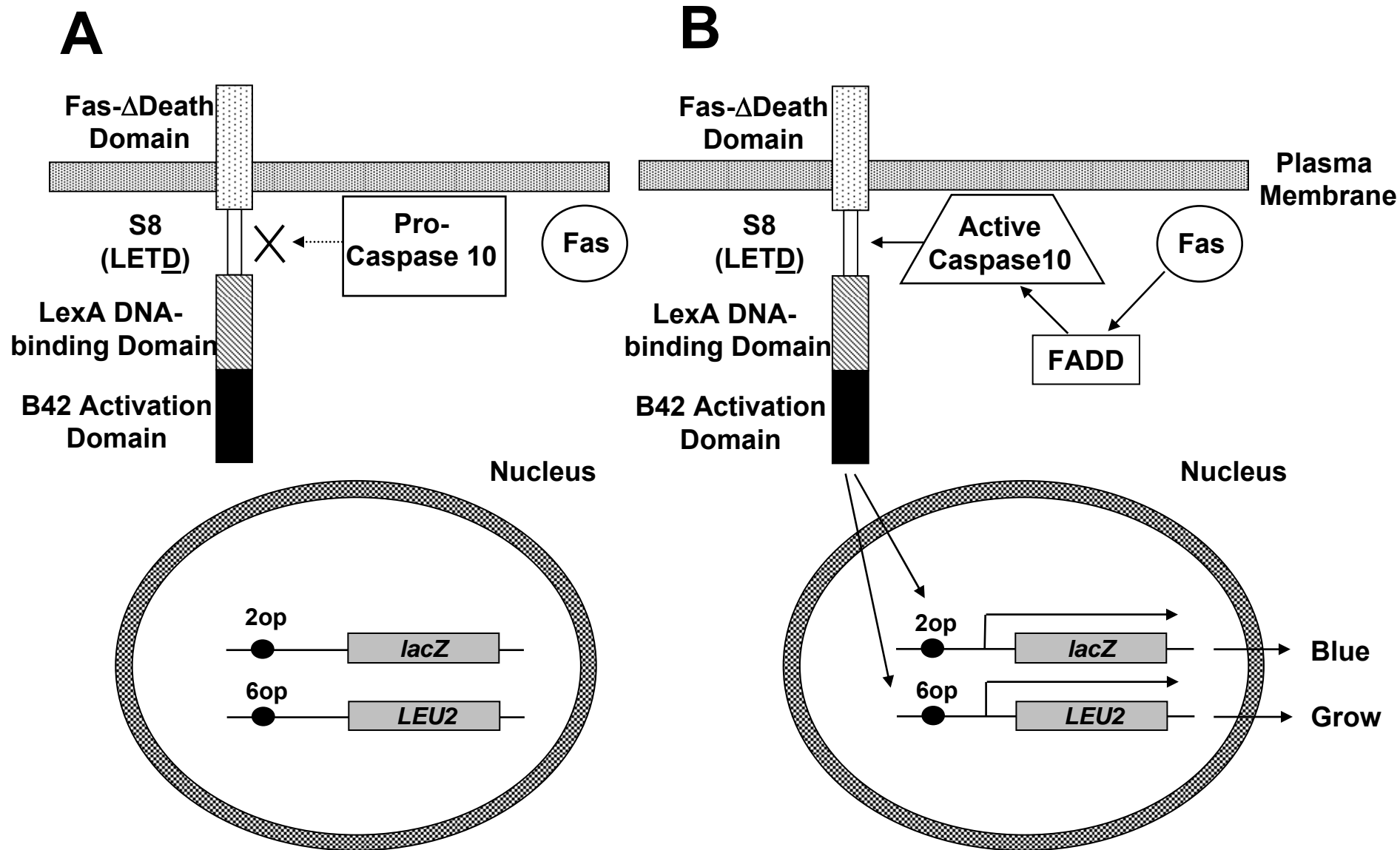

Figure-S12 (Reed)

Supplement: Figure S12 — Schematic representation of 3-component system used for cloning adapter protein that links Fas to pro-Caspase-10. (A) The zymogen pro-Caspase-10 was expressed (from CYC1-promoter) with substrate containing the LETD-containing cleavage element S8 (from plasmid p413-GPD-Fas-d-S8-TA/CYC1-Caspase-10-FLAG) in the yeast EGY48 expressing 6op-LEU2. Fas was also expressed without activating the Caspase-10 from plasmid p426-2op-lacZ/ADH-Fas. (B) Addition of FADD activates Caspase-10, releasing the transcription factor. (0.04 MB PDF) [file pone.0007655.s014.pdf]

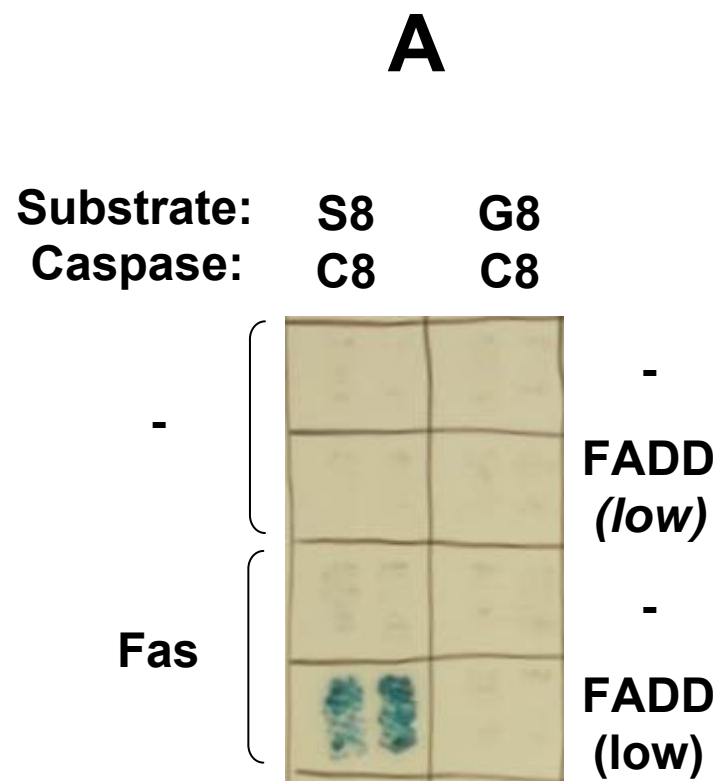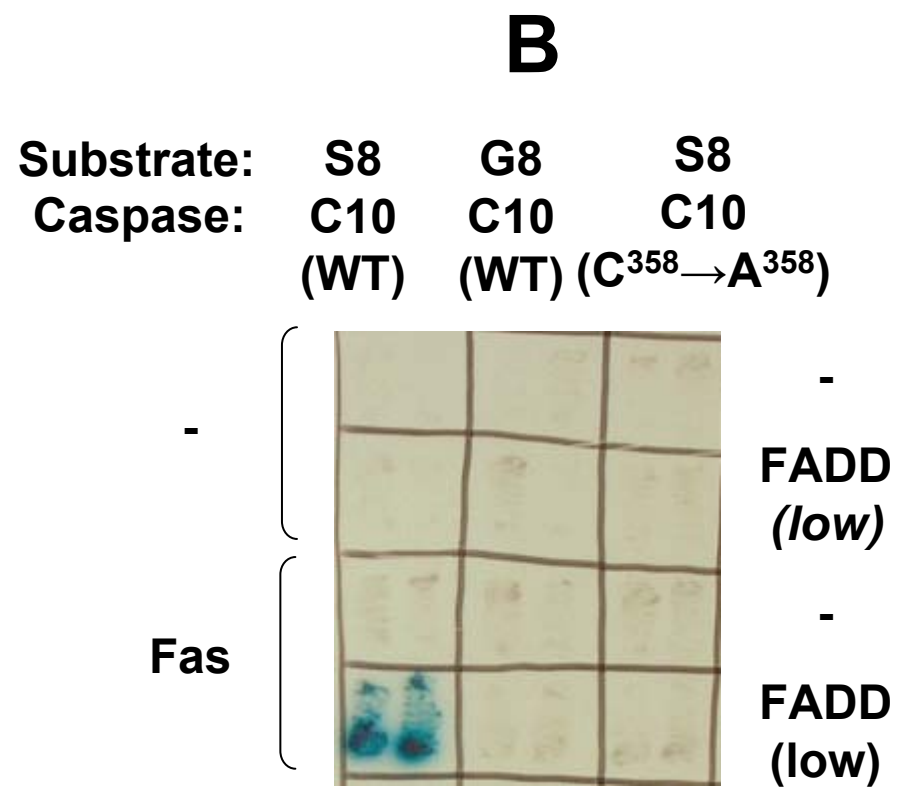

Figure-S13 (Reed)

Supplement: Figure S13 — Validation of adapter protein cloning system for Fas/FADD/Caspase-8/10: Reconstituted DISC. Yeast cell transformants were prepared to assess the performance of the Fas/FADD/Caspase-8 or Fas/FADD/Caspase-10 three-component systems. Two independent clones of each transformant were plated on leucine-deficient medium containing X-gal and grown for 4 days. Substrates included LETD-containing cleavable (S8) and LETG-containing non-cleavable (G8) transcription factors, while Caspase expression plasmids included wild-type (WT) pro-Caspases-8 (A) and -10 or a catalytic mutant of Caspase-10 (C358→A358) (B). Note that the lacZ reporter gene was activated only when the combination of Fas, FADD, and either WT pro-Caspase-8 or -10 was co-expressed and only when a cleavable substrate (S8) was employed. Transformants: (A) Yeast cell transformants included: EGY48-6op-LEU2/2op-lacZ/TEF-Fas-d-S8 (LETD)-TA/CYC1-Caspase8-HA (S8,C8) and EGY48-6op-LEU2/2op-lacZ/TEF-Fas-d-G8 (LETG)-TA/CYC1-Caspase8-HA (G8,C8), with empty vector (-) or with plasmids encoding FADD (expressed from CYC1 promoter) or Fas (expressed from ADH promoter). (B) Yeast cell transformants included EGY48-6op-LEU2/2op-lacZ/GPD-Fas-d-S8(LETD)-TA/CYC1-Caspase10-FLAG (S8,C10), EGY48-6op-LEU2/2op-lacZ/GPD-Fas-d-G8 (LETG)-TA/CYC1-Caspase10-FLAG (G8,C10), and EGY48-6op-LEU2/2op-lacZ/GPD-Fas-d-S8(LETD)-TA/CYC1-Caspase10(C358→A358)-FLAG (S8,C10(C358→A358)), each with empty vector (-) or with plasmids encoding FADD or Fas as above. (0.05 MB PDF) [file pone.0007655.s015.pdf]

**EGY48-6op-LEU2/2op-lacZ/GPD-Fsa-d-S8-TA/CYC1-Caspase-10-FLAG/ADH-Fas**

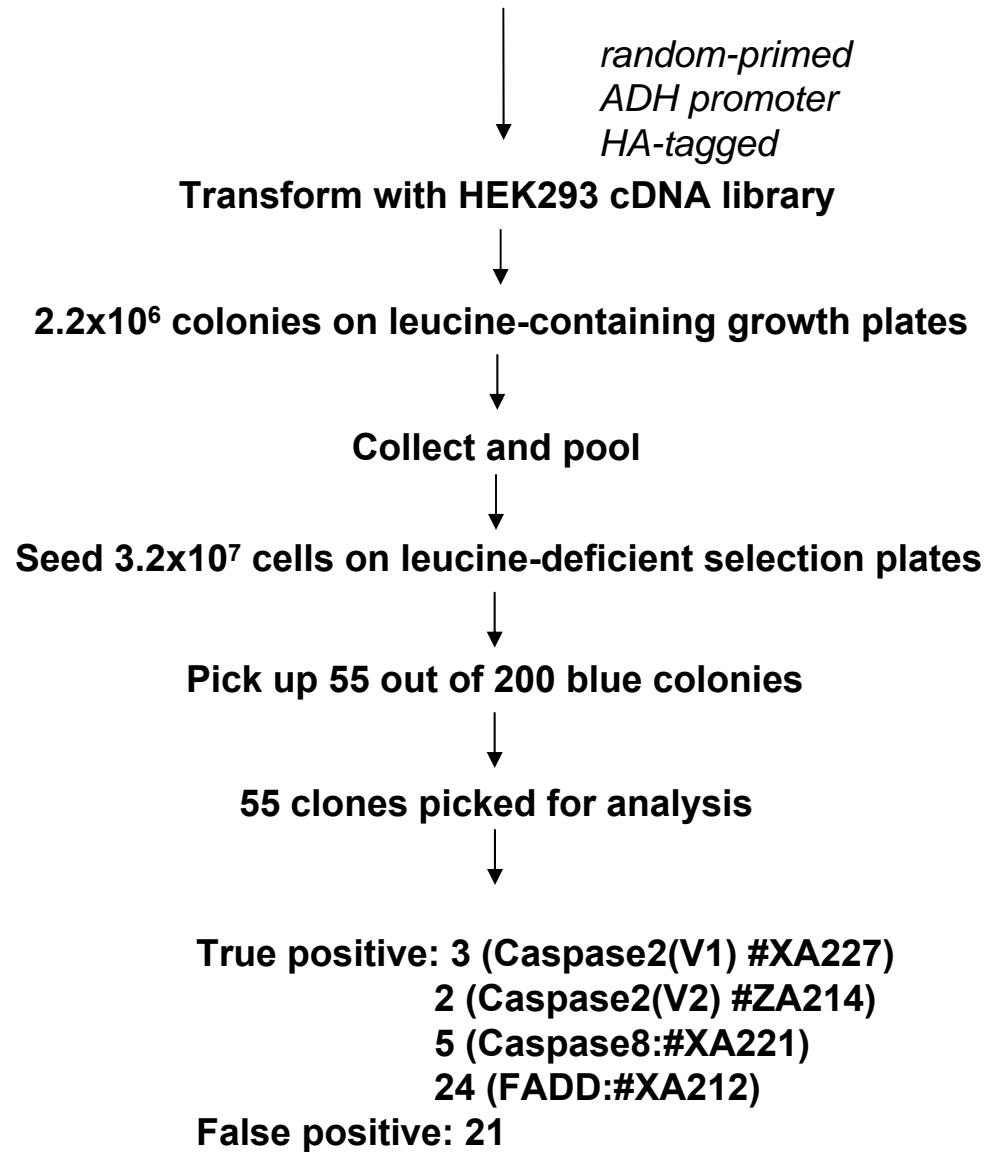

**Figure-S14 (Reed)**

Supplement: Figure S14 — Flow chart for cDNA library screening using 3 component system to clone adapters. Yeast transformant EGY48-6op-LEU2/2op-lacZ/GPD-Fas-d-S8(LETD)-TA/ADH-Fas/CYC1-Caspase10-FLAG was transformed with a HEK293 cell cDNA library (random-primed, PADH promoter, HA-tagged). Independent colonies (∼2.2×10^6^) appeared on growth plates within 48 hours. Cells were collected, pooled, and a portion (3.2×10^7^) was seeded on leucine-deficient selection plates containing X-gal. Blue-colored colonies (n = 200) appeared within a week, five of which encoded full-length Caspase-2 (#XA227), five encoded a fragment of Caspase-2 (#ZA214), five encoded a fragment of Caspase-8 (#XA221), and 24 encoded full-length FADD (#XA212), while the rest were apparent false positives. (0.03 MB PDF) [file pone.0007655.s016.pdf]

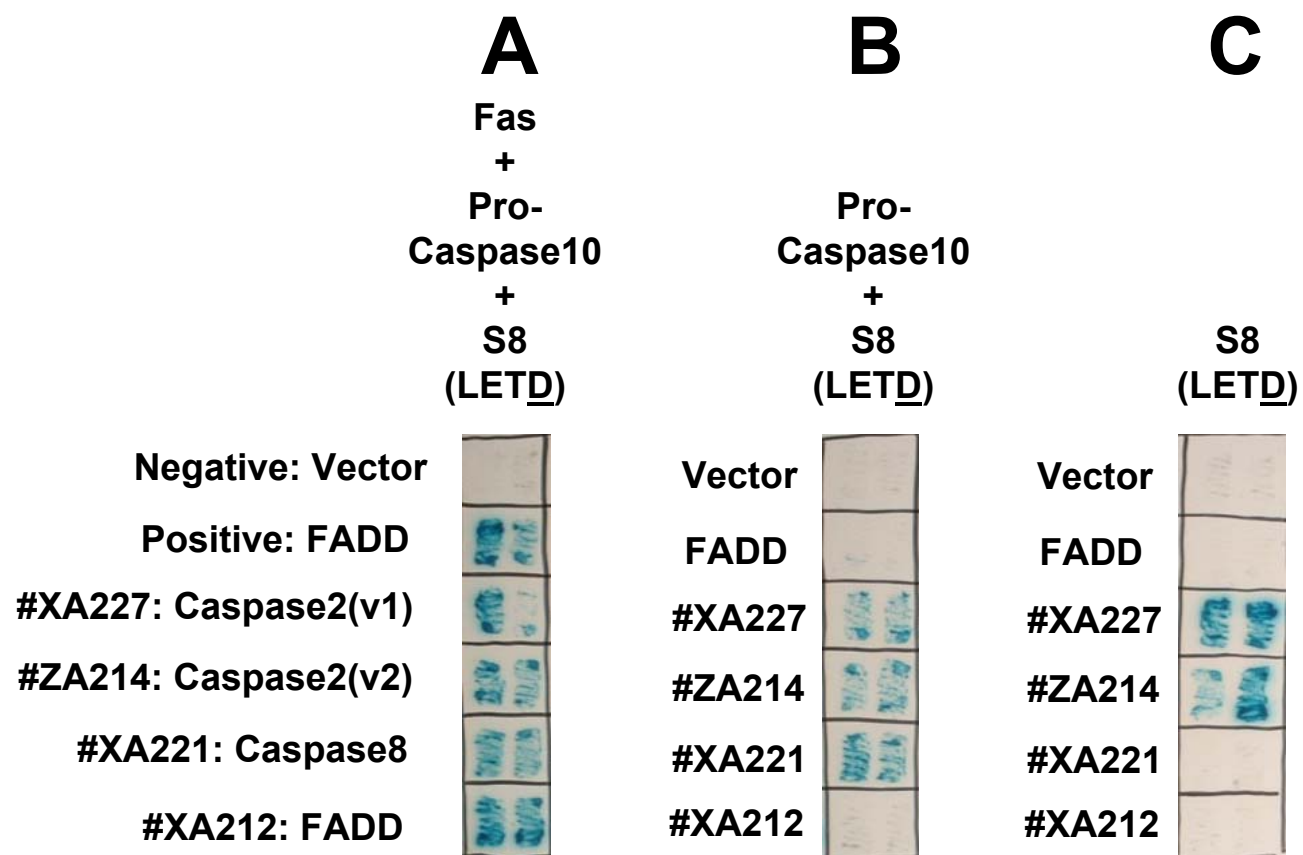

Figure-S15 (Reed)

Supplement: Figure S15 — Examples of cDNA cloning results. The clones that activated the reporter genes were characterized by recovery of cDNA library plasmids and retransformation into yeast expressing Fas and pro-Caspase-10 (A), pro-Caspase-10 without Fas (B), or neither (C). Among the positive clones were a full-length Caspase-2 (#XA227), a fragment of Caspase-2 (#ZA214, V130-L312), a fragment of Caspase-8 (#XA221, M1-K438), and full-length FADD (#XA212). Assays were performed in duplicate, growing cells for 4 days on plates. (0.05 MB PDF) [file pone.0007655.s017.pdf]

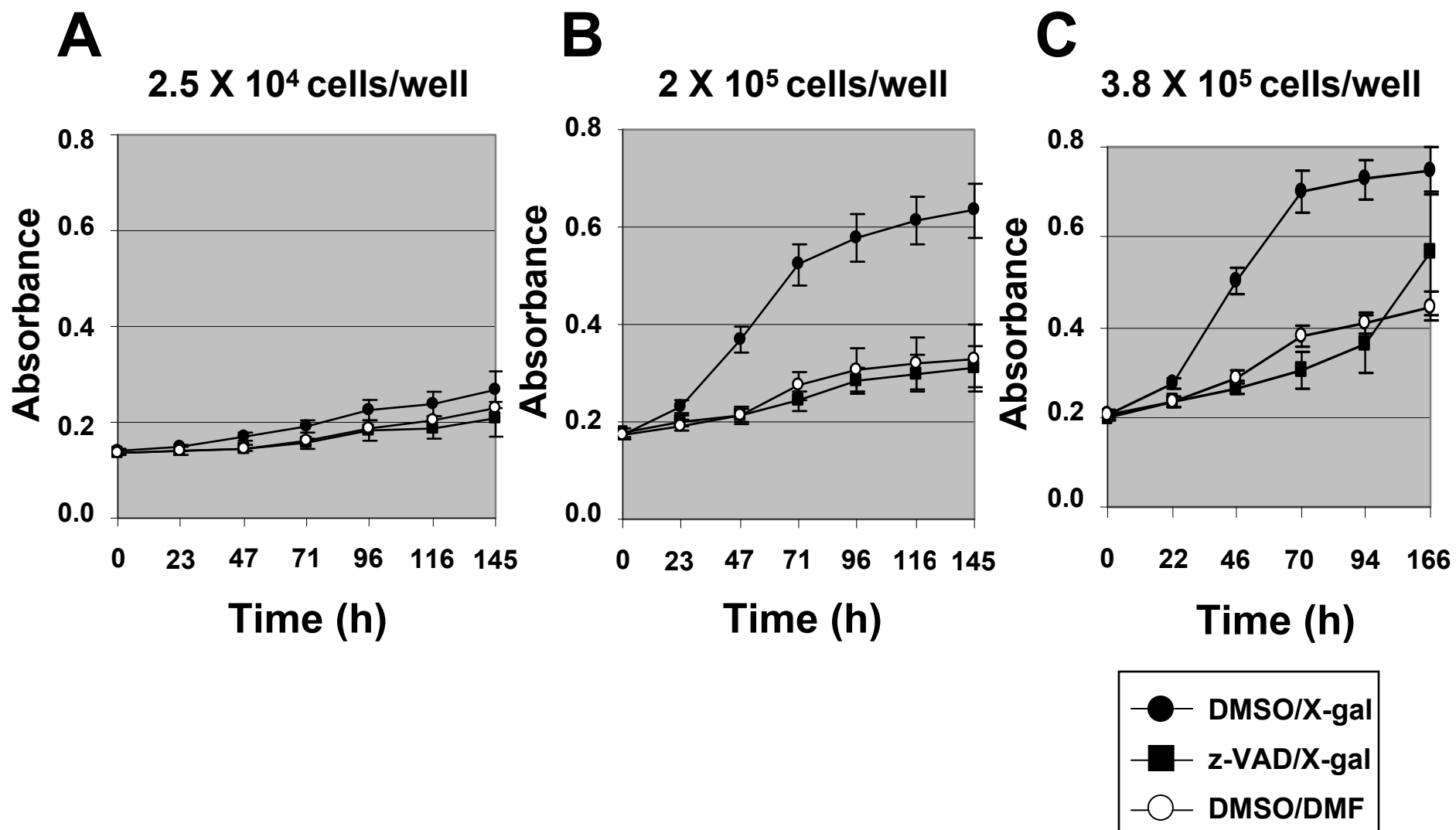

Figure-S16 (Reed)

Supplement: Figure S16 — Optimization of signal:noise ratio in microtiter plates: cell density. EGY48 cells containing the 3-component Fas/FADD/Caspase-8 system and Caspase-8-cleavable reporter were seeded at 2.35×10^4^/well (A), 2×10^5^/well (B), or 3.76×10^5^/well (C) in 384 well plates to compare cell densities. Cells were cultured at 30°C. The activity of β-galactosidase was measured after 72 hrs at various times after initiating cultures (mean+std dev; n = 3), for cells grown without (white circles) or with (black circles and black squares) X-gal and grown in the absence (circles) or presence (black squares) of 100 µM zVAD-fmk Caspase inhibitor. (0.04 MB PDF) [file pone.0007655.s018.pdf]

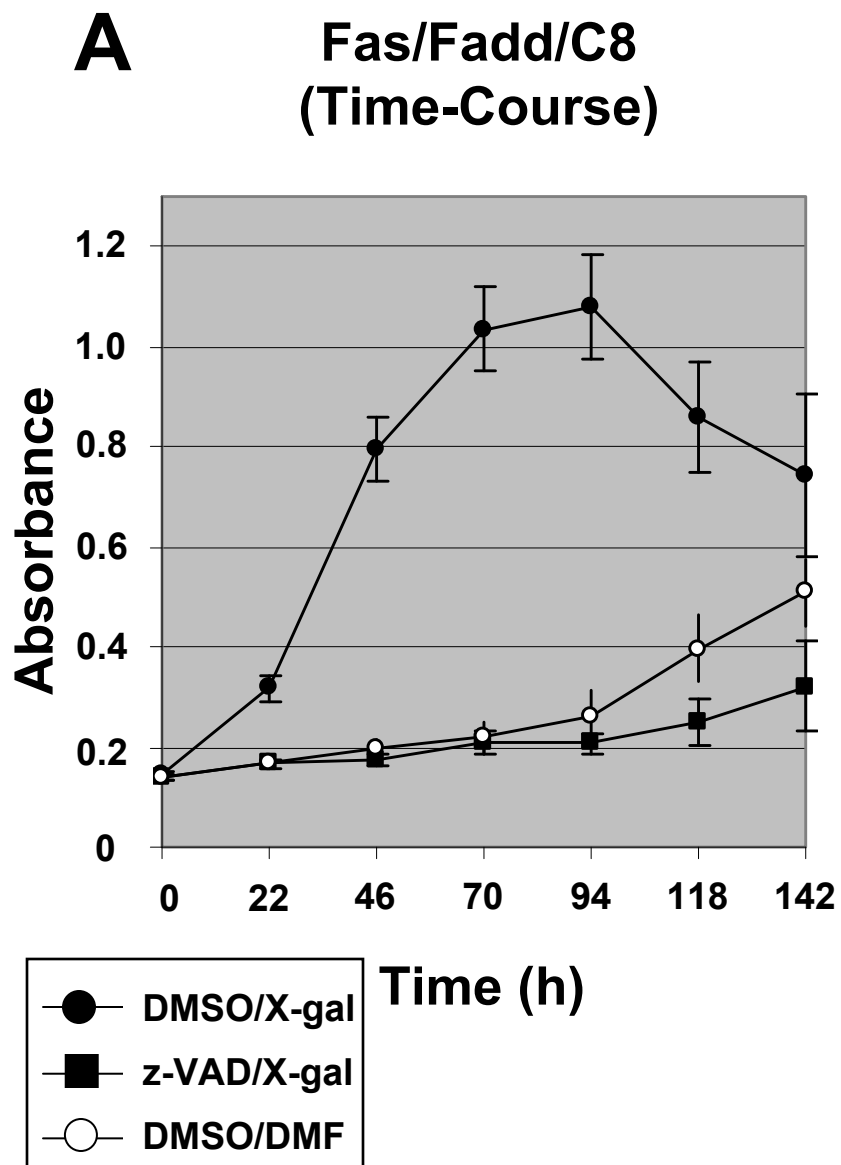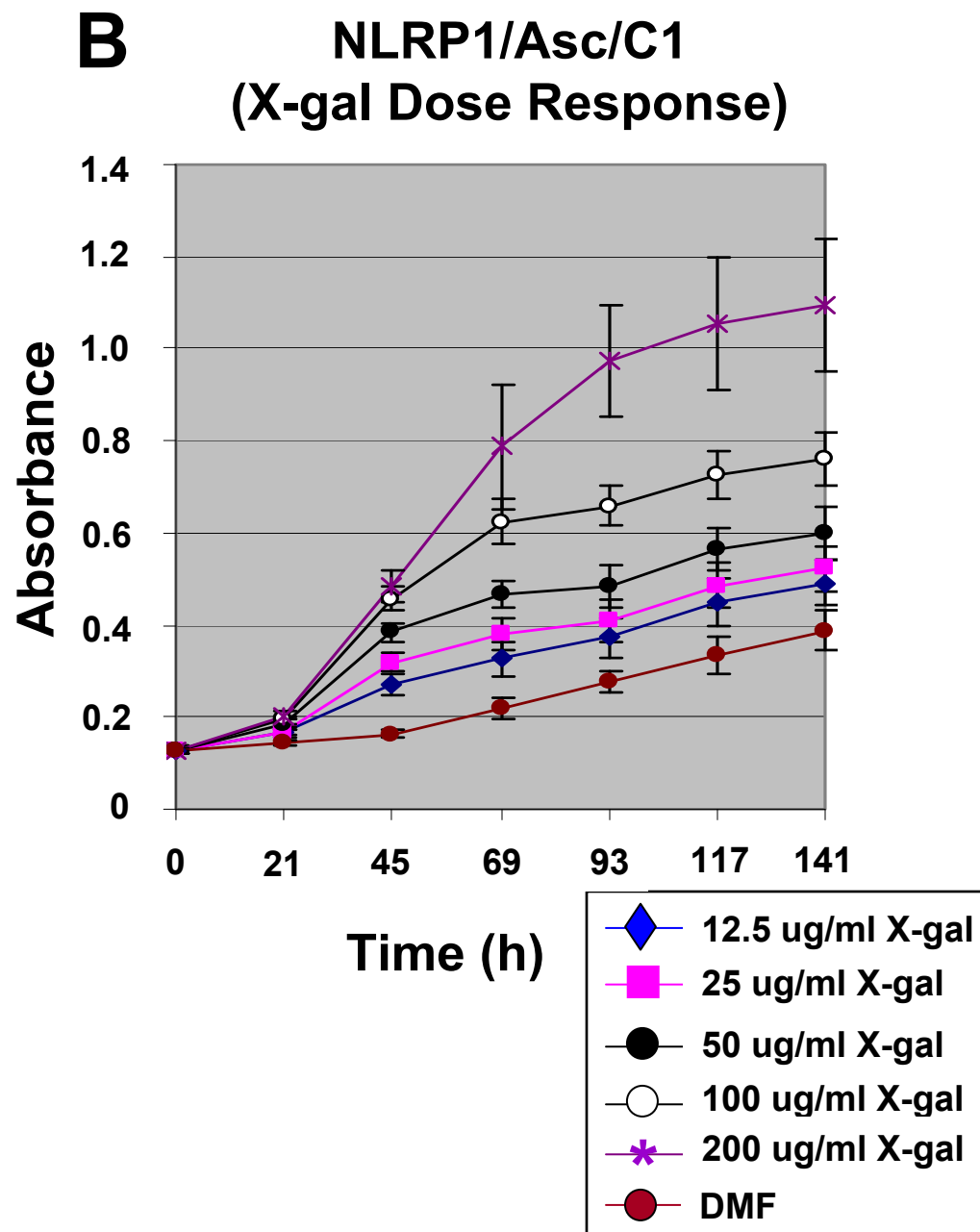

Figure-S17 (Reed)

Supplement: Figure S17 — Optimization of signal:noise ratio in microtiter plates: time and X-gal concentration. (A) EGY48 cells containing the 3-component Fas/FADD/Caspase-8 system and Caspase-8-cleavable reporter were grown at 2×10^5^ cells/well in 384 well plates, comparing β-galactosidase activity at various times (mean ± std dev; n = 3), for cells grown in the absence (black squares) or presence (circles) of X-gal and in the absence (circles) or presence (squares) of 100 µM zVAD-fmk Caspase inhibitor. (B) EGY48 cells containing the 3-component NLRP1ΔLRR/Asc/Caspase-1 system and Caspase-1-cleavable reporter were grown at 2×10^5^ cells/well in 384 well plates, comparing X-gal concentrations. The activity of β-galactosidase was measured after 72 hrs culture (mean ± std dev; n = 3) for cells grown in the absence (red circles) or presence (all others) X-gal. Various concentrations of X-gal were compared. (0.04 MB PDF) [file pone.0007655.s019.pdf]

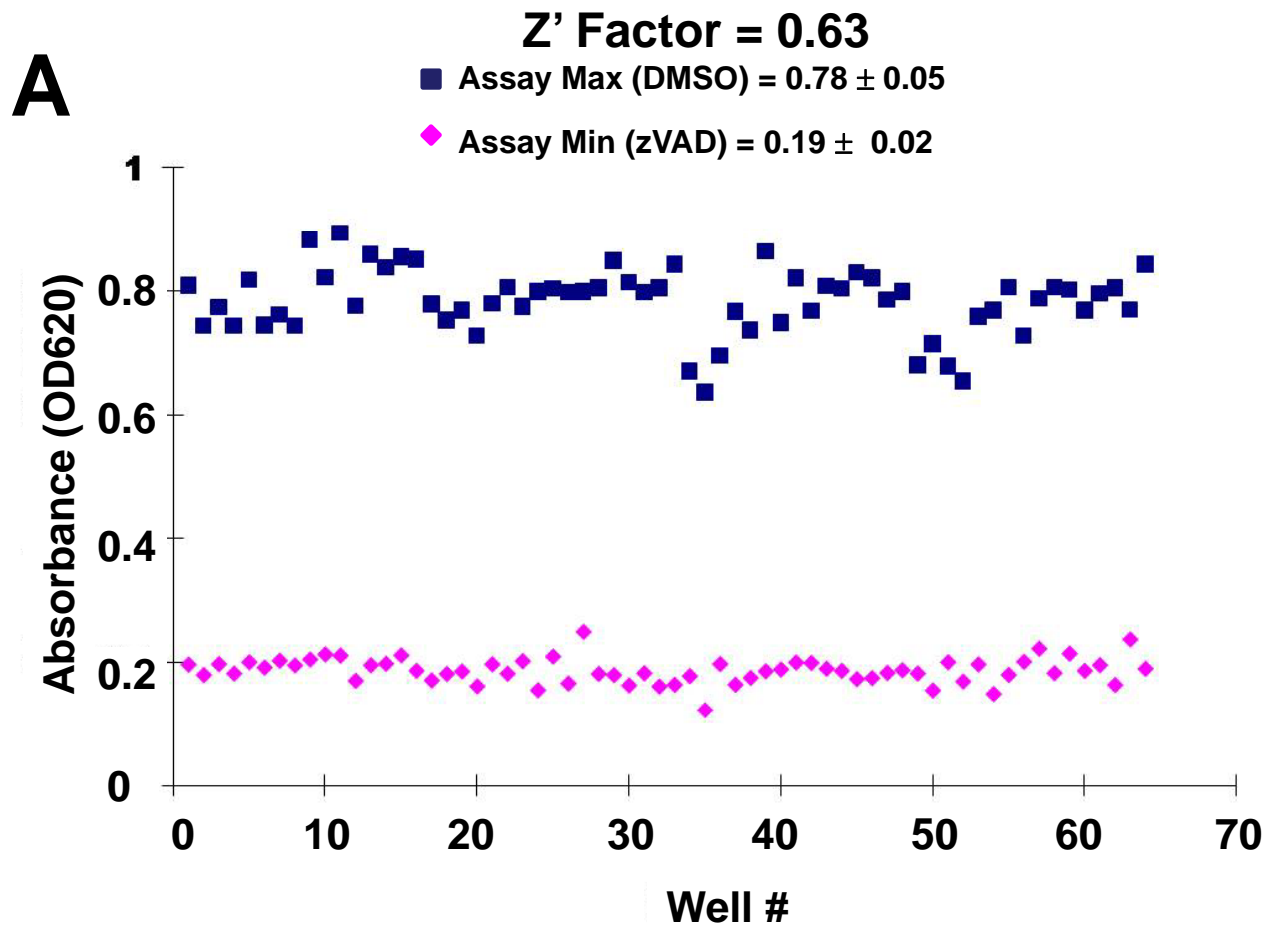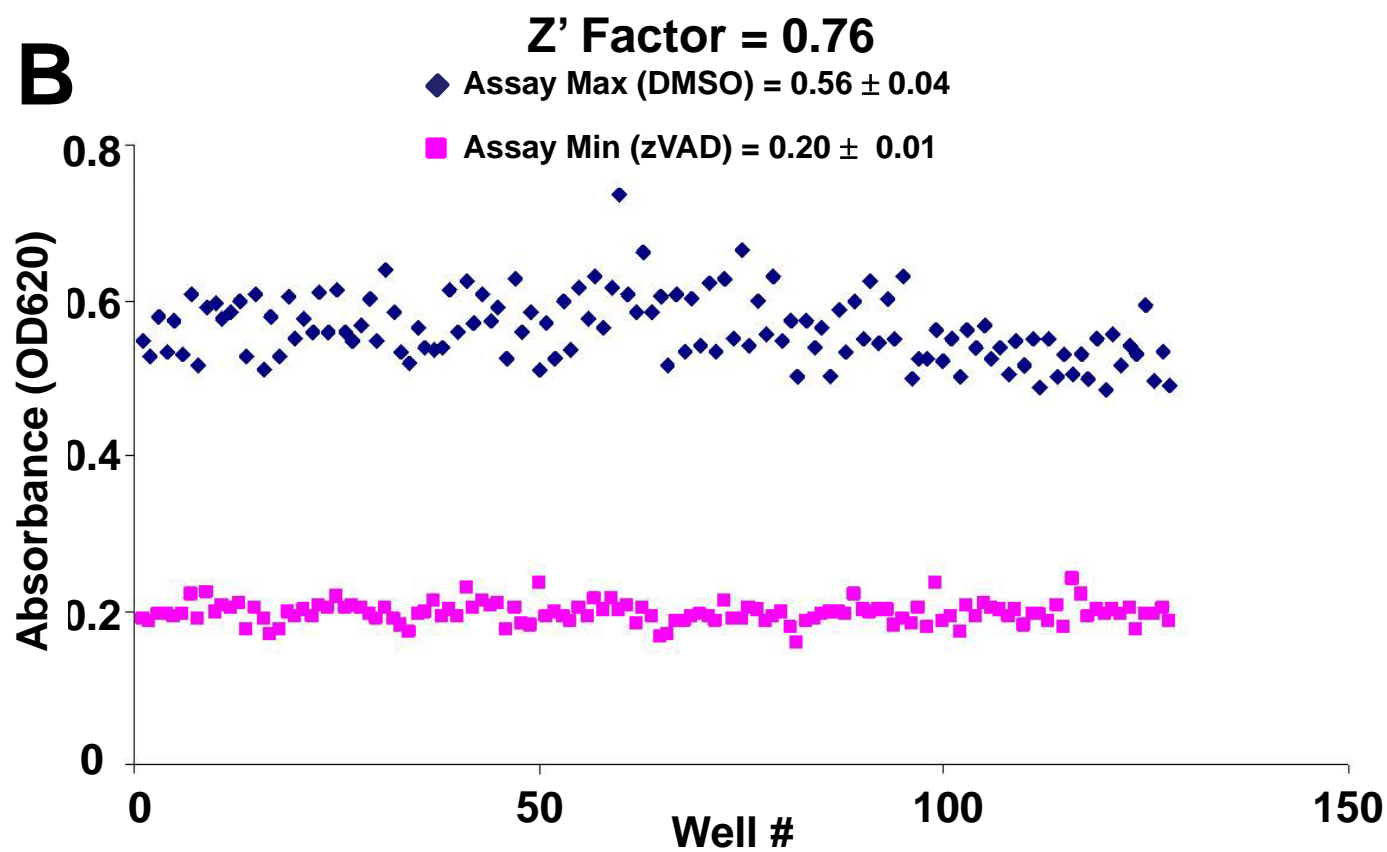

Figure S18 (Reed)

Supplement: Figure S18 — Determination of Z' scores for NLRP1(NALP1) and Fas(CD95) 3-component yeast-based HTS assays using caspase inhibitor zVAD-fmk. (A) Yeast strain EGY48 harboring the NLRP1/ASC/Caspase-1 proteolytic network (B) or the Fas/FADD/Caspase-8 proteolytic network were cultured at 2×10^5^ cells/well for 48 hours in SD media containing 400 µg/ml X-gal with DMSO solvent control (blue) or zVAD in DMSO (100 µM final concentration) (purple). The Z' scores were determined by comparison of the DMSO-treated samples with the zVAD-treated samples. (0.34 MB PDF) [file pone.0007655.s020.pdf]

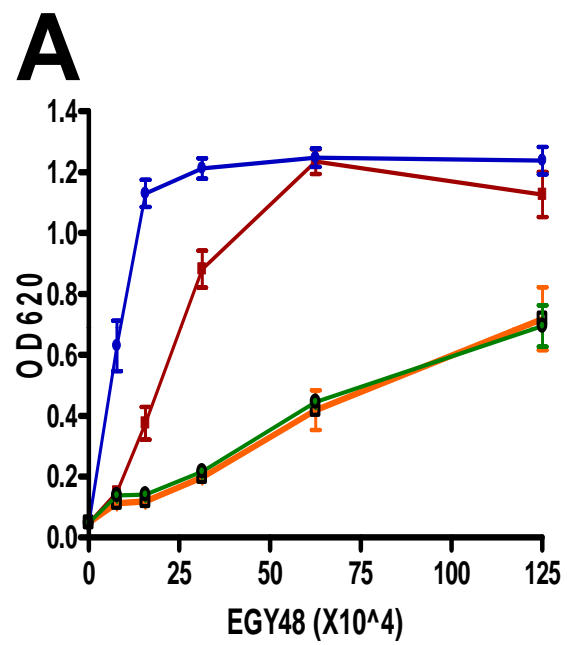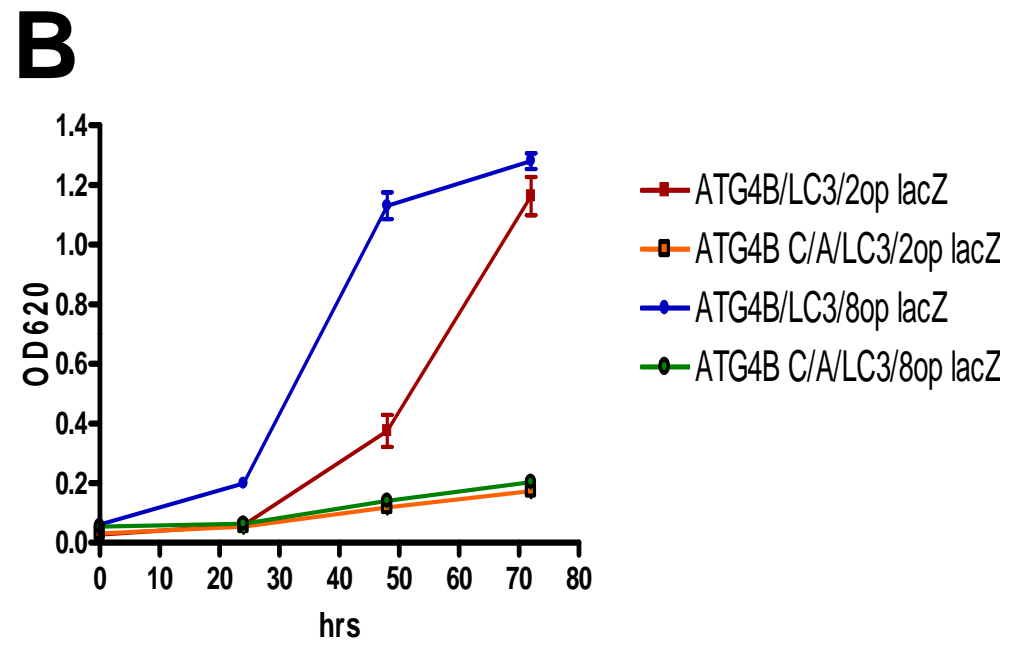

Figure S19 (Reed)

Supplement: Figure S19 — Experiments with ATG4B-expressing yeast. (A) Determination of cell density dependence. The production of β-galactosidase activity (y-axis) in 384 well plate format was compared for various densities of EGY48 cells (x-axis) harboring plasmids encoding ATG4B and the LC3-containing substrate and lacZ reporter genes containing either 2 (red, orange) or 8 (blue, green) LexA-operators. Medium consisted of 1% galactose/2%raffinos, with 100 µg/ml X-gal. Cells were cultured at 30°C for 48 hrs, then absorbance was measured at OD 620 nm. Data represent mean±std dev (n = 4). (B) The time-course of β-galactosidase activity generation was compared for EGY48 cells containing plasmids encoding LEU2 and lacZ reporter with (2 vs 8) LexA operators. Cells were plated and culture as described above. Data represent mean ± std dev (n = 4). (0.02 MB PDF) [file pone.0007655.s021.pdf]

**A**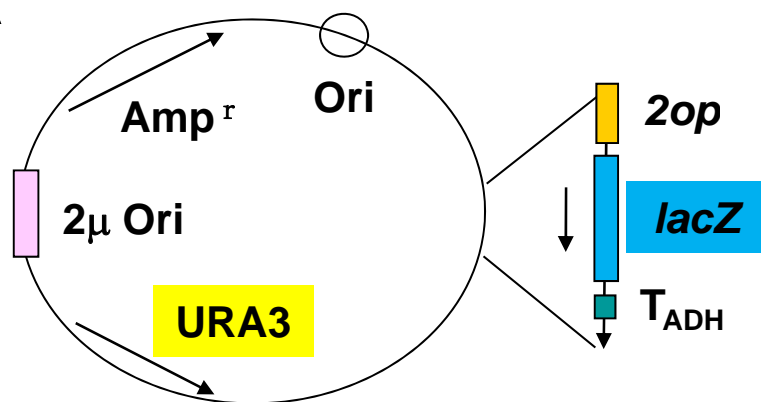**p426-2op-lacZ****B**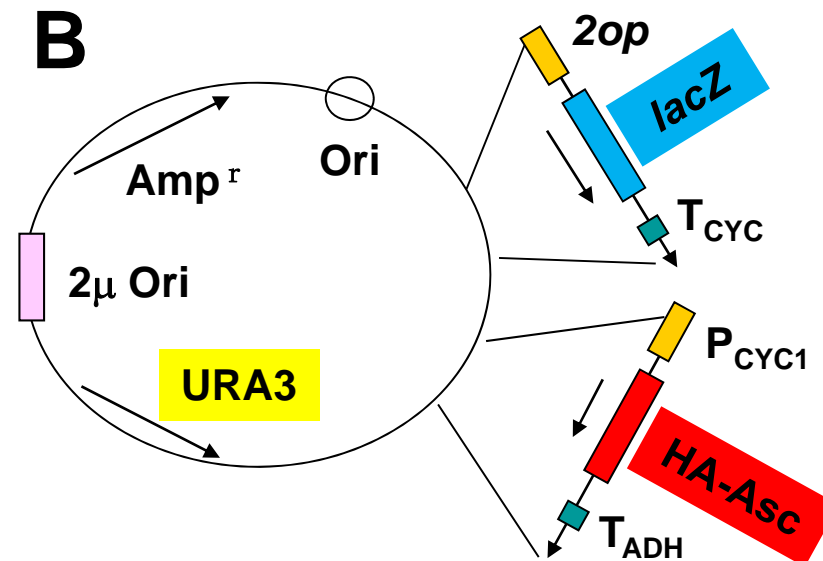**p426-2op-lacZ/CYC1-HA-Asc****C**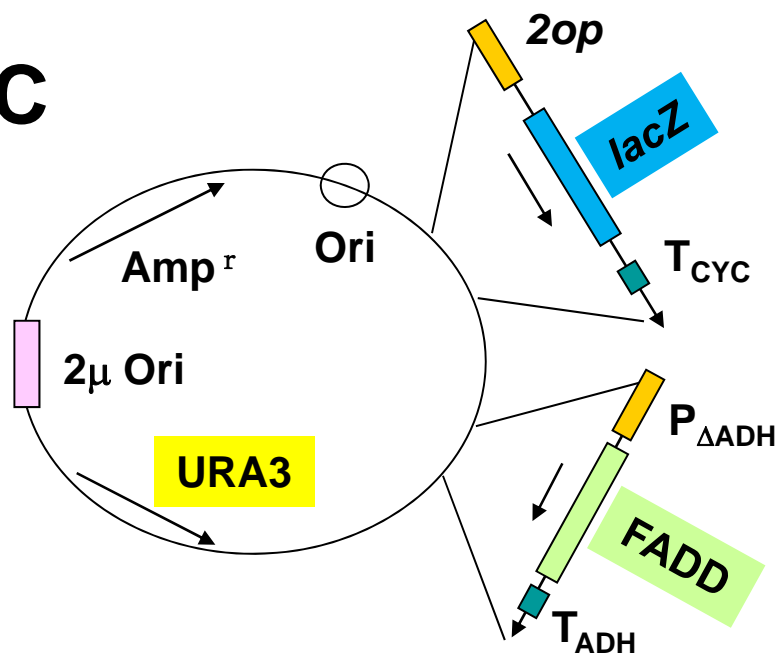**p426-2op-lacZ/ΔADH-FADD****D**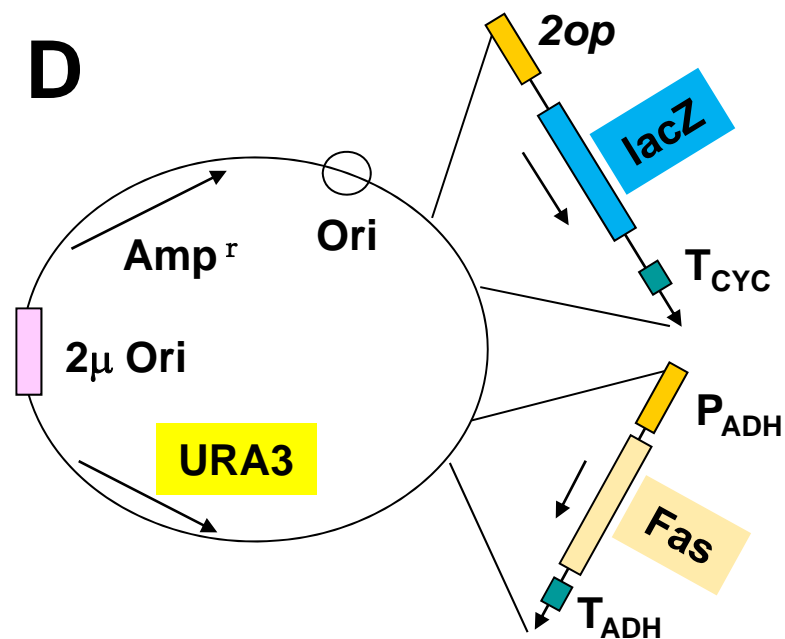**p426-2op-lacZ/ADH-Fas**

Supplement: Figure S21 — Plasmids for expression of upstream activators of Caspases and lacZ reporter gene in yeast. The plasmid p426 was used as the backbone for these constructions, containing 2µ plasmid origin for high-copy episomal replication in yeast and URA3 gene for selection in ura3 yeast strains. (A) The plasmid p426-2op-lacZ contains a lacZ gene driven by a minimal promoter containing two tandem copies of binding sites for the LexA transcription factor, followed by ADH gene termination element. (B) Plasmid p426-2op-lacZ/CYC1-HA-Asc contains an additional transcriptional unit, where expression of human cDNA encoding N-terminal HA-tagged Asc protein is driven by a CYC1 promoter, and followed by ADH gene termination element. (C) In plasmid p426-2op-lacZ/ΔADH1-FADD, expression of a human FADD cDNA is driven by ΔADH1 promoter and followed by ADH termination element. (D) Plasmid p426-2op-lacZ/ADH-Fas contains a human Fas(CD95) cDNA driven by ADH promoter, and followed by CYC1 gene termination element. (0.05 MB PDF) [file pone.0007655.s023.pdf]

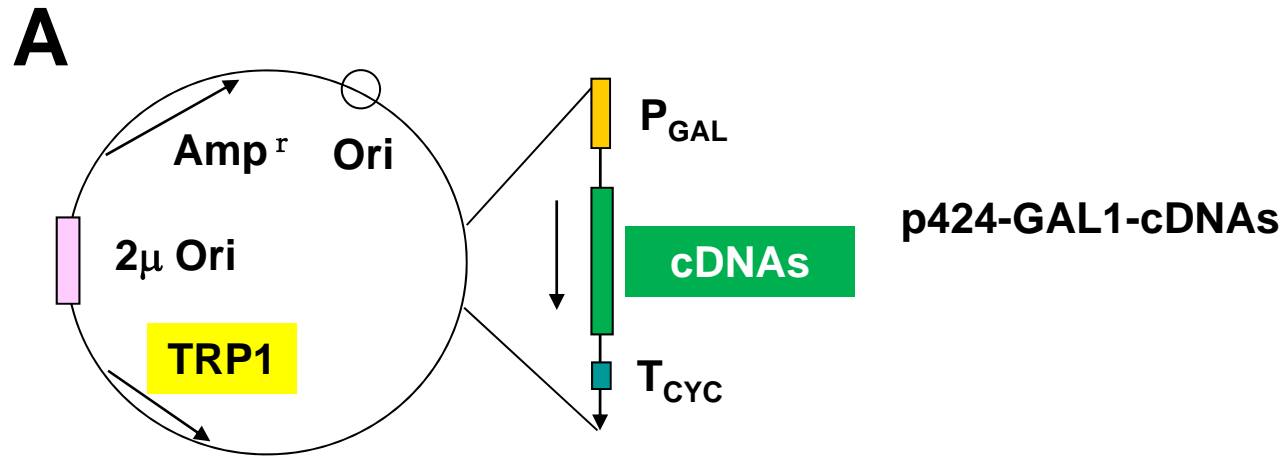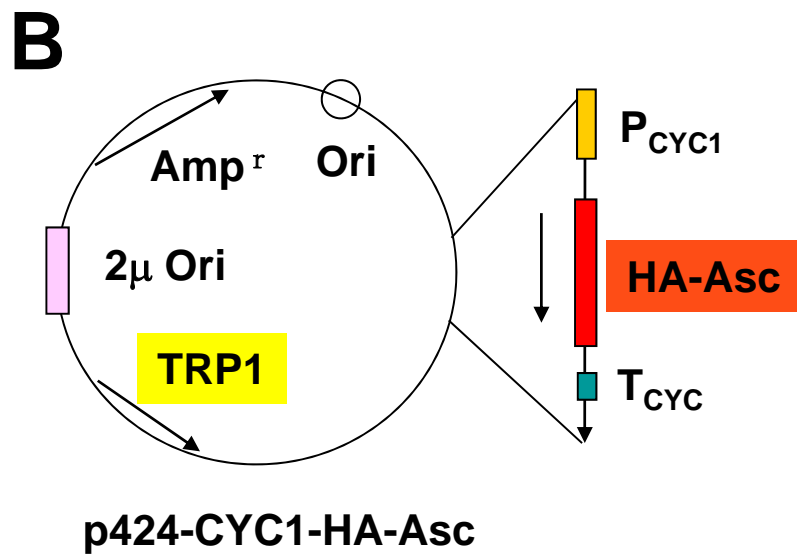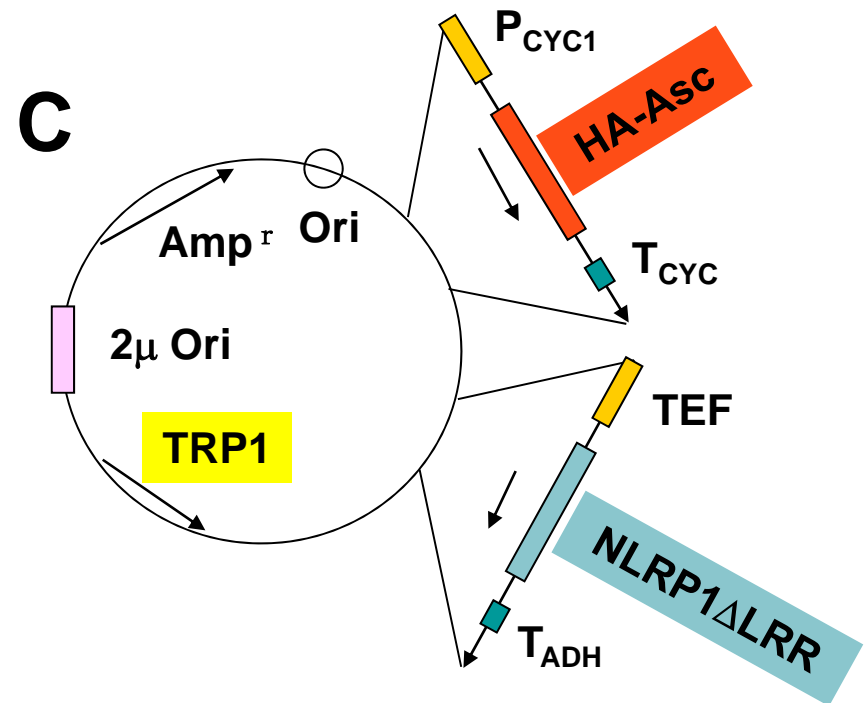

Figure-S22 (Reed)

Supplement: Figure S22 — Yeast expression plasmids for functional screening of cDNA libraries and expression of upstream activators of Caspases. The plasmid p424 was used as the backbone for these constructions, containing 2µ plasmid origin for high-copy episomal replication in yeast (S. cerevisiae) and TRP1 gene for selection in trp1 yeast strains. (A) The plasmid p426-GAL1-cDNAs contains human cDNAs directionally cloned downstream of a GAL1 promoter, followed by CYC1 gene termination element. (B) Plasmid p424-CYC1-HA-Asc contains a human cDNA encoding N-terminal HA-tagged Asc protein cloned downstream of a CYC1 promoter and followed by CYC1 gene termination element. (C) In plasmid p424-CYC1-HA-Asc/TEF-NLRP1ΔLRR, an additional transcriptional unit was added to p424-CYC1-HA-Asc above, where expression of a human cDNA encoding a gain-of-function NLRP1 mutant lacking LRRs (Faustin, B, et al. Molecular Cell 25;713, 2007) is driven by a TEF promoter and followed by ADH gene termination element. (0.04 MB PDF) [file pone.0007655.s024.pdf]
