# Supplementary material for: Versatile Assays for High Throughput Screening for Activators or Inhibitors of Intracellular Proteases and Their Cellular Regulators
Source: PLoS One. 2009 Oct 30;4(10):e7655. doi: 10.1371/journal.pone.0007655 (PMC2764853; doi:10.1371/journal.pone.0007655)
Supplement: Figure S6 — Validation of yeast-based assays for initiator Caspase activators: two-component systems. Yeast EGY48 strain containing 6op-LEU2/2op-lacZ was employed for developing 2-component assays for activators of upstream initiator Caspases (e.g. Caspases-1, 2, 8, 9, 10). Two independent clones of yeast transformants were plated on leucine-deficient medium containing X-gal. Yeast were transformed with plasmids encoding various membrane tethered transcription factor substrates containing (A) WEHD (“S1”), (B) DEHD (“S2”), (C) LEHD (“S9”) or (D) LETD (“S8”) cleavable linkers or their corresponding non-cleavable glycine mutants (“G1”, “G2”, “G9”, “G8”). The yeast were also transformed with plasmids encoding wild-type (WT) or catalytically inactive mutants of proforms various initiator Caspases expressed from relatively weak promoters (e.g., CYC1; ΔTEF3), including (A) pro-Caspase-1, (B) pro-Caspase-2, (C) pro-Caspase-9, and (D) pro-Caspase-10. (Note that the optimal betrapeptide sequences are the same for Caspase-8 and 10). These yeast were transformed with empty vectors (-) or plasmids encoding upstream activators of the Caspases, including (A) Asc, (B) RAIDD, (C) Apaf-1*, and (D) FADD, expressed from strong promoters (either GPD or TEF). Note that the lacZ reporter gene was activated only when the combination of an initiator Caspase and upstream activator was co-expressed, along with a cleavable substrate. Transformants: The transformed yeast cell clones are: (A) EGY48-6op-LEU2/2op-lacZ/TEF-Fas-d-S1(WEHD)-TA/ΔTEF3-Caspase1-FLAG (S1,C1(WT)), EGY48-6op-LEU2/2op-lacZ/TEF-Fas-d-G1(WEHG)-TA/ΔTEF3-Caspase1-FLAG (G1,C1(WT)), or EGY48-6op-LEU2/2op-lacZ/TEF-Fas-d-S1(WEHD)-TA/ΔTEF3-Caspase1(C285→G285)-FLAG, (S1,C1(C285→G285)), were transformed with the plasmids encoding the activator Asc, or the empty vector (-); (B) EGY48-6op-LEU2/2op-lacZ/ΔTEF2-Fas-d-S2(DEHD)-TA/ΔGPD1-HA-Caspase2- FLAG(S2,C2(WT)), EGY48-6op-LEU2/2op-lacZ/ΔTEF1-Fas-d- G2(DEHG)-TA/ΔGPD1-HA-Caspase2-FLAG (G2,C2(WT)), or E [file pone.0007655.s008.pdf]

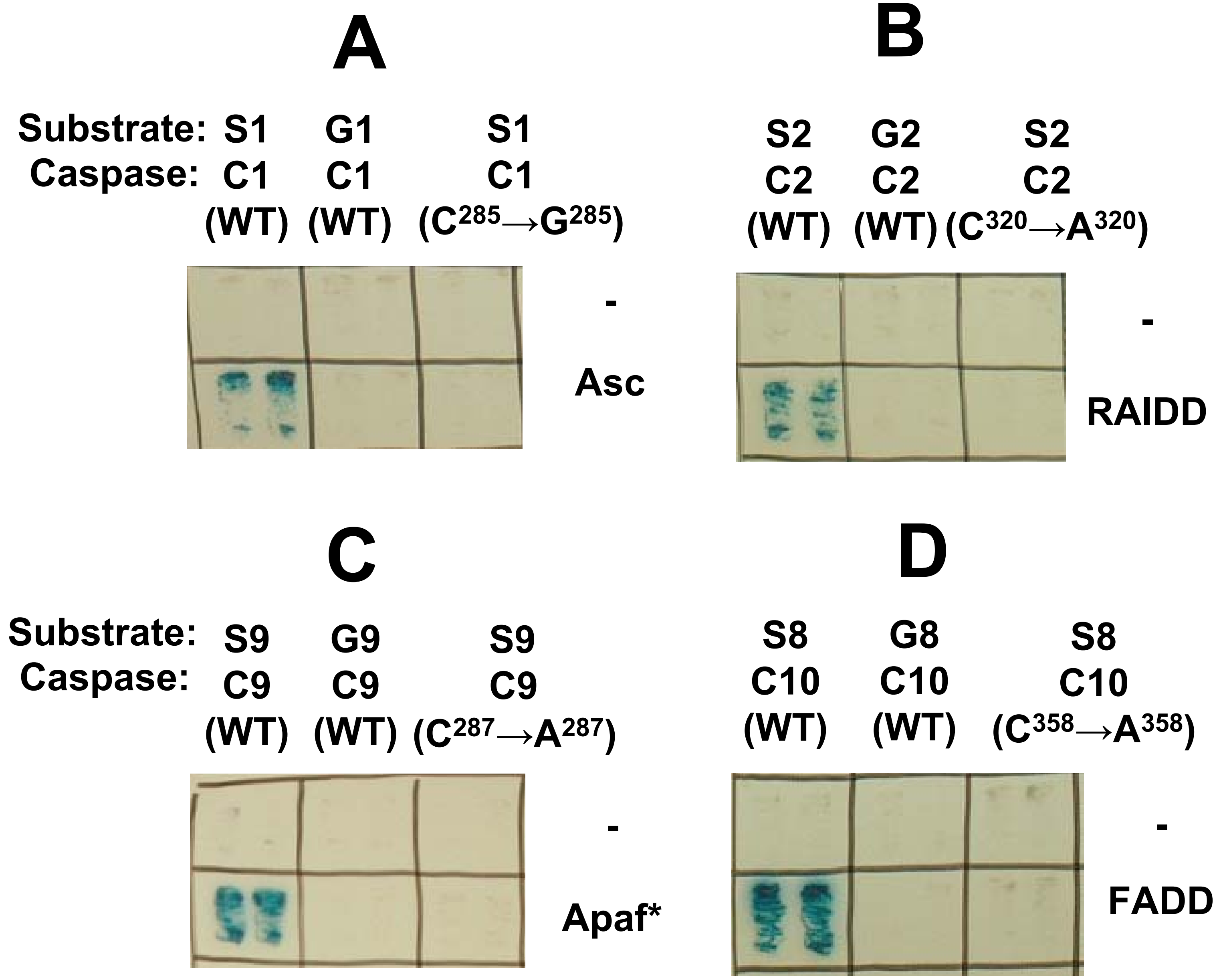

Figure-S6 (Reed)
