# Supplementary material for: Versatile Assays for High Throughput Screening for Activators or Inhibitors of Intracellular Proteases and Their Cellular Regulators
Source: PLoS One. 2009 Oct 30;4(10):e7655. doi: 10.1371/journal.pone.0007655 (PMC2764853; doi:10.1371/journal.pone.0007655)
Supplement: Figure S20 — Plasmids for co-expression of Caspases and substrate cleavable transcription factors in yeast. The plasmid p413 was used as the backbone for these constructions, containing CEN/ARS centromeric origin for low-copy episomal replication in yeast (S. cerevisiae) and HIS3 gene for selection in his3 yeast strains. (A) The plasmid p413-TEF-Fas-d-S1-TA, where expression of the Fas-LexA/B42 membrane tethered transcription factor with WEHD linker (Caspase-1/4/5 cleavable) is driven by the TEF promoter. (B) The plasmid p413-TEF-Fas-d-S1-TA/ΔTEF3-Caspase-1-FLAG, contains two additional transcriptional units, where expression of the Fas-LexA/B42 membrane tethered transcription factor with WEHD linker (Caspase-1/4/5 cleavable) is driven by the TEF promoter and expression of pro-Caspase-1 with C-terminal FLAG tag is driven by an attenuated TEF3 promoter (ΔTEF3). (C) The plasmid p413-TEF-Fas-d-S8-TA/CYC1-Caspase-8-HA contains two additional transcriptional units, where expression of the Fas-LexA/B42 membrane tethered transcription factor with LETD linker (Caspase-8/10 cleavable) is driven by the TEF promoter and expression of pro-Caspase-8 with C-terminal HA tag is driven by CYC1 promoter. (D) The plasmid p413-GDP-Fas-dS8-TA/CYC1-Caspase-10-FLAG, similarly contains two additional transcriptional units, where expression of the same Caspase-8/10 cleavable Fas-LexA/B42 substrate as above is driven by GDP promoter and where expression of pro-Caspase-8 with C-terminal FLAG tag is driven by CYC1 promoter. Transcriptional termination elements from the CYC1 and ADH genes were employed as illustrated. (0.07 MB PDF) [file pone.0007655.s022.pdf]

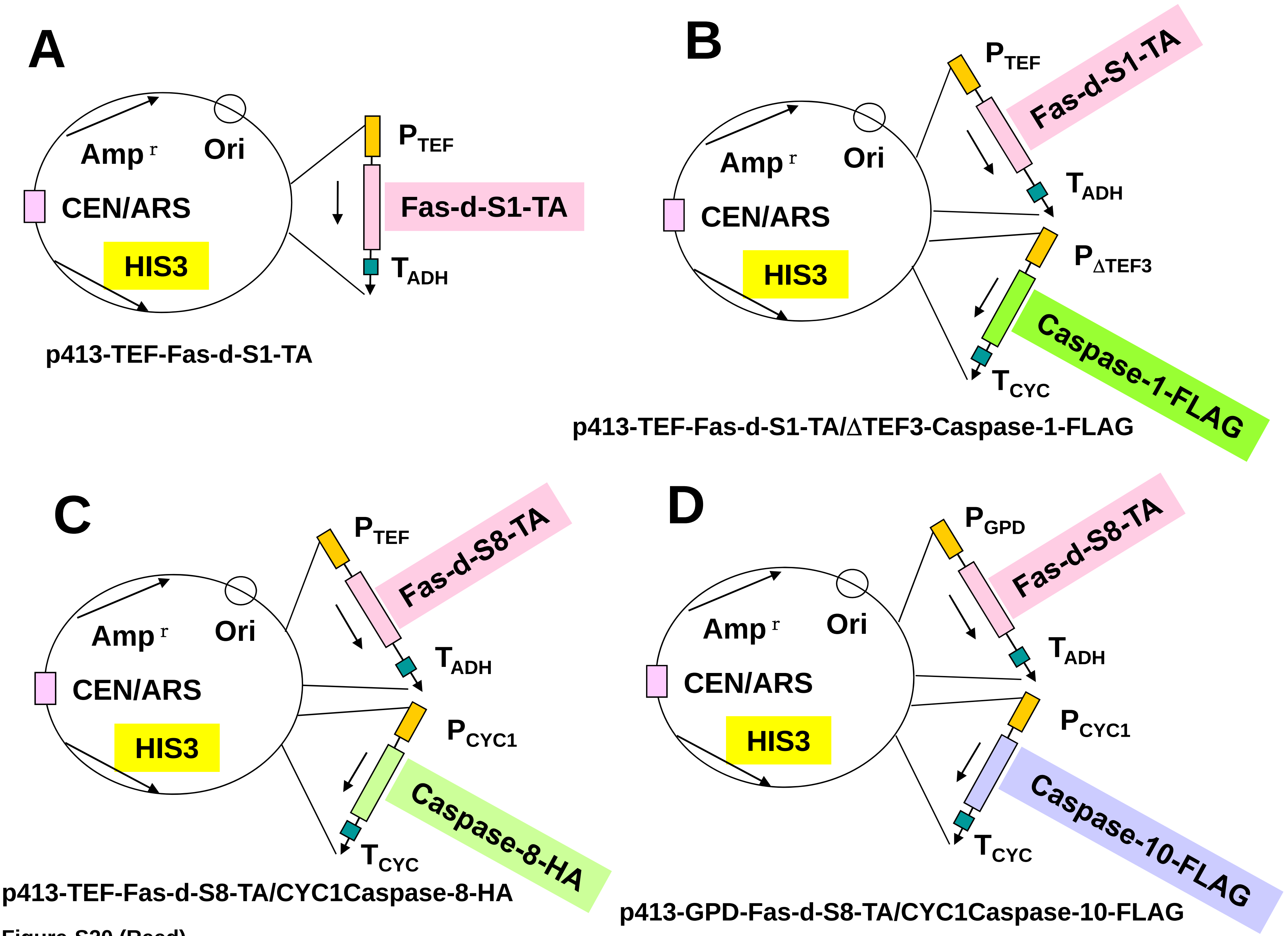

Figure-S20 (Reed)
